# RNA binding protein *Pcbp1* maintains mitochondria integrity to promote antibody production and germinal center response

**DOI:** 10.1126/sciadv.adz9095

**Published:** 2026-04-10

**Authors:** Lizhen Zhu, Li Lin, Tuan Qi, Geng Li, Zhixing Liang, Jiancheng Yu, Yifan Fu, Yue Peng, Xing Chang

**Affiliations:** ^1^ State Key Laboratory of Gene Expression, School of Medicine, Westlake University, Hangzhou, Zhejiang, China.; ^2^ School of Life Sciences, Westlake University, Hangzhou, Zhejiang, China.; ^3^ Fudan University, 220 Handan Road, Shanghai 200433, China.; ^4^ Institute of Basic Medical Sciences, Westlake Institute for Advanced Study, Hangzhou, Zhejiang, China.; ^5^ Center for Genome Editing, Westlake Laboratory of Life Sciences and Biomedicine, Hangzhou, Zhejiang, China.; ^6^ Research Center for Industries of the Future, Westlake University, Hangzhou, Zhejiang, China.; ^7^ Affiliated Hangzhou First People’s Hospital, School of Medicine, Westlake University, Hangzhou, Zhejiang, China.

## Abstract

B cells are crucial for adaptive immunity, orchestrating humoral responses by producing antibodies essential for pathogen clearance. Here, we show that Poly(rC) binding protein 1 (*Pcbp1*), a multifunctional RNA binding protein, is a key regulator of antibody production in B cells. *Pcbp1* deficiency in B cells resulted in significant reductions in immunoglobulin M expression at steady state and compromised differentiation of germinal center B cells and production of high-affinity antibodies upon immunization. These effects were caused by defective mitochondrial integrity in *Pcbp1*-deficient B cells, including impaired mitochondrial electron transport chain complex I and elevated mitochondrial reactive oxygen species production. Mechanistically, *Pcbp1* binds to the 3′ untranslated region of *Fdxr* messenger RNA to promote its expression, thereby supporting iron-sulfur cluster biogenesis, the assembly of mitochondrial complex I, and other Fdxr-dependent processes. Our findings reveal a previously unidentified role for *Pcbp1* in regulating mitochondrial function, protein synthesis, and antibody responses in B cells, providing insight into posttranscriptional regulation and mitochondrial functions in adaptive immunity.

## Teaser

RNA binding protein *Pcbp1* preserves mitochondrial integrity for efficient antibody production and germinal center formation.

## INTRODUCTION

B cells are critical components of the adaptive immune system, primarily responsible for the production of antibodies (*1*). Naïve B cells initially produce low-affinity immunoglobulin M (IgM), but upon antigen stimulation, they proceed to enter germinal centers (GCs) (*2*, *3*), a process essential for producing class-switched, high-affinity antibodies (*3*). These antibodies, produced by both naïve B cells and through GC responses, constitute the body’s first line of defense against invading pathogens. Recent studies have increasingly recognized the importance of metabolic regulation and mitochondrial dynamics in the differentiation of GC B (GCB) cells (*4*–*7*). However, the upstream regulatory mechanisms and their roles in naïve B cells remain less understood. Understanding these complex interactions could provide valuable insights into the regulation of antibody production and humoral immunity.

Mitochondria, beyond their well-known role in energy production, are involved in various cellular processes, including the generation of reactive oxygen species (ROS) as by-products of the electron transport chain (ETC) (*8*, *9*). While mitochondria-derived ROS can act as signaling molecules that influence B cell differentiation (*10*), excessive ROS production may compromise cell viability and contribute to functional defects. The precise mechanisms through which mitochondria-derived ROS are regulated in B cells and their impact on humoral immunity remains insufficiently understood.

Poly(rC) binding protein 1 (*Pcbp1*), also known as hnRNPE1, is a member of the heterogeneous nuclear ribonucleoprotein family. *Pcbp1* is ubiquitously expressed and participates in both transcriptional and posttranscriptional regulation (*11*–*13*). Moreover, *Pcbp1* also functions as an iron chaperone for other iron-containing proteins (*14*, *15*). Previous studies have implicated the importance of *Pcbp1* in antiviral response (*16*, *17*), tumor metastasis, and tumor suppression (*18*, *19*). *Pcbp1* has been found to stabilize proinflammatory cytokine mRNA (*20*) and to inhibit the conversion of effector T cells into regulatory T cells (*21*), thereby highlighting its pivotal role in the effector function of lymphocytes. Despite these findings, the contribution of *Pcbp1* in humoral immunity remains to be explored.

In this study, we reveal that *Pcbp1* is critical for maintaining the integrity of the mitochondrial ETC, thereby promoting antibody production and efficient GC responses. We demonstrate that *Pcbp1* deficiency in naïve B cells impairs mitochondrial ETC function, leading to a global suppression of protein translation, including IgM production. In GCB cells, *Pcbp1* deficiency disrupts light zone (LZ) formation, resulting in compromised GCB differentiation and a less efficient production of high-affinity antibodies. Mechanistically, *Pcbp1* interacts with mRNA of *Fdxr,* a key protein in iron-sulfur (FeS) cluster biogenesis and critical for ETC complex I assembly, to enhance *Fdxr* expression and mitigate the excessive production of mitochondrial ROS (mt-ROS). Our findings uncover a previously unrecognized link between posttranscriptional regulation and mitochondrial dynamics in the humoral immune response.

## RESULTS

### *Pcbp1* promotes IgM production

*Pcbp1* is abundantly expressed across all stages of B cell development, with the highest levels observed at the pro-B stage under steady state (fig. S1A). Following naïve B cell differentiation into GC B cells, *Pcbp1* expression was further elevated to a level even higher than that of pro-B cells (fig. S1, B and C). To circumvent embryo lethality (*22*) and analyze the function of *Pcbp1* in B cells, we generated *Pcbp1* floxed (fl/fl) Mb1-Cre^+/−^ mice (hereafter referred to as *Pcbp1* BKO), where conditional *Pcbp1* alleles were deleted by Mb1-Cre (*23*) during the transition from pre–pro B cells to pro-B cells. Intracellular staining confirmed selective depletion of *Pcbp1* in splenic mature B cells, with no observable effects in non–B cell compartment (CD19^−^B220^−^) (fig. S1D).

In the developing B cells at the bone marrow, *Pcbp1* deficiency resulted in a notable blockade in the transition from pro-B to pre-B cells (fig. S1, E to G). Pre-B cells (CD25^+^c-kit^−^) showed reduced percentages (from 61.2 to 36.2%) in *Pcbp1* BKO mice, and, concurrently, the percentage of pro-B cells (CD25^−^c-Kit^+^) was increased in the *Pcbp1* BKO bone marrow (from 9.10 to 17.1%) (fig. S1, E and F). The number of B cells in the bone marrow of *Pcbp1* BKO mice was reduced, with the pre-B cells being the major affected population (fig. S1G), indicating a blockade from pro-B cells to pre-B cells in the absence of *Pcbp1*. Recirculating IgM^+^IgD^+^CD19^+^ B cells were also decreased (from 29.1 to 10.4%) in the *Pcbp1* BKO mice (fig. S1, H and I).

Despite the notable blockade of B cell development in the bone marrow, total numbers (fig. S1J) and ratios of B cells (fig. S1, K and L) were minimally different in the spleen between 7-week-old *Pcbp1* BKO mice and wild-type (WT) mice. With the increase of age, total B cells in the spleen of *Pcbp1* BKO mice showed a minor reduction compared to their WT littermates (fig. S1M). Like the spleen, B cells in other peripheral lymphoid organs of *Pcbp1* BKO mice, including Peyer’s patches, peripheral lymph nodes, and mesenteric lymph nodes, were slightly reduced (fig. S1, K and L). Further examination revealed a minor reduction in the proportion of mature B cells (CD19^+^CD93^−^) (from 39.5 to 25.3%; fig. S1, N and O) in the spleen and a decrease of marginal zone B (MzB) cells (fig. S2, A and B), suggesting that *Pcbp1* was largely dispensable for mature B cell homeostasis, a function likely to be compensated by other genes (e.g., *Pcbp2*). B1a cells in the peritoneal cavity were also reduced (fig. S2, C and D). Notably, two nonexclusive factors may reconcile the observed partial block of B cell development in the bone marrow with the near-normal mature B cell compartment: The spleen contains a limited number of survival niches that cap peripheral B cell numbers, and mature splenic B cells are long-lived with slow turnover.

**Fig. 1.**
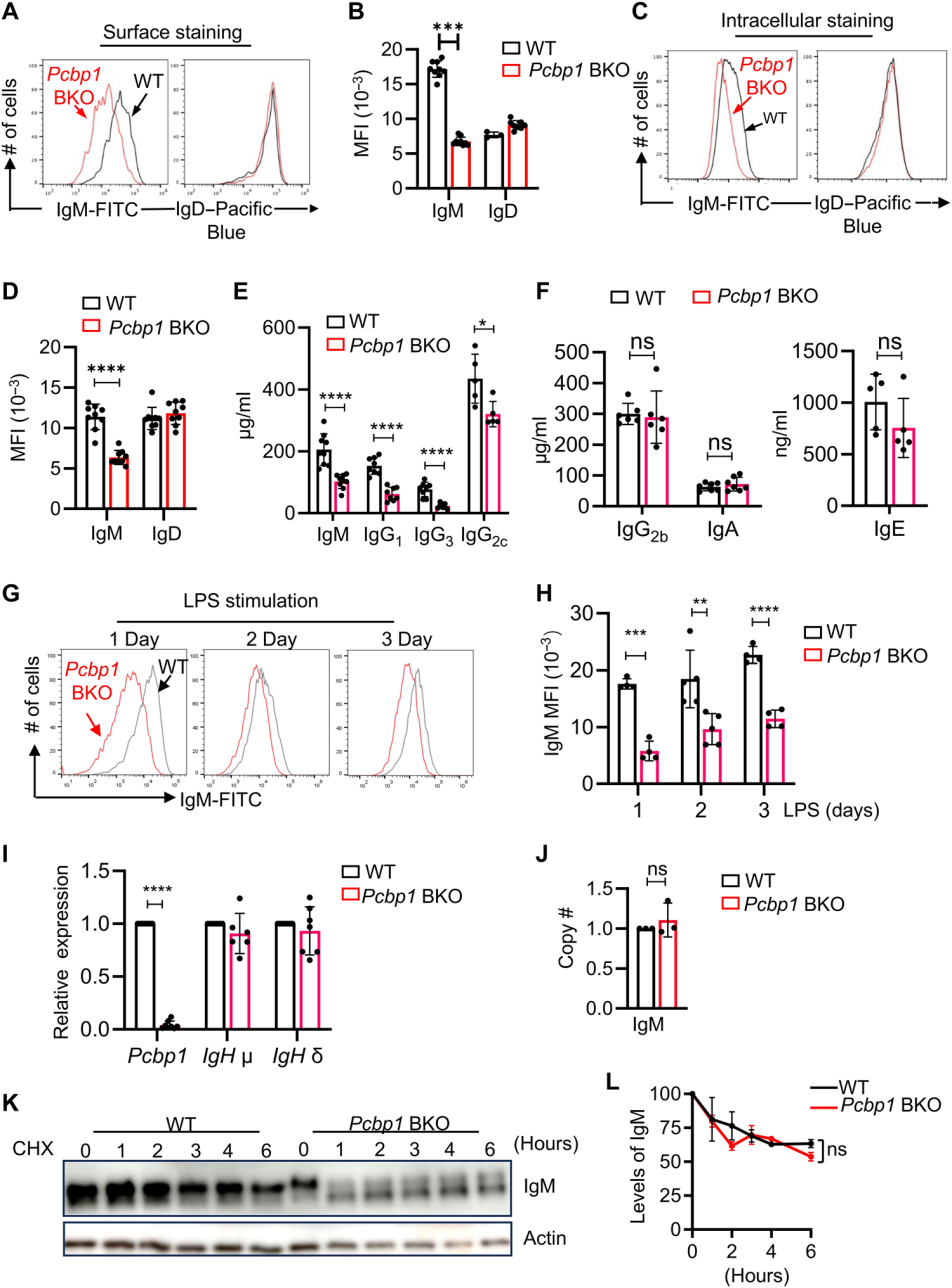
*Pcbp1* promotes IgM expression in B cells. (**A** and **B**). Loss of *Pcbp1* diminished levels of IgM but not IgD in naïve B cells. Naïve B cells in the spleen (CD19^+^B220^+^) were gated from 7-week-old Mb1-Cre *Pcbp1*^fl/fl^ (*Pcbp1* BKO) or WT littermates. Surface IgM and IgD levels were determined by flow cytometry. Data are representative (A) or summary (B) of three independent experiments. MFI, mean fluorescence intensity. (**C** and **D**). As in (A), total IgM levels in the splenic B cells were determined by intracellular staining. Data are representative (C) or summary (D) of three independent experiments. FITC, fluorescein isothiocyanate. (**E** and **F**). Serum levels of IgM, IgG, IgA, and IgE in 7-week-old WT or *Pcbp1* BKO mice were measured by enzyme-linked immunosorbent assay (ELISA). Data are a summary of five independent experiments. ns, not significant. (**G** and **H**). Naïve B cells from WT or *Pcbp1* BKO mice were stimulated with LPS, IL-2, and IL-5, and IgM expression was determined at indicated time following the stimulation via surface staining. Data are representative (G) or summary (H) from three independent experiments. (**I**). *Igh*μ and *Igh*δ levels in WT and *Pcbp1* BKO naïve B cells were determined via real-time PCR. Data are summary from at least three independent experiments. (**J**). The *Igh*μ copy number in naïve B cells was quantified using digital PCR. Data are summarized from three independent experiments. (**K** and **L**). *Pcbp1* did not affect IgM protein stability. Naïve B cells from WT and *Pcbp1* BKO mice were stimulated with LPS, IL-2, and IL-5. After 24 hours, cycloheximide (CHX) was added to inhibit protein synthesis. IgM and actin protein levels were assessed by immunoblotting at the indicated time points following CHX treatment (K). (L) Levels of IgM protein were normalized to actin. Data are presented as means ± SD, *P < 0.05, **P < 0.01, ***P 0.001, ****P < 0.0001 in Student’s *t* test.

Naïve mature B cells predominantly express surface IgM and IgD, with IgD generated via alternative splicing of the μ Ig heavy chain transcripts (*24*). Despite a generally intact B cell compartment in the spleen of *Pcbp1*-deficient mice, flow cytometric analysis revealed a more than 50% reduction of surface IgM expression on splenic B cells, whereas surface IgD levels were unaltered (Fig. 1, A and B). Intracellular staining demonstrated a similar reduction in total cellular IgM levels in *Pcbp1*-deficient B cells (Fig. 1, C and D). To exclude the potential confounding effects associated with Mb1 Cre allele—generated by replacing Cd79a with a Cre transgene—we compared IgM expression between Mb1-Cre^+/−^ *Pcbp1*^+/+^ and Mb1-Cre^+/−^ *Pcbp1*^fl/fl^ mice (fig. S2E). A similar reduction was observed in B cells from the latter mice. Furthermore, IgM expression was comparable between WT (Mb1-Cre^−/−^) and Mb1-Cre^+/−^ mice (fig. S2E), confirming that the phenotype is due to *Pcbp1* loss rather than Cre-mediated effects.

Both splenic follicular B (FoB) cells and MzB cells exhibited diminished IgM expression in *Pcbp1* BKO mice compared to their counterparts in the WT mice (fig. S2, F and G). However, only *Pcbp1*-deficient MzB cells, but not FoB cells, displayed drastic elevated IgD levels (fig. S2, H and I). Lymph node B cells, which are predominantly follicular, also displayed reduced IgM expression in *Pcbp1* BKO mice (fig. S2, J and K), as did B1a cells (fig. S2, L and M). These observations demonstrate that *Pcbp1* is essential for maintaining steady-state IgM expression across multiple B cell subsets.

Furthermore, serum levels of IgM, IgG1, IgG3, and IgG2c were decreased by approximately 50% in *Pcbp1* BKO mice (Fig. 1E). In contrast, the other isotypes, including IgA, IgE, and IgG2b in the serum were not altered in the *Pcbp1* BKO mice (Fig. 1F). The concomitant decrease in multiple Ig isotypes also suggests a shared mechanism governing the expression of these Ig isotypes and the involvement of *Pcbp1* in GC responses (discussed in further detail below).

### *Pcbp1* promotes Ig expression in activated B cells

As a prototype of T-independent antigen, lipopolysaccharide (LPS) stimulates B cell activation, proliferation, and up-regulation of IgM expression and secretion, a process critical for the immediate and robust neutralization of pathogenic bacteria (*25*). Consistent with our observations in naïve B cells, *Pcbp1*-deficient B cells exhibited reduced IgM expression following LPS stimulation in vitro (Fig. 1, G and H). In addition to IgM, LPS-induced IgG3 expression was reduced in *Pcbp1* BKO cells in vitro (fig. S3, A to C).

To further establish a cell-autonomous role for *Pcbp1* in regulating IgM expression, we depleted *Pcbp1* using multiple orthogonal approaches. First, *Pcbp1* was deleted in vitro by Cre-expressing retrovirus in *Pcbp1^flox/flox^* B cells (fig. S3D), as well as by in vitro activation of Aid-Cre *Pcbp1^flox/flox^* primary B cells (fig. S3E). In parallel, *Pcbp1* was knocked down by short hairpin RNA (shRNA) in Namalwa cells, a human IgM-expressing B lymphoma cell line (fig. S3F). Across all approaches, acute *Pcbp1* depletion in vitro consistently resulted in reduced IgM levels. These findings corroborate our observations in vivo and support a conserved, cell-intrinsic role for *Pcbp1* in regulating IgM expression in both murine and human B cells. Despite the substantial reduction in IgM protein, the levels of IgM transcripts were similar between WT and *Pcbp1* BKO naïve B cells, as shown by quantitative real-time polymerase chain reaction (PCR) and digital PCR analyses (Fig. 1, I and J). The levels of membrane and secreted IgM (μ chain) isoforms, as well as IgD (δ chain) were also comparable between WT and *Pcbp1* BKO B cells (fig. S3G). Similarly, IgM transcription following the LPS stimulation was not impaired in the *Pcbp1* BKO B cells (fig. S3H). In addition, no difference in IgM protein degradation was observed between WT and *Pcbp1*-deficient B cells when cycloheximide was used to block new protein synthesis (Fig. 1, K and L). These findings indicate that *Pcbp1* promotes IgM and IgG production through posttranscriptional mechanisms, independent of effects on RNA transcription or protein stability.

### *Pcbp1* maintains mitochondria integrity in naïve B cells to sustain protein translation

**Fig. 2.**
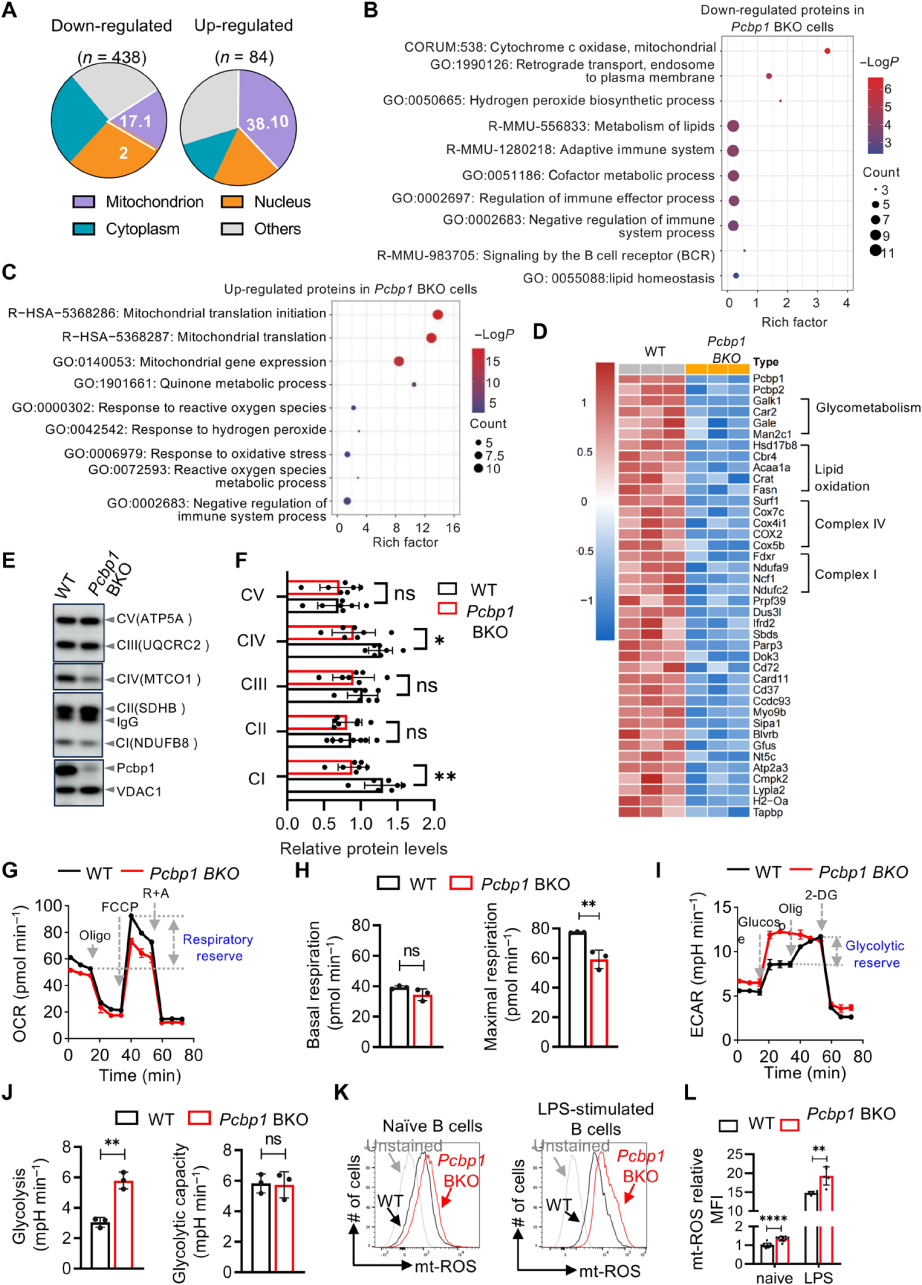
*Pcbp1* regulates mitochondrial dynamics in B cells. (**A** to **D**). Mass spectrometer analysis of naïve B cells derived from *Pcbp1* BKO or WT littermates. Differentially expressed proteins were identified using TMT mass spec with criteria of |log_2_FC| ≥ 0.5 and *P* < 0.05. (A). Subcellular localization of down-regulated (left) and up-regulated (right) proteins in *Pcbp1*-deficient B cells. Data were curated on the basis of UniProt annotations. [(B) and (C)]. GO analysis of down-regulated proteins (B) or up-regulated proteins (C) in *Pcbp1*-deficient B cells using Metascape (*65*). (D). Heatmap illustrating down-regulated proteins in *Pcbp1*-deficient B cells compared to WT cells. (**E** and **F**). Levels of mitochondrial respiratory complexes were determined by immunoblotting via total OXPHOS antibody cocktail against complexes I to V. Data are representative (E) or summary (F) of five independent experiments. (**G** and **H**). *Pcbp1*-deficient naïve B cells exhibited reduced OCR. Purified splenic B cells were analyzed for OCR using a Seahorse XFe96 extracellular flux analyzer. Representative trace (G) and quantitative analyses of basal/maximum OCR (H) from two independent experiments (*n* = 3 mice in each group). (**I** and **J**). As in (G), ECAR measurements were performed on *Pcbp1*-deficient and WT naïve B cells (4 × 10^5^ cells per well). Error bars stand for SD of the mean. Data are representative (I) and quantitative (J) of three independent experiments. (**K** and **L**). Loss of Pcbp1 increases mt-ROS in B cells. Naïve or stimulated B cells (LPS, IL-2, and IL-5 for 24 hours) were stained with MitoSOX. mt-ROS levels were analyzed by flow cytometry (K). Left, naïve B cells; right, LPS-stimulated B cells. (L) Quantification of mt-ROS levels, presented as means ± SD from five independent experiments. Error bars indicate SD of the mean, with statistical significance assessed using Student’s *t* test. *P < 0.05, **P < 0.01, ****P < 0.0001.

To gain mechanistic insight into how *Pcbp1* regulates IgM expression, we comprehensively profiled proteins affected by *Pcbp1* deficiency in naïve B cells using quantitative tandem mass tagging (TMT) proteomics (*26*). A total of 438 proteins were reduced while only 84 proteins were increased in the *Pcbp1*-deficient cells compared to the WT cells (Fig. 2A). Notably, more than 17.2% of the decreased proteins were located in mitochondria (Fig. 2A), with most of them being nuclear-encoded mitochondrial proteins. Gene ontology (GO) analysis showed that down-regulated proteins in *Pcbp1*-deficient B cells were enriched for mitochondrial cytochrome c oxidase and hydrogen peroxide biosynthetic processes (Fig. 2B). In contrast, up-regulated proteins were enriched in pathways associated with mitochondrial translation and response to oxidative stress, likely reflecting a compensatory response to impaired oxidative phosphorylation (OXPHOS) (Fig. 2C). Components of complex I (e.g., *Fdxr* and *Ndufa9*) of the mitochondrial ETC were reduced in the *Pcbp1*-deficient B cells (Fig. 2D). Proteins involved in glycometabolism, lipid oxidation, and ETC complex IV were also reduced. The reduction in ETC complex I and complex IV in *Pcbp1*-deficient naïve B cells was further confirmed by immunoblotting (Fig. 2, E and F).

To evaluate the functional impact of these mitochondrial proteomic changes, we conducted Seahorse metabolic flux analysis on *Pcbp1*-deficient naïve B cells. This revealed compromised mitochondrial metabolism, with significantly reduced maximal oxygen consumption rate (OCR) following carbonyl cyanide *p*-trifluoromethoxyphenylhydrazone (FCCP) uncoupling compared to WT controls (Fig. 2, G and H), indicating impaired respiratory capacity. Conversely, extracellular acidification rate (ECAR) measurements revealed elevated glycolytic activity in *Pcbp1*-deficient B cells, particularly following the addition of saturating amounts of glucose (Fig. 2, I and J). These data thus indicate mitochondrial respiratory chain dysfunction in *Pcbp1*-deficient B cells, causing compensatory glycolytic up-regulation to maintain cellular energy homeostasis.

Naïve B lymphocytes have low energy requirement and rely on OXPHOS for adenosine triphosphate (ATP) generation (*27*). The TMT analysis (Fig. 2D) revealed reduced expression of proteins from both complex I and complex IV. We focused on complex I because it serves as the main entry point of electrons into the ETC, playing a critical role in the reduced form of nicotinamide adenine dinucleotide (oxidized form) (NAD^+^, NADH) oxidation, redox balance, and ATP/precursor production in B cells (*28*). Complex I is also the major source of mt-ROS, which regulate lymphocyte signaling and survival, whereas complex IV acts at the terminal ETC step, contributes minimally to ROS production, and is generally less rate-limiting under physiological conditions. *Pcbp1*-deficient naïve B cells exhibited a decreased NAD^+^/NADH ratio (fig. S3I), suggesting impaired ETC complex I activities. Despite this defect, ATP levels were slightly increased (fig. S3J), implying a metabolic shift from OXPHOS to glycolysis in the *Pcbp1*-deficient cells as shown in the ECAR results (Fig. 2I). A similar reduction in the NAD^+^/NADH ratio was also observed in LPS-stimulated cells (fig. S3K). This metabolic adaptation likely contributes to the minimal impact of *Pcbp1* deficiency on naïve B cell homeostasis.

Defective complex I can lead to an increased production of ROS (*29*–*31*), which may, in turn, inhibit cytosolic protein translation (*30*). *Pcbp1*-deficient naïve and LPS-activated B cells exhibited elevated mitochondrial (Fig. 2, K and L) and total ROS levels (fig. S3L) compared to their WT counterparts, indicating that *Pcbp1* deficiency induced oxidative stress in B cells. Consistent with this, *Pcbp1*-deficient naïve B cells displayed a 28.5% reduction in total cellular protein compared to WT controls (fig. S4A) without significantly affecting the size of cells (fig. S4B). To directly assess protein synthesis, we used the puromycin (Puro) incorporation assay (*32*). This revealed substantially lower Puro incorporation in *Pcbp1*-deficient follicular and MzB cells compared to their WT counterparts, indicating reduced nascent protein synthesis across B cell subsets (fig. S4, C and D). Such defects were also observed following LPS stimulation, where O-Propargyl-Puromycin (OP-Puro) incorporation (*32*) was significantly lower in *Pcbp1*-deficient B cells, as assessed by Western blotting (fig. S4E). These findings demonstrate that *Pcbp1* deficiency leads to increased ROS and a significant reduction in protein synthesis in B cells.

Collectively, these findings highlight the critical role of *Pcbp1* in preserving the integrity and function of ETC complex I in naïve B cells. Although *Pcbp1* deficiency did not significantly affect ATP production, it resulted in a notable reduction in NAD^+^/NADH level. This metabolic disruption increased oxidative stress and impaired protein translation, ultimately compromising IgM production.

### Restoration of mitochondrial function partially rescues IgM translation in *Pcbp1*-deficient B cells

To establish a causal link between mitochondrial dysfunction and impaired IgM expression, we first treated WT B cells with rotenone, which inhibits the transfer of electrons from FeS centers in complex I to ubiquinone and enhances production of ROS (*33*). Rotenone treatment recapitulated the effects of *Pcbp1* deficiency, including increased levels of mt-ROS (fig. S4F) (*33*), suppressed global protein translation (fig. S4G), and reduced IgM levels (fig. S4, H and I) in primary B cells, while leaving IgD and CD19 levels unaffected. No alterations were observed in IgM mRNA levels (fig. S4J).

**Fig. 3.**
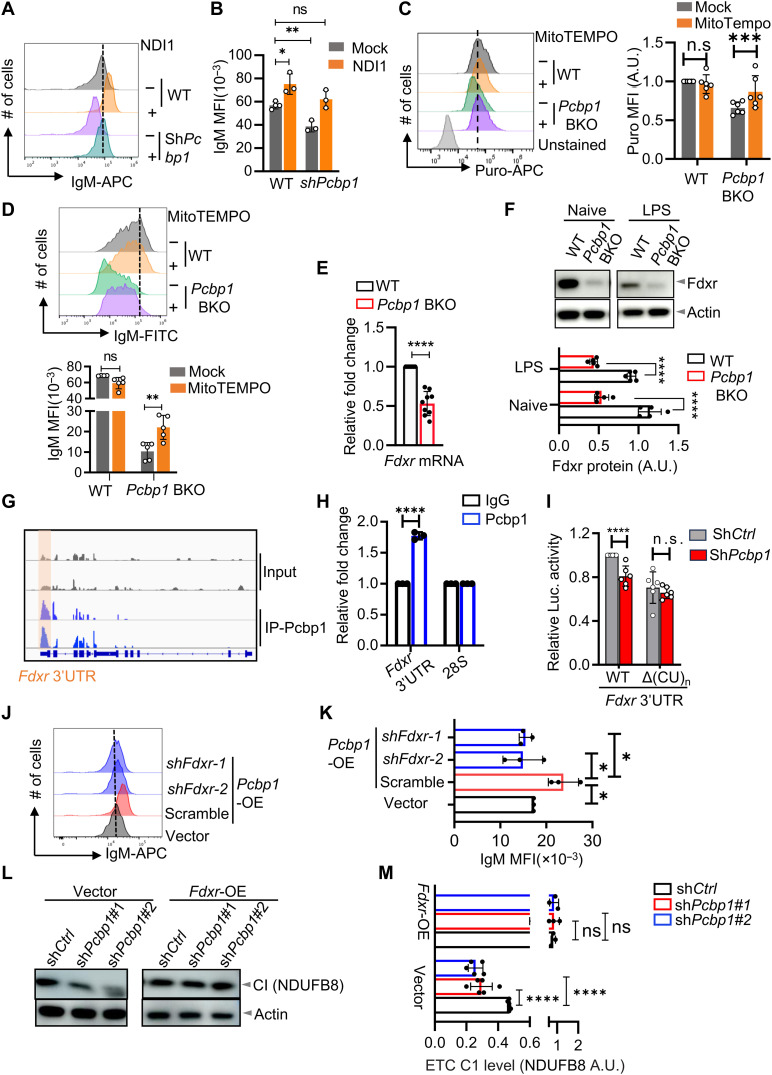
*Pcbp1* enhances IgM expression by maintaining the mitochondrial complex I. (**A** and **B**). *Pcbp1*-deficient Namalwa B cells were transduced with yeast NDI1, and IgM expression was analyzed by flow cytometry. (**C**). MitoTEMPO restores global translation in *Pcbp1* BKO B cells, assessed by Puro incorporation. Left, representative flow plots; right, Puro MFI quantification. ***P < 0.001; two-way analysis of variance (ANOVA) with Sidak’s posttest. A.U., arbitrary units. (**D**). B cells were stimulated with LPS, IL-2, and IL-5 ± MitoTEMPO for 2 days, and IgM expression was measured by flow cytometry. Top, representative plots; bottom, quantification (*n* = 3). (**E**). B cells from indicated mice were treated with LPS, IL-2, and IL-5 for 3 days, and *Fdxr* expression was analyzed by real-time PCR. (**F**). Levels of *Fdxr* protein were determined via immunoblotting. Data are representative (top) and quantitative (bottom) of three independent experiments. (**G** and **H**). WT B cells were stimulated under iGCB condition for 3 days, followed by *Pcbp1* RIP-seq. (G) IGV tracks show *Pcbp1* RIP-seq reads enrichment on *Fdxr* mRNA. (H) Pcbp1 binding to *Fdxr* 3′UTR was confirmed with RIP-qPCR. (**I**). 293T cells were cotransfected with luciferase reporters containing either a WT or ΔCU (*Pcbp1*-binding sites) Fdxr 3′UTR and shPcbp1. Luciferase activity was measured at 48 hours and normalized to renilla. (**J** and **K**). Namalwa B cells were transduced with *Pcbp1* retrovirus and two-independent shRNAs targeting *Fdxr*. IgM expression was assessed by flow cytometry. Representative plots (J) and quantitative (K) of three independent experiments. (**L** and **M**). Pcbp1 knockdown Namalwa cells were rescued with RV-Fdxr, and mitochondrial complex I were analyzed by immunoblotting. (L) Representatives or (M) quantifications of immunoblot images. Data represent three [(A), (B), (D), (F), and (H) to (K)] or six (C) independent experiments. Statistics: Student’s *t* test; *P < 0.05, **P < 0.01, ****P < 0.0001.

To directly test whether mitochondrial rescue could restore IgM expression, we complemented *Pcbp1*-deficient cells with yeast NDI1, a single-subunit complex I homolog (*34*). NDI1 expression increased IgM levels in both WT and *Pcbp1*-deficient cells, with the greater effect in the latter. Notably, NDI1 restored IgM expression in *Pcbp1*-deficient cells to WT levels (Fig. 3, A and B). These results demonstrate that impaired mitochondrial complex I function is a driver of the IgM defect in the absence of Pcbp1.

We next directly targeted mt-ROS production using MitoTEMPO, a mitochondria-targeted antioxidant (*35*). MitoTEMPO treatment reduced mt-ROS levels and restored global protein translation in *Pcbp1*-deficient B cells but had no effect on WT cells (Fig. 3C). Consistently, while MitoTEMPO exerted minimal effects on WT B cells, it markedly increased IgM expression in *Pcbp1*-deficient cells, confirming the role of mt-ROS in suppressing IgM production (Fig. 3D). Consistent with these findings implicating defective protein synthesis, we observed reduced mammalian target of rapamycin complex1 (mTORC1) activity in *Pcbp1*-deficient B cells, as indicated by decreased phosphorylation of ribosomal protein S6 (pS6) (fig. S4, K and L). Given that elevated ROS can impair mTORC1 signaling (*36*), this suggests a potential mechanism linking the mitochondrial defect to translational impairment in the *Pcbp1*-deficient B cells. Collectively, these findings demonstrate that mitochondrial dysfunction, specifically defects in complex I and subsequent ROS generation, underlies the impaired protein translation and IgM expression observed in *Pcbp1*-deficient B cells.

### *Pcbp1* regulates *Fdxr* expression to maintain mitochondrial function and IgM production

*Pcbp1* has dual roles as a nucleic acid–binding protein (*37*) and as an iron chaperone (*14*, *15*). To distinguish these two activities, we used variants of *Pcbp1* that are defective in either RNA binding (ΔRNA) or iron binding capabilities (Δiron), respectively (*38*). Both WT *Pcbp1* and its Δiron variant comparably enhanced IgM expression in Namalwa cells, whereas the ΔRNA mutant showed greatly attenuated capacity (fig. S5, A and B). Similar observation was recapitulated in *Pcbp1*-deficient primary B cells, where WT and Δiron variants, but not the ΔRNA variants of *Pcbp1*, were able to restore IgM expression levels in *Pcbp1*-deficient primary B cells to physiological ranges observed in WT controls (fig. S5, C and D). Thus, *Pcbp1* primarily functions as an RNA binding protein, rather than an iron chaperone, in regulating IgM expression.

To investigate the downstream mechanisms of *Pcbp1*-regulating mitochondrial activity, we concentrated on ferredoxin reductase (*Fdxr*), a crucial component of complex I (*39*). *Fdxr* is indispensable for maintaining mitochondrial integrity, including ATP production, ROS detoxification, and iron homeostasis. Notably, expression of both *Pcbp1* and *Fdxr* was elevated in MzB compared to FoB in WT mice (fig. S5, E and F), suggesting a physiological correlation between these two factors. TMT proteomic analysis revealed a significant reduction of *Fdxr* protein in *Pcbp1*-deficient B cells versus their WT counterparts (Fig. 2D and fig. S5G), which was further confirmed by real-time PCR (Fig. 3E) and immunoblotting (Fig. 3F). Further analysis confirmed that *Pcbp1* deficiency caused pronounced *Fdxr* reduction in Fob cells (fig. S5, H and I). In vitro studies using Namalwa cells demonstrated that *Pcbp1* knockdown decreased *Fdxr* levels (fig. S6A), whereas *Pcbp1* overexpression increased *Fdxr* expression (fig. S6B). However, this effect was largely abolished in the RNA binding–deficient mutant group (fig. S6C). To determine the regulatory mechanism, RNA immunoprecipitation sequencing (RIP-seq) confirmed that *Pcbp1* directly bound to *Fdxr* mRNA in B cells (Fig. 3, G and H), and further examination suggested that *Pcbp1* mainly bound to the last exon and 3′ untranslated region (3′UTR) of *Fdxr* based on previously published enhanced UV crosslinking and immunoprecipitation sequencing (eCLIP-seq) analysis of *Pcbp1* targets in K562 cells (fig. S6D). Knockdown of *Pcbp1* inhibited WT *Fdxr* 3′UTR activity, an effect that was largely abrogated when predicted *Pcbp1*-binding sites were removed from the reporter (Fig. 3I). These data collectively demonstrate that *Pcbp1* was able to promote *Fdxr* expression via modulating its 3′UTR activity.

Knockdown *Fdxr* in Namalwa cells (fig. S6E) phenocopied many aspects of *Pcbp1* deficiency, including reduced rate of protein synthesis (fig. S6F), and diminished IgM expression (fig. S6G). Moreover, the positive effect of *Pcbp1* on IgM expression was abolished by *Fdxr* deletion (Fig. 3, J and K), while *Fdxr* overexpression rescued the reduction in mitochondrial complex I activity and diminished IgM production in *Pcbp1*-deficient B cells (Fig. 3, L and M).

However, the rescue by *Fdxr* was incomplete, suggesting that *Pcbp1* may regulate mitochondrial function through multiple targets. Reanalysis of our RNA sequencing (RNA-seq) data uncovered *Pcbp1*-dependent regulation of *Timm21* splicing (fig. S7A). *Pcbp1* deficiency caused aberrant second intron retention in *Timm21* pre-mRNA (fig. S7, B and C), leading to reduced levels of Timm21 protein (fig. S7, D and E), a component vital for mitochondrial protein import and assembly of complexes I and IV (*40*). Therefore, *Pcbp1* orchestrates B cell mitochondrial homeostasis via multiple posttranscriptional mechanisms, regulating both *Fdxr* expression via its 3′UTR and *Timm21* expression via splicing.

### *Pcbp1* promotes mitochondrial remodeling during B cell activation and GCB cell differentiation

**Fig. 4.**
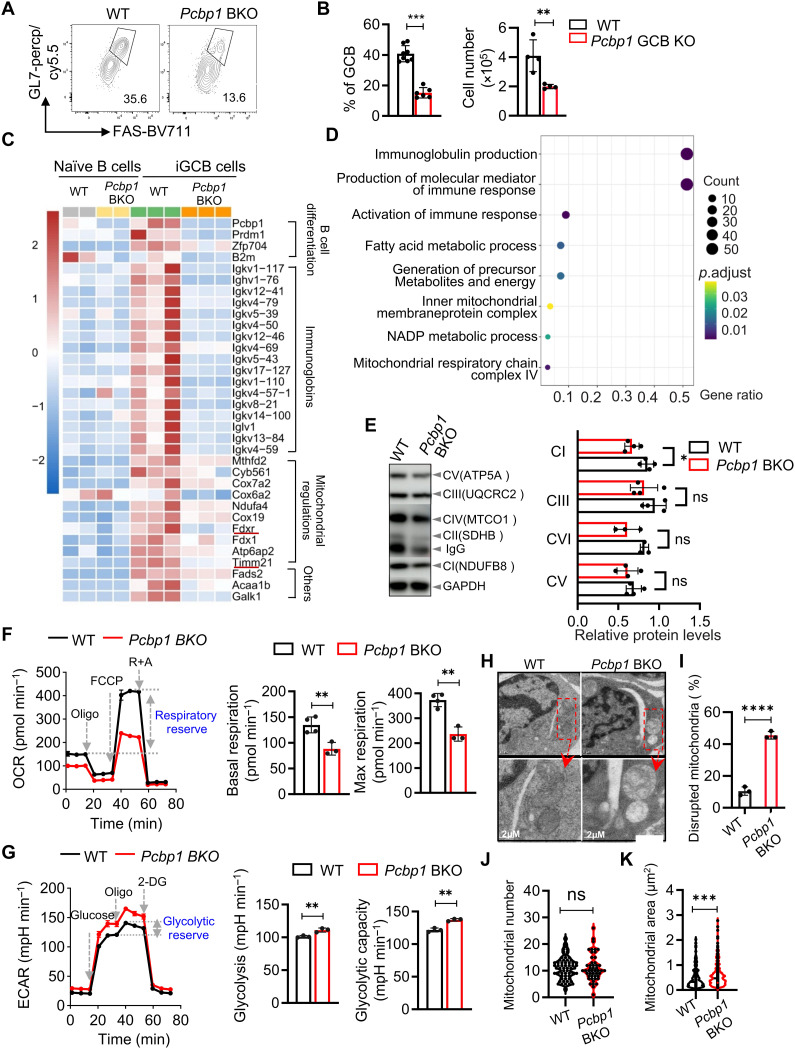
*Pcbp1* promotes differentiation of GCB cells via maintaining mitochondrial activities. (**A** and **B**) WT or *Pcbp1* BKO mice were immunized with NP-KLH. Thirteen days postimmunization, GCB (CD95^+^GL7^+^) were determined in pregated B cells (NP^+^B220^+^CD19^+^) in the spleen. (A), representative FACS plot. (B), quantification of GCB cell frequency and cell number from three independent experiments. (**C**). RNA-seq analysis was performed to identify differentially expressed genes (DEGs) in naïve B cells (CD19^+^B220^+^) and iGCBs (CD95^+^GL7^+^). Heatmaps depict genes that were up-regulated in WT or *Pcbp1*-deficient iGCB cells compared to naïve cells. (**D**). GO term analysis performed on genes that were up-regulated in WT iGCB cells over naïve cells but failed to show similar up-regulation in the *Pcbp1* BKO counterparts. *P* values were calculated on the basis of the cumulative hypergeometric distribution, and *q* values are calculated using the Benjamini-Hochberg procedure. (**E**). Levels of mitochondrial respiratory complexes in iGCB cells were determined by immunoblotting using total OXPHOS antibody cocktail. (Left) representative immunoblot images; (right) quantifications of ETC complex levels from five independent experiments. (**F**) As shown in Fig. 2G, iGCB cells were analyzed using a Seahorse XF analyzer. Data are representative of two independent experiments (*n* = 4 per group). (**G**). As shown in Fig. 2I, ECAR was measured in *Pcbp1*-deficient and WT iGCB cells using a Seahorse XF Analyzer. Representative trace (left) and quantitative analyses of basal/maximum OCR (middle, right) from two independent experiments (*n* = 4 per group). (**H** and **I**). Mitochondrial structures in iGCBs were examined using scanning electron microscopy, and the percentage of mitochondria with disrupted cristae was quantified. Data are representative (H) or the summary (I) of three biological replicates. (**J** and **K**). As in (H), mitochondrial number per cell (J) and mitochondrial sizes (K) were quantified. Data are summarized from three biological replicates. *P < 0.05, **P < 0.01, ***P < 0.001, ****P < 0.0001 in Student’s *t* test.

Given the importance of metabolic remodeling in B cell activation and GCB cell differentiation (*4*, *6*, *27*, *39*, *41*–*45*), we further investigated the function of *Pcbp1* in this process using NP-alum-adsorbed 4-hydroxy-3-nitrophenylacetyl (NP) conjugated to Keyhole limpet hemocyanin (NP-KLH) for immunization. We observed a significant reduction in GCB cells, from 35.6 to 13.6% following NP-KLH immunization (Fig. 4, A and B). The total number of GCB cells was also markedly reduced (Fig. 4B). In addition, in an in vitro culture system (*46*), naïve B cells from WT or *Pcbp1* BKO mice were differentiated into GCB cells (iGCB) in the presence of interleukin-4 (IL-4) and CD40L/BAFF-expressing stromal cells. *Pcbp1*-KO B cells displayed diminished differentiation efficiencies into GCB cells, as determined by GL7 and Fas staining (fig. S8A). The reduction in GL7 expression is unlikely to reflect a general defect in B cell activation (fig. S8B) because other activation-induced genes (e.g., CD80, CD86, and CD69) were not down-regulated in *Pcbp1*-deficient iGCB cells compared to the WT cells (fig. S8C). These data indicate that *Pcbp1* is essential for differentiating naïve B cells into GCB cells.

We next used an unbiased and high-throughput RNA-seq approach to determine molecular pathways affected by *Pcbp1* during iGCB differentiation. As previously reported, the differentiation process of naïve B cells into iGCB cells was accompanied by an up-regulation of mitochondria-related genes (cluster 2) and Ig genes (cluster 3) (*4*, *6*, *27*, *39*, *41*, *47*). Notably, such up-regulation was completely abolished by *Pcbp1* deficiency (Fig. 4C). GO analysis further confirmed that the down-regulated genes in *Pcbp1*-deficient iGCB cells were enriched for antibody production and mitochondrial/metabolism (Fig. 4D). The latter includes genes involved in fatty acid metabolic process, inner mitochondrial membrane protein complex, and mitochondrial respiratory chain complex IV. Immunoblotting also revealed that complex I and IV protein levels were decreased in *Pcbp1-*deficient iGCBs (Fig. 4E). These findings highlight *Pcbp1*’s essential role in orchestrating metabolic shifts that are fundamental for successful GCB cell differentiation and function.

Consistent with these transcriptional changes, *Pcbp1*-deficient iGCBs displayed mitochondrial dysfunction similar to that in *Pcbp1*-deficient naïve B cells. Seahorse metabolic flux analysis demonstrated diminished OXPHOS (as determined by OCR) (Fig. 4F) concomitant with enhanced glycolytic compensation (as determined by ECAR) in *Pcbp1*-deficient iGCB cells compared to WT controls (Fig. 4G). Transmission electron microscopy (TEM) showed that more mitochondria had disrupted morphology with disorganized cristae [40% in knockout (KO) versus 10% in WT] (Fig. 4, H and I) in *Pcbp1*-deficient iGCB cells. Although numbers of mitochondria were comparable, *Pcbp1*-deficient iGCB cells had more swollen mitochondria than WT iGCB cells (Fig. 4, J and K). Similar to naïve B cells, *Pcbp1*-deficient iGCB cells also exhibited elevated mt-ROS levels compared to WT iGCB cells (fig. S8D). Consequently, under the iGCB conditions, *Pcbp*1-deficient cells showed compromised proliferation and expansion based on carboxyfluorescein diacetate succinimidyl ester (CFSE) dilution assays (fig. S8E). The mitochondria-targeted antioxidant MitoTEMPO rescued iGCB differentiation (fig. S8, F and G), indicating a causal role for mitochondrial dysfunction in the observed defects in GCB differentiation in vitro.

### *Pcbp1* enhances GC responses in vivo

Building on the findings from *Pcbp1* BKO mice, we aimed to selectively abrogate *Pcbp1* in GCB cells while bypassing its deficiency in naïve B cells. To achieve this, we bred *Pcbp1^fl/fl^* mice with *AID-Cre* mice, where Cre recombinase is specifically expressed in GCB cells (*48*). Subsequently, we immunized the *Pcbp1*^fl/fl^ *AID*-Cre mice (referred to as *Pcbp1* GCB KO thereafter) with NP-KLH. The partial deletion of *Pcbp1* in AID-Cre *Pcbp1*^fl/fl^ GCB cells was confirmed through intracellular staining at day 13 postimmunization (fig. S9A).

**Fig. 5.**
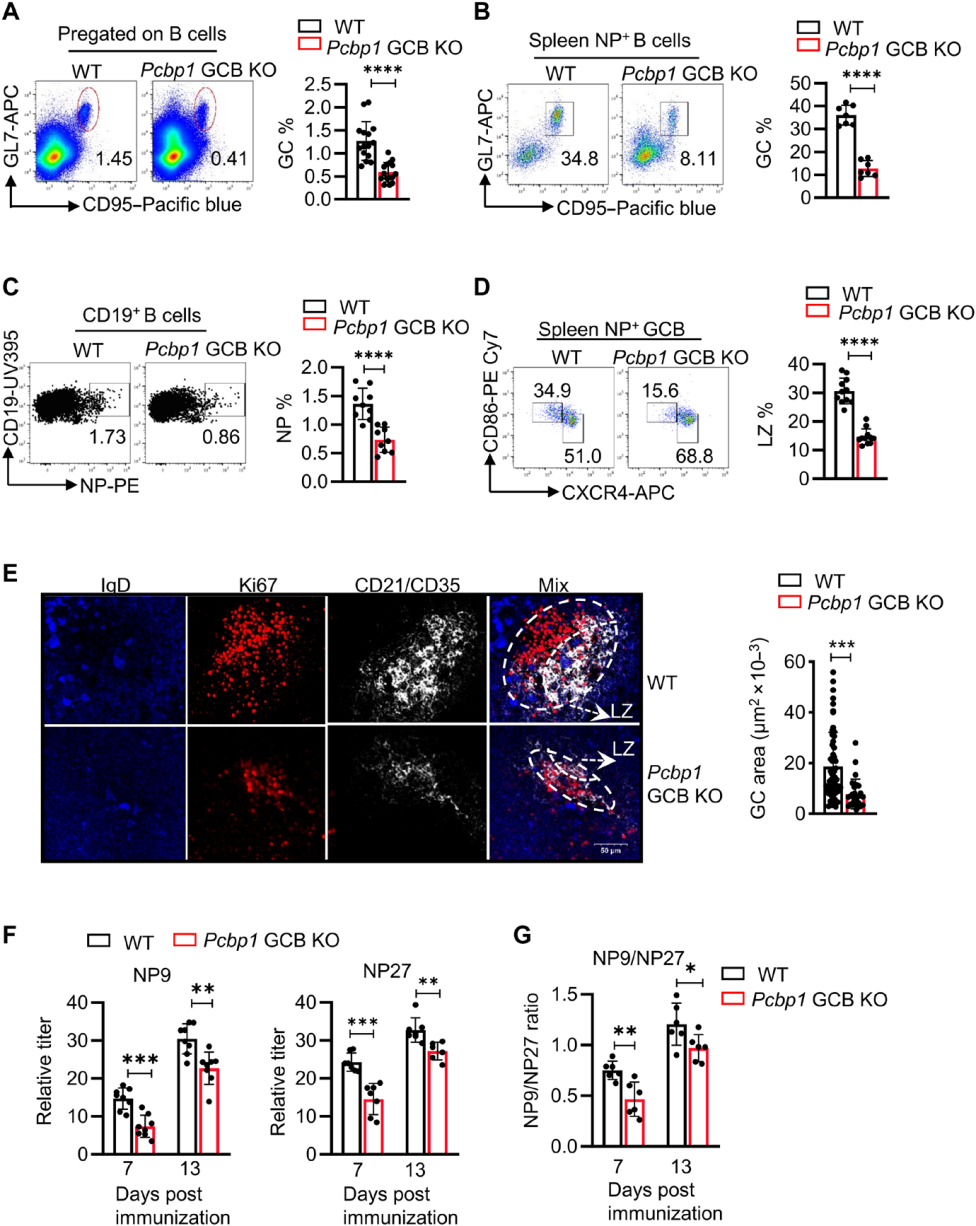
*Pcbp1* deficiency in GCB cells results in impaired GC responses. *AID ^Cre/+^Pcbp1^fl/fl^* (*Pcbp1* GCB KO) and *Pcbp1^fl/fl^* mice (6 to 8 weeks) were immunized with NP-KLH and Alum. (**A**). Thirteen days postimmunization, GCB (CD95^+^GL7^+^) cells were determined in pregated B cells (B220^+^CD19^+^) in the spleen. Left, representative flow cytometric analysis; right, summary of percentages of GCB cells within total B cells from three independent experiments. (**B**). As in (A), NP-specific GCB cells were determined in the pregated NP^+^ B cells (B220^+^CD19^+^ NP^+^) in the spleen. Left, representative flow cytometric analysis; right, summary of percentages of GCB cells within NP-specific B cells from three independent experiments. (**C**). As in (A), NP-specific B cells in the spleen were determined. Data are representative (left) or summary of percentages of NP-specific B cells within total B cells (right) from three independent experiments (*n* = 9 mice). (**D**). As in (B), LZ and DZ B cells were determined by CXCR4 and CD86 staining in the pregated NP-specific GCB cells. Left, representative staining; right, summary of LZ B cells percentage from three independent experiments (*n* = 10 mice). (**E**). As in (A), sizes of GC in the spleen were analyzed by immunofluorescence staining of IgD, Ki67, and CD21/CD35. Left, representative results; right, summary of GC size from three independent experiments, and each dot represents one GC. (**F** and **G**). High-affinity anti-NP antibodies (NP9) and low affinity anti-NP antibodies (NP27) in the serum were determined by ELISA at day 7 and day 13 after the NP-KLH immunization (F). Affinity maturation was determined by calculating NP9/NP27 ratios (G). Data from three independent experiments, and each dot represents one mouse. Statistical significance was determined using Student’s *t* test: *P < 0.05, **P < 0.01, ***P < 0.001, ****P < 0.0001.

Analysis at day 13 postimmunization revealed that *Pcbp1* GCB KO mice exhibited dramatic reductions in both total (Fig. 5A and fig. S9B) and NP-specific GCB cells (Fig. 5B), while the ratio of naïve B cells remained unaltered (fig. S9C). NP-specific B cells were also diminished in the *Pcbp1* GCB KO mice (Fig. 5C). Within the NP-positive GCB-cell population, a significant reduction of LZ B cells compared to dark zone (DZ) B cells was observed (Fig. 5D). The reduction in GC and LZ sizes was also corroborated by immunofluorescence staining on splenic sections from the immunized *Pcbp1* GCB KO mice (Fig. 5E). Unlike in vitro activation, the impaired GC reaction occurred without detectable alterations in GCB cell apoptosis (fig. S9D) or proliferation (fig. S9E) in *Pcbp1*-deficient GCB cells. This finding suggests a role for *Pcbp1* in regulating GCB differentiation or maintenance pathways rather than primarily controlling cell turnover. Similarly, sheep red blood cells (SRBCs) immunization showed GCB deficiency (fig. S9F) and LZ reduction in *Pcbp1* GCB KO mice (fig. S9G).

To further evaluate the outcome of GC responses, we assessed the quantity and avidity of serum antibodies targeting NP using enzyme-linked immunosorbent assay (ELISA). At 7 and 13 days postimmunization, *Pcbp1* GCB KO mice exhibited nearly equivalent levels of NP-specific IgM antibodies compared to *AID*-Cre control mice (fig. S9H). Conversely, these mice displayed significantly lower levels of circulating NP-specific IgG1 in the peripheral blood compared to control mice (Fig. 5F). Furthermore, the ratios of high-affinity NP-specific IgG1 (NP9) to low-affinity NP-specific IgG1 (NP27) were reduced, indicating impaired antibody affinity maturation in *Pcbp1* GCB KO mice (Fig. 5G). These findings were further supported by a similar reduction of antigen-specific IgG following SRBC immunization (fig. S9I). Although frequencies of total plasma cells (CD138^+^TACI^+^) in the spleen were not changed following NP-KLH immunization (fig. S9J), bone marrow NP-specific antibody-secreting cells (ASCs) were reduced in the *Pcbp1* GCB KO mice at day 13 postimmunization, determined by enzyme-linked immunospot (ELISPOT) analysis (fig. S9, K and L). Together, these data indicate that although extrafollicular differentiation may generate comparable CD138^+^ plasma/plasmablast cells, robust generation of antigen-specific long-lived plasma cells requires *Pcbp1* activity in GCB cells.

Mechanistic analysis revealed that *Pcbp1*-deficient GCB cells exhibited diminished mitochondrial mass (MitoTracker Deep Red) and impaired ETC activity (COXI-AF488, cytochrome c) compared to controls (fig. S10, A and B). Despite the reduced OXPHOS, ATP levels were slightly elevated in *Pcbp1*-deficient GCB cells (fig. S10C), likely due to compensatory mechanisms (e.g., elevated glycolysis). However, the limited number of GCB cells from *Pcbp1*-deficient mice prevented direct assessment of mitochondrial function by Seahorse analysis.

Beyond metabolic dysfunction, protein translation was compromised in the *Pcbp1*-deficient GCB cells as determined by Puro incorporation assay (fig. S10D). Unlike IgM expression in the naïve B cells, however, IgG1 levels were comparable between *Pcbp1*-deficient and WT GCB cells (fig. S10, E and F), likely because robust antibody expression is essential for positive selection during GC responses, leading to counter-selection against low antibody-producing cells.

To further validate the cell-autonomous role of *Pcbp1*, we leveraged the incomplete Cre-mediated depletion in the *AID*-Cre mice to compare *Pcbp1*^high^ and *Pcbp1*^low^ cells within the same *Pcbp1* GCB KO mice (fig. S10G). Only the *Pcbp1*^low^, but not *Pcbp1*^high^ GCB cells exhibited reduced mitochondrial mass (fig. S10H), ETC activity (fig. S10, I and J), and protein translation (fig. S10K). These data further underscored the importance of *Pcbp1* as a cell-autonomous factor for maintaining mitochondrial integrity in the GCB cells.

### scRNA-seq analysis of *Pcbp1*-deficient GCB cells

**Fig. 6.**
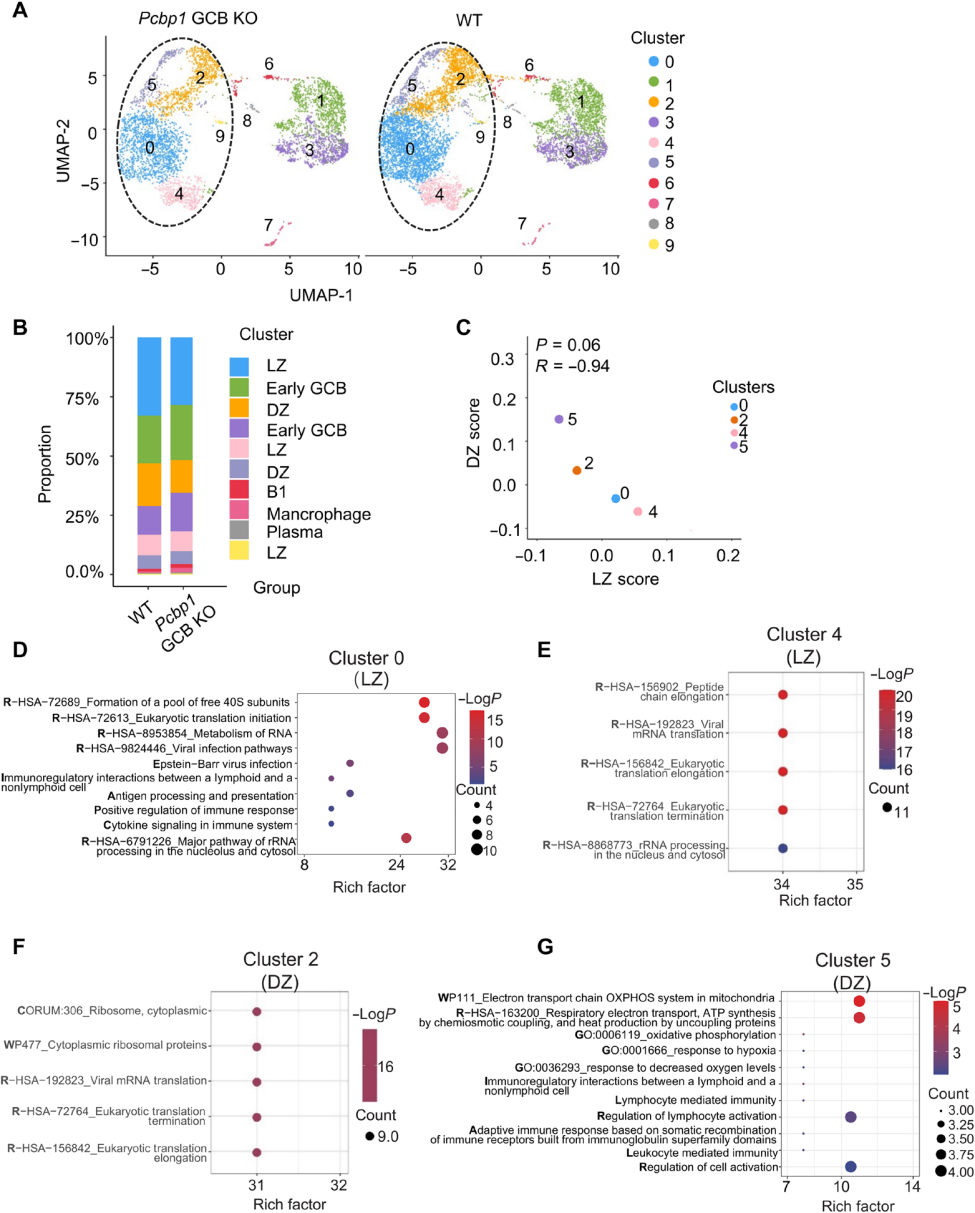
scRNA-seq analysis of *Pcbp1*-deficient GCB cells. scRNA-seq analysis of NP^+^ GC B cells from WT and *AID^Cre/+^Pcbp1^fl/fl^* (*Pcpb1* GCB KO) mice. Thirteen days after NP-KLH immunization. NP^+^ GC B cells were sorted by flow cytometry (B220^+^CD19^+^ NP^+^ CD95^+^ GL7^+^) and analyzed by scRNA-seq. (**A**). The Uniform Manifold Approximation and Projection (UMAP) visualization of the scRNA-seq results. Each point corresponds to an individual cell (WT = 7464, *Pcbp1* GCB KO = 4951). Clusters 0, 2, 4, 5, and 9 represented GC B cells. (**B**). Proportions of the 10 identified clusters in WT and *Pcpb1* GCB KO mice. (**C**). Shown is the average expression level of DZ/LZ signature scores of GC B cells in different clusters. The Pearson correlation coefficient (*R*) and the *P* values are indicated. (**D** to **G**). GO analysis of DEGs in GCB clusters as shown in (A). *P* values are calculated on the basis of the cumulative hypergeometric distribution, and *q* values are calculated using the Benjamini-Hochberg procedure.

To gain further insight into *Pcbp1*-mediated transcriptional programs in GC response in vivo, we performed single-cell RNA sequencing (scRNA-seq) of NP-specific GCB cells (B220^+^Fas^hi^GL7^hi^NP-PE^+^). We obtained 7464 and 4951 high-quality GCB cells passing initial filtering from WT and *Pcbp1* GCB KO mice, respectively. Ten clusters were annotated by differentially expressed genes (DEGs) (Fig. 6A and fig. S11A), including GCB, early GCB, B1, plasma B cells and macrophages (Fig. 6, A and B). Clusters 1 and 3 were classified as early GCB cells, characterized by up-regulation of B cell activation markers but lacking robust *Aicda* (AID) expression (fig. S11A). These clusters retained *Pcbp1* expression in *Pcbp1* GCB KO mice, suggesting that they represent a pre-GC or transitional population that had not yet undergone Cre-mediated recombination (fig. S11B).

Clusters 0, 2, 4, and 5 were identified as GC B cells that exhibited efficient *Pcbp1* depletion in *Pcbp1* GCB KO mice and were therefore the focus of subsequent analyses (fig. S11B). Within GCB cells, clusters 2 and 5 showed high expression of DZ genes and low expression of LZ genes (Fig. 6C). In contrast, clusters 0 and 4 displayed a reciprocal expression pattern, indicating distinct spatial and functional stages within the GC (Fig. 6C).

We observed that *Pcbp1* expression was up-regulated in the DZ of GCs (fig. S11C). Pathway enrichment analysis revealed that DZ B cells preferentially elevated OXPHOS compared to LZ B cells (fig. S11, D to F), resulting in elevated mt-ROS levels in DZ clusters (fig. S11G). These results suggest that *Pcbp1* may play a pivotal role in coordinating metabolic programs that support GCB cell function. GO analysis of DEGs between *Pcbp1*-deficient and control cells in these clusters highlighted significant enrichment in pathways related to eukaryotic translation and RNA metabolism in the LZ and DZ (Fig. 6, D to F), as well as in the ETC and OXPHOS pathways (Fig. 6G). This suggests that *Pcbp1* plays a critical role in mitochondrial remodeling and metabolic processes crucial for GC B cell differentiation and function.

### *Pcbp1* enhances GC response partially through *Fdxr* up-regulation and mt-ROS suppression

**Fig. 7.**
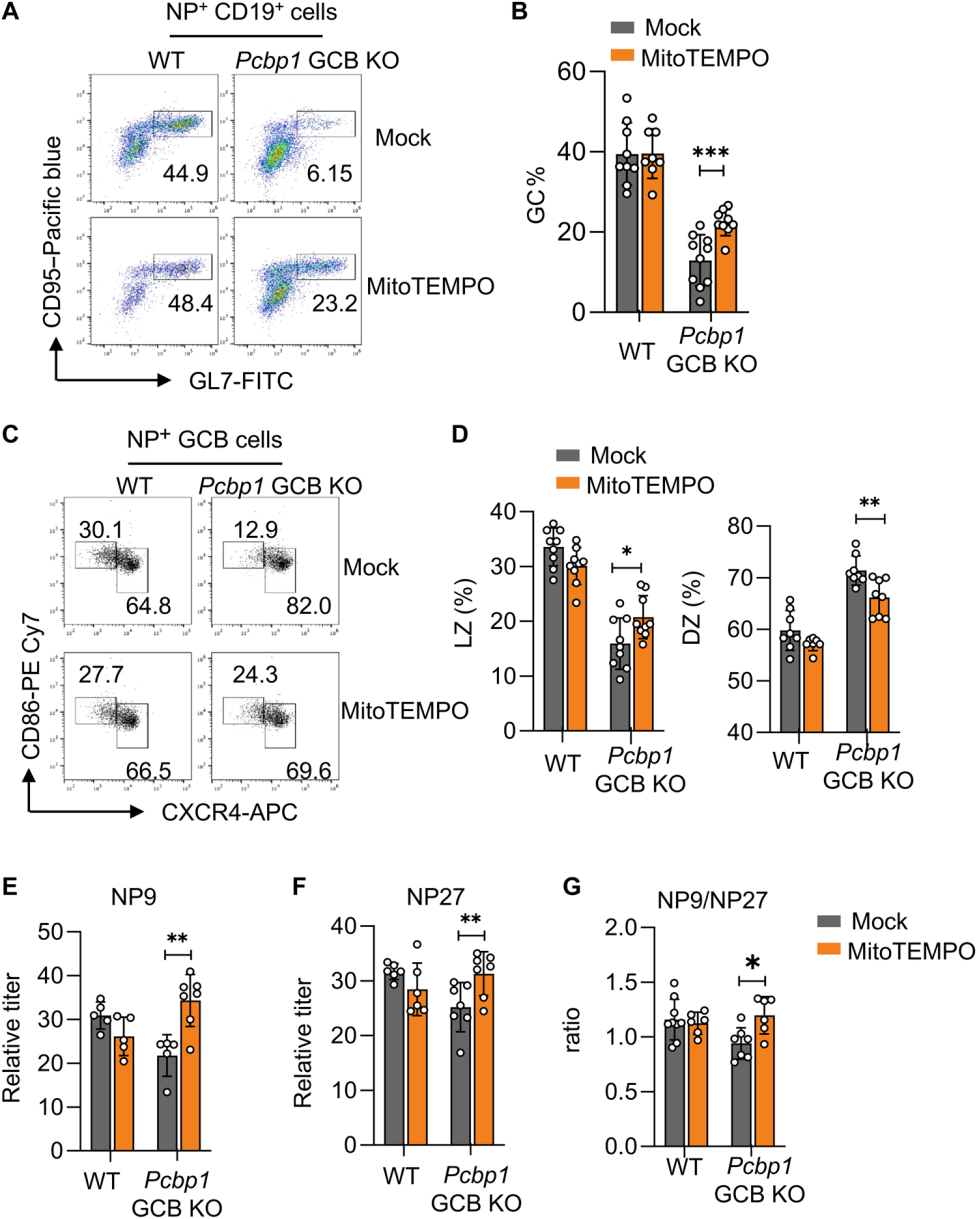
MitoTempo rescues GC defects associated with loss of *Pcbp1*. Eight-week-old WT or *Pcbp1* GCB KO (*AID^Cre/+^Pcbp1^fl/fl^*) mice were treated with MitoTEMPO (5 mg/kg) via daily intraperitoneal injection from day −7 to day 13. The mice were immunized with NP-KLH on day 0. (**A** and **B**). MitoTEMPO partially restored differentiation of GCB cells in the *Pcbp1*-deficient mice. Thirteen days after the NP-KLH immunization, B cells expressing NP-specific antibodies in the spleen were identified as CD19^+^NP^+^, and percentages of GCB in NP-specific cells were subsequently identified on the basis of GL7 and CD95 staining. (A). Representative flow cytometric analysis; (B) summary of percentages of GCB cells within NP^+^ B cells from three independent experiments. ***P < 0.001 in Student’s *t* test. (**C** and **D**). MitoTEMPO partially rescued LZ B cells in *Pcbp1*-deficient GCB cells. As in (A), LZ/DZ ratio in GCB cells were determined by CXCR4 and CD86 staining. (C). Representative staining. (D). Summary of three independent experiments with total of nine mice. **P < 0.01 in Student’s *t* test. (**E** to **G**). MitoTEMPO promoted affinity maturation in *Pcbp1*-deficient mice. As in (A), the high-affinity anti-NP antibodies (NP9) and low affinity anti-NP antibodies (NP27) in the serum were determined by ELISA at day 7 and day 13 after the NP-KLH immunization (E and F). Affinity maturation was determined by comparing ratios of NP9 /NP27 (G). Data from three independent experiments. Statistical significance was determined using Student’s *t* test: *P < 0.05, **P < 0.01.

Last, we examined whether *Pcbp1* promoted GC response in vivo via up-regulating *Fdxr* and detoxification of mitochondria. Similar to the IgM expression, MitoTEMPO rectified the defective GC responses in *Pcbp1* GCB KO mice following the NP-KLH immunization. Thirteen days following the immunization, MitoTEMPO did not affect the differentiation of GCB cells in the spleen of WT mice but significantly increased GCB cell percentage in *Pcbp1* GCB KO mice (from 6 to 23%) (Fig. 7, A and B). Within GCB cells of *Pcbp1* GCB KO mice, the proportion of LZ B cells also increased after MitoTEMPO treatment (from 12.9 to 24.3%), while the fraction of DZ B cells decreased (from 82.0 to 69.6%) (Fig. 7, C and D). Consistent with the enhanced GC response, MitoTEMPO treatment also increased the production of high-affinity NP-specific antibodies (as measured by ratios of NP9/27-specific IgG) in the *Pcbp1* GCB KO mice (Fig. 7, E to G).

Furthermore, restoration of *Fdxr* in *Pcbp1*-deficient B cells partially rescued GCB cell defects. As shown in fig. S12A, we reconstituted μMT mice (endogenous B cell deficient) with *Pcbp1* BKO bone marrow cells transduced with retrovirus vectors expressing either *Fdxr*–internal ribosomal entry site–green fluorescent protein (GFP) (RV-*Fdxr*) or a control empty vector. Six weeks after reconstitution, *Fdxr* expression partially rescued defective IgM expression in *Pcbp1*-deficient naïve B cells. Splenic naïve B cells from mice receiving *Fdxr*-expressing bone marrow exhibited higher level of IgM compared to empty vector controls (fig. S12B).

Although the reconstitution efficiencies were relatively low (fig. S12C), potentially leading to suboptimal GC induction, we proceeded to immunize the chimeric mice with NP-KLH to evaluate GC responses. Thirteen days postimmunization, GFP^+^ B cells in RV-*Fdxr*–transduced groups showed a moderate increase of GCB cell frequencies (fig. S12, D and E) and elevated LZ B cell proportions (fig. S12, F and G) within NP^+^ GCB cells. The mitochondria mass was also elevated in *Fdxr*-expressing GCB cells (fig. S12, H and I). Although further optimization of the reconstitution system will be necessary to rigorously determine whether Fdxr restoration can completely rescue GCB responses in *Pcbp1*-deficient mice, these findings suggest that *Pcbp1* promotes GCB cell differentiation, at least partially through enhancing *Fdxr* expression and attenuating mt-ROS accumulation.

## DISCUSSION

Our study establishes *Pcbp1* as a critical regulator of mitochondrial integrity in B cells, essential for effective antibody production and robust GC responses. Through its RNA binding activity, *Pcbp1* regulates the expression of *Fdxr* to modulate production of mt-ROS, thereby maintaining mitochondrial function. These findings uncover a connection between posttranscriptional regulation, mitochondrial dynamics, and humoral immunity, providing insights into the mechanisms that govern antibody production and GC formation.

Mechanistically, *Pcbp1* regulates mitochondrial function mainly through its interaction with *Fdxr* mRNA, a critical regulator of complex I activities and FeS cluster biogenesis. The binding of *Pcbp1* to the 3′UTR of *Fdxr* mRNA enhances its stability and expression, thereby supporting mitochondrial integrity and function. This is particularly important in the context of B cells, where efficient mitochondrial function is essential for sustaining the energy demands of protein synthesis, especially during the rapid expansion and differentiation phases of GC responses. The partial rescue achieved by *Fdxr* overexpression indicates that *Pcbp1* likely regulates mitochondrial function through multiple targets, including *TIMM21* splicing regulation—a component essential for mitochondrial protein import and assembly of respiratory complexes I and IV. Notably, *Fdxr* is not only a direct complex I subunit but, together with FDX2, contributes to FeS cluster biogenesis, thereby influencing the activity of multiple FeS-containing enzymes, including—but not limited to—complex I. Thus, mitochondrial dysfunction in *Pcbp1*-deficient cells likely reflects impaired activity across several FeS-dependent enzymes, with complex I representing one critical, although not exclusive, target.

The restoration of IgM expression following the rescue of mitochondrial function with NDI1 or MitoTEMPO treatment further supports the causal link between mitochondrial dysfunction and impaired IgM production in *Pcbp1*-deficient cells. Our RNA-seq analysis further revealed that *Pcbp1* orchestrates broader mitochondrial and metabolic pathway regulation during GC differentiation, with down-regulation of mitochondrial gene expression in *Pcbp1*-deficient cells highlighting its role in metabolic remodeling necessary for effective GC responses.

The impact of *Pcbp1* deficiency varies across different B cell developmental stages, reflecting the distinct metabolic and mitochondrial requirements at each stage. While naïve B cells use the ETC to generate ATP, GCB cells exhibit high proliferation and metabolic activity rates upon activation. *Pcbp1* deficiency–induced mitochondrial defects may impair IgM production in naïve B cells without significantly disrupting their homeostasis. In contrast, these defects critically undermine the survival and expansion of GCB cells. Further research is warranted to determine whether ETC defects contribute to the pro-B to pre-B cell transition defects observed in the bone marrow.

*Pcbp1* exerts distinct regulatory effects among Ig isotypes. The most pronounced impact occurs on IgM, where *Pcbp1* deficiency significantly reduces both surface and cellular IgM levels across multiple B cell subsets, including follicular, marginal zone, and B1a cells, extending to reduced serum IgM levels. This regulatory role persists in activated B cells following LPS stimulation. In contrast, IgD levels remain largely unaltered, although MzB cells display elevated IgD expression. For IgG isotypes, the effects are more nuanced, suggesting selective and context-dependent regulation of different IgG subtypes and their induction pathways.

mt-ROS are recognized as key mediators of cellular signaling in various contexts (*49*–*54*). GCB cells also require tightly regulated ROS dynamics to balance survival, proliferation, and apoptosis during affinity maturation (*1*, *10*). These cells exhibit elevated mitochondrial activity compared to naïve B cells, creating a prooxidative environment particularly pronounced in proliferating DZ cells, as confirmed by our scRNA-seq analysis. *Pcbp1* deficiency disrupts this delicate balance, leading to elevated mt-ROS that impairs GC responses. By maintaining mitochondrial integrity through *Fdxr* and *TIMM21* regulation, *Pcbp1* prevents excessive ROS accumulation in this metabolically demanding environment. The resulting protein synthesis defects in *Pcbp1*-deficient GCB cells likely reflect both ROS-induced damage and direct mitochondrial dysfunction, highlighting the critical intersection of ROS control, metabolic reprogramming, and mitochondrial quality control in shaping GC fate decisions.

The elevated ROS generation following *Pcbp1* loss likely originates from multiple sources, including complexes I and III (*55*, *56*). Under conditions of highly reduced Coenzyme Q (CoQ) pools and elevated mitochondrial membrane potential, complex I can generate substantial ROS through reverse electron transport—a mechanism likely contributing significantly to amplified ROS generation in *Pcbp1*-deficient cells. Similar mechanisms may explain reported mitochondrial defects in *Pcbp1*-deficient hepatocytes.

A notable consequence of *Pcbp1* deficiency–induced mitochondrial dysfunction is the suppression of cytosolic translation in B cells. Previous studies have demonstrated that ROS can inhibit protein translation by altering the redox status of key synthesis proteins (*57*) and reducing newly synthesized protein burden. Ribosomal proteins may function as oxidative stress sensors, modulating translation by halting postinitiation processes. Elevated ROS levels can decrease ribosomal pS6 phosphorylation (*36*), potentially through mTOR pathway modulation or direct translation machinery impacts. Investigating whether similar mechanisms underlie ROS effects on cytosolic translation in B cells represents an important future research direction.

Notably, the *Pcbp1* deficiency only resulted in the diminished proliferation upon in vitro activation but not in GCB cells in vivo. This discrepancy may reflect the selective nature of the GC response, where B cells with proliferative or survival defects are outcompeted and eliminated during selection. Consequently, in vivo assays such as 5-bromo-2′-deoxyuridine incorporation or caspase-3 staining likely capture only the surviving, successfully selected cells, masking early defects. In contrast, the in vitro system, which lacks this competitive pressure, unmasks the intrinsic proliferative and survival disadvantage caused by *Pcbp1* loss.

Regarding the specificity of translational effects on IgM versus IgD, we believe that this reflects a case of selective translational reprogramming, a well-characterized cellular stress response. Specifically, complex I–derived ROS and mTOR inhibition are known to trigger such reprogramming, suggesting an active and regulated reallocation of translational resources rather than a passive and complete failure of the translational machinery. Several mechanisms may underlie this specificity. For example, differences in the 3′UTRs between μ mRNA and δ mRNA could confer differential sensitivity to stress conditions. In addition, μ mRNA is more abundant than δ mRNA, and the biosynthesis of pentameric IgM imposes a greater demand on chaperone capacity compared to the production of membrane-bound IgD. In conclusion, we propose that IgM translation is selectively impaired under stress conditions due to transcript-specific features of μ mRNA. This impairment is consistent with stress-induced translational reprogramming and reflects molecular determinants intrinsic to the IgM transcript rather than a generalized dysfunction of the cellular translation machinery. Together, these findings support a model in which *Pcbp1* deficiency modestly impairs global protein synthesis via mt-ROS but exerts a specific regulatory effect on IgM production.

Given the established role of mitochondrial dysfunction in various immune disorders, including autoimmune diseases and immunodeficiencies, our findings suggest potential therapeutic avenues targeting mitochondrial pathways to modulate immune responses. The selective effects of *Pcbp1* on different B cell stages and antibody isotypes may pave the way for therapeutic strategies aimed at enhancing protective immunity while minimizing autoimmune responses.

## MATERIALS AND METHODS

### Mice

*Pcbp1*^fl/fl^ mice were provided by C. C. Philpott (National Institute of Diabetes and Digestive and Kidney Diseases) and backcrossed to C57BL/6 background for over 10 generations. Mb1-Cre, AID-Cre, and μMT mice were obtained from the Jackson laboratory, and all Cre-expressing lines were maintained in the heterozygous state throughout this study. All mice were maintained under specific pathogen–free conditions. All animal studies were performed in compliance with the guide for the care and use of laboratory animals and were approved by the institutional biomedical research ethics committee of Westlake University (AP#24-066-CX-12).

### Cell lines

Human embryonic kidney 293T cells and Namalwa B cells were obtained from American Type Culture Collection and cultured in Dulbecco’s modified Eagle’s medium (DMEM, Hyclone, USA) or RPMI 1640 (Hyclone, USA) with 10% fetal bovine serum (FBS, Hyclone, USA), respectively. 40LB-IL4 cell lines were provided by C. Xiao and cultured in DMEM with 10% FBS. For iGCB culture, B cell medium [RPMI 1640 with 10% FBS, 10 mM Hepes-NaOH (pH 7.5), minimum essential medium nonessential amino acids solution, 50 μM β -mercaptoethanol, and gentamycin (50 mg/ml)] was used instead of DMEM.

### Lymphocyte staining and flow cytometry

For cell surface staining, cells were washed with fluorescence-activated cell sorting (FACS) buffer (2% FBS in phosphate-buffered saline (PBS) with 1 mM EDTA), blocked with an antibody against CD16/32 (2.4G2, BD Pharmingen), and incubated with indicated surface antibodies on ice for 30 min. The cells were then washed two more times with FACS buffer and analyzed with a CytoFLEX machine (Beckman). For intracellular staining, cells were fixed/permeabilized with a Cytofix/Cytoperm kit (BD Bioscience) and stained with antibodies against indicated proteins. To determine expression of *Pcbp1*, the cells were first cytofixed and permeabilized (BD Bioscience), stained with rabbit anti-*Pcbp1* antibody (1:500, MBL, RN0249P) for 1 hour, and followed by Alexa Fluor 647 donkey anti-rabbit IgG (H + L) (1:1000, Invitrogen, A31573).

The following FACS antibodies were used in the study: IgM–fluorescein isothiocyanate (FITC, 1:200, II/41, BD Biosciences, 553437), IgD–Pacific blue (1:200, 11-26c.2a, BioLegend, 405712), CD95-BV421 (1:200, Jo2, BD Biosciences, 562633), GL7–allophycocyanin (APC) (1:200, GL7, BioLegend, 144618), NP–phycoerythrin (PE) (1:200, Biosearch, N-5070-1), CD19-UV395 (1:200,1D3, BD Biosciences, 563557), CXCR4-APC (1:200,2B11, Invitrogen, 17-9991-82), CD86-PE/Cy7 (1:200, GL1, BD Biosciences, 560582), GL7-FITC (1:200, GL7, BioLegend, 144604), GL7-PerCP/Cy5.5 (1:200, GL7, BioLegend, 144610), CD19-APC (1:200, 6D5, BioLegend, 115512), B220-PE(1:200, RA3-6B2, BioLegend, 103208), CD19- PE/Cy7 (1:200, 6D5, BioLegend, 115520), CD93-PE (1:200, AA4.1, BioLegend, 136503), c-Kit–PE(1:200, 2B8, Invitrogen, 12-1171-82), CD25-APC(1:200, PC61, BioLegend, 102012), IgM-PE/Cy7(1:200, II/41, Invitrogen, 25-5790-82), B220-PerCP/Cy5.5 (1:200, RA3-6B2, BD Biosciences, 552771), CD95-PE(1:200, 15A7, Invitrogen, 12–0951-81), anti-human IgM-APC (1:200, HIB19, BioLegend, 302212), and anti-human CD19-PE (1:200, HIB19, BioLegend, 302254).

### Measurement of mt-ROS

To detect mt-ROS, cells were incubated with MitoSOX Red superoxide indicator (Invitrogen) at a final concentration of 5 μM for 30 min at 37°C, according to the manufacturer’s instructions. Following this, the cells were labeled with cell surface antibodies as described previously.

For intracellular total ROS level, splenic cells were stained with 10 μM carboxy-H2DCFDA at 37°C for 30 min. Total ROS levels were measured via flow cytometry.

### RNA extraction and real-time quantitative PCR

Total RNA was isolated with RNeasy plus kit (Qiagen, CA), and cDNA was synthesized using PrimeScript RT reagent kit with genomic DNA (gDNA) eraser (TaKara, Japan). Quantitative PCR was performed using SYBR Premix Ex Taq (TaKara, Japan) on a StepOnePlus real-time PCR system (Life Tech, USA). mRNA abundance was quantified by calculating the C_T_ of the target of interest and normalizing to actin or glyceraldehyde-3-phosphate dehydrogenase (Gapdh) (Δ C_T_). Primer sequences were listed in table S2.

### SDS-PAGE and immunoblotting

Cells were lysed in radioimmunoprecipitation assay (RIPA) lysis buffer (EMD Millipore) with 0.25% SDS, 1.0 mM dithiothreitol DTT (Sigma-Aldrich) and protease inhibitor cocktail (Roche Diagnostics). Samples were centrifuged at 4°C, 10,000*g* for 15 min. Lysates were denatured at 50°C for 10 min before SDS–polyacrylamide gel electrophoresis (SDS-PAGE). Gels were transferred to polyvinylidene difluoride membranes. Membranes were blocked in 5% milk for 1 hour at room temperature before overnight incubation at 4°C with primary antibodies. Membranes were washed in PBS and 0.1% Tween and incubated with horseradish peroxidase (HRP)–conjugated secondary antibodies for 1 hour before imaging using a ChemiDoc MP Imaging System (Bio-Rad).

The following antibodies were used in Western blotting: goat anti-mouse IgM-HRP antibody (1:1000, SouthernBiotech, 1020-05), HRP-conjugated rabbit anti–β-actin monoclonal antibody (1:5000, HUABIO, ET1702-67), total OXPHOS rodent WB antibody cocktail (1:1000, Abcam, MS604-300), rabbit anti-*Pcbp1* antibody (1:1000, MBL, RN0249P), rabbit anti-VDAC1 monoclonal antibody (1:1000, HUABIO, ET1601-20), rabbit anti-FdxR polyclonal antibody (1:1000, ProteinTech, 15584-1-AP), HRP-conjugated rabbit anti-GAPDH monoclonal antibody (1:5000, HUABIO, ET1702-66), HRP-conjugated goat anti-rabbit IgG antibody (1:5000, HUABIO, HA1001), and HRP-conjugated goat anti-mouse IgG polyclonal antibody (1:5000, HUABIO, HA1006).

### Seahorse Mito stress assay

Analysis of mitochondrial respiration and glycolytic rate of WT or *Pcbp1*-deficient B was performed using a Seahorse XFe96 extracellular flux analyzer with a Seahorse XF Cell Mito Stress Test Kit following the manufacturer’s instructions. The Seahorse XFe96 FluxPak cartridge was hydrated with distilled water overnight at 37°C (no CO_2_) and equilibrated for 1 hour on the day of the assay in Seahorse XF Calibrant solution. Vessels were precoated with Cell-Tak (22.4 mg/ml) for 20 min at 37°C to ensure continued adhesion of cytotoxic T lymphocytes on the plate during the assay. For OCR measurements, RPMI 1640 medium (pH 7.4) was supplemented with 10 mM glucose, 1 mM sodium pyruvate, and 2 mM l-glutamine, with sequential injection of 1 μM oligomycin, 1 μM FCCP, and 0.5 μM rotenone/antimycin A. For ECAR measurements, RPMI 1640 (pH 7.4) contained 2 mM l-glutamine, with injection of 10 mM glucose, 1 μM oligomycin, and 50 mM 2-deoxy-d-glucose to assess glycolysis parameters. Seahorse data were collected using Wave Controller 2.6 (Agilent).

### In vitro iGCB cell differentiation

40LB culturing system was conducted as described previously (*46*). Briefly, naïve B cells from WT or *Pcbp1* BKO mice were seeded on 40LB-IL4 feeder cells (NIH 3T3 cells expressing CD40L, BAFF, and IL-4) for iGCB differentiation. Four days later, the percentage of iGCB was determined by Fas and GL7 staining.

### Immunization

For NP-KLH immunization, 100 μl of NP-KLH (1 mg/ml; Biosearch, N5060) and 100 μl of album adjuvant (Thermo Fisher Scientific, 77161) were gently mixed upside down at room temperature for 30 min. Two hundred microliters of emulsified NP-KLH was injected intraperitoneally into the mice. Serum was collected on day 7 or 13 after immunization. For MitoTEMPO treatment, the mice were administered MitoTempo (5 mg/kg) (body weight) via intraperitoneal injection from day −7 to day 13 daily.

For SRBC immunization, 1 ml of citrated sheep blood was washed twice by ice cold PBS and resuspended 1:10 in PBS to make SRBC suspension. Two hundred microliters of the SRBC suspension was injected intraperitoneally into the mice. Serum was collected on day 5 and day 10.

### MzB cell depletion

WT and *Pcbp1* BKO mice were intraperitoneally injected with 100 μg of each anti-CD11a (InVivoMab, clone M17/4) and anti-CD49d (InVivoMab, clone PS/2) or isotype-control antibodies (IgG2a, InVivoMab). Mice were analyzed 7 days posttreatment.

### Enzyme-linked immunosorbent assay

To quantify levels of serum Ig, Nunc 96-well Multisorp plates (BBI Life Science) were coated with anti-mouse Ig antibody (Southern Biotech, 1010-01) in PBS and stored at 4°C. On the day of usage, the plates were washed with ELISA wash buffer (1× PBS 0.025% Tween-20) at the start and between each step. The plates were blocked with 1% FBS in PBS for 2 hours at 37°C. ELISA standards (Southern Biotech) were prepared according to the manufacturer’s instructions. Samples, ELISA standards, and secondary antibodies were appropriately diluted in 1% FBS, run in triplicates and, at each step, incubated for 1 hour at room temperature with light shaking. After incubation, the plates were washed again with PBST (PBS+0.05% Tween-20) five times, and HRP-conjugated anti-mouse IgM (0.5 μg/ml; Southern Biotech, 1010-01), IgG1 (Southern Biotech, 1070-01), IgG2b (Southern Biotech, 1090-01), IgG2c (Southern Biotech, 1077-01), IgG3 (Southern Biotech, 1100-01), IgA (Southern Biotech, 1040-01), or IgE (Southern Biotech, 1160-01) was added into each well for 1-hour incubation. For development, plates were incubated with trimethylboron (TMB) substrate (BioLegend, 421101) at room temperature in the dark for 1 to 5 min. The reaction was stopped with equal volume of 1 M sulfuric acid and absorbance read at 450 nm on a plate reader (Thermo Fisher Scientific).

To measure NP-specific antibody titer in the serum, 96-well plates were precoated with NP–bovine serum albumin (BSA) (NP9-BSA, N-5050 L-10; NP27-BSA, and N-5050H-10; 10 μg/ml) in PBS at 4°C overnight. Plates were washed with PBST three times and blocked with blocking buffer (1% FBS in PBS) at 37°C for 2 hours. The serum was diluted with antibody-dilution buffer (1% FBS in PBS) and was incubated with the plates coated with the antigen at room temperature for 1 hour. After incubation, the plates were washed again with PBST five times and HRP-conjugated anti-mouse IgG1 (0.5 μg/ml; Southern Biotech, 5300-05B), IgG3 (Southern Biotech, 5300-05B) and was added into each well for 1-hour incubation. After sufficiently washed with PBST, the plates were developed with TMB substrate (BioLegend, 421101) for 1 to 5 min, and chromogenic reaction was stopped with echo volume of 1 M sulfuric acid. Absorbance was determined by Thermo Fisher Scientific, Varioskan LUX multimode microplate reader.

### ELISPOT assay

To quantify bone marrow ASCs producing high-affinity (NP9) and low-affinity (NP27) anti-NP IgG, as well as total IgG, ELISPOT assays were performed 13 days postimmunization with NP-KLH in Alum. Bone marrow cells were isolated from WT and *AID^Cre/+^Pcbp1^fl/fl^* (*Pcbp1* GCB KO) mice. Multiscreen-HA 96-well filter plates (Millipore, MAHA) were coated overnight at 4°C with goat anti-mouse Ig (50 μg/ml), NP27-BSA (50 μg/ml), or NP7-BSA (50 μg/ml) in PBS. Plates were washed twice with PBS containing 0.05% Tween-20 (PBST) and blocked with DMEM supplemented with 10% FBS at 37°C for 2 hours. Bone marrow cells (1 × 10^6^ per well) were plated in duplicate and incubated at 37°C for 5 hours in a 5% CO_2_ incubator. Plates were then washed three times with PBS and PBST, followed by overnight incubation at 4°C with alkaline phosphatase–conjugated anti-mouse IgG (0.5 μg/ml; Sigma-Aldrich). The following day, the plates were developed using BCIP/NBT-plus substrate (Mabtech) until distinct spots appeared, according to the manufacturer’s instructions. ASCs were visualized as dark blue spots and quantified using ImageJ software (NIH). To assess differences in antibody affinity, the ratio of NP9 (high-affinity) to NP27 (low-affinity) ASCs was calculated for WT and *Pcbp1* GCB KO mice.

### LPS treatment

Naïve B cells purified by negative selection (Stem Cell Technology) were cultured at 1 × 10^6^ per well in 12-well plates at a 1-ml final volume. The following reagents were added for activation: LPS (Sigma-Aldrich, at 20 μg/ml), murine rIL-2 (Novoprotein) at 200 U, and murine rIL-5 (Novoprotein, at 5 ng/ml). Then, IgM expression was analyzed at days 1, 2, and 3 by flow cytometry.

### Immunofluorescence imaging

Thirteen days after NP-KLH immunization, spleen tissue cryosections were fixed with 1% paraformaldehyde (PFA) and permeabilized with 0.4% Triton X-100. Following this, the sections were blocked with 10% goat serum in PBST for 2 hours. Primary antibodies, specifically rat anti-mouse IgD BV450 (1:100), rat anti-mouse CD21/CD35 APC (1:100), and rabbit anti-mouse Ki67 (1:1000), were incubated for 1 hour at room temperature in a dark environment. After three washes with PBST, each lasting 5 min, the sections were incubated with the secondary antibody, goat anti-rabbit IgG Cy3(1:1000), for another hour at room temperature while avoiding light. The sections were then washed three more times with PBST, mounted, and imaged using a fluorescence microscope.

### Transmission electron microscopy

iGCB cells differentiated for 3 days were collected for mitochondrial morphology study. At first, dead cells or cellular debris were removed via Ficoll density gradient centrifugation, 2 × 10^6^ cells were fixed in 2% PFA + 2.5% glutaraldehyde buffer (pH 7.2) overnight, and then washed and postfixed in 1% osmium followed by 1% uranyl acetate, and processed for EPON embedding. Sections (about 70 nm) were poststained with lead citrate and viewed using a Thermo Fisher Scientific Talos L120C TEM microscope at 80 kV. Images were captured using a 4 k by 4 k Ceta charge-coupled device camera and the Thermo Fisher Scientific Velox software (version 2.8.0.898). For quantitation of mitochondria phenotype, images were collected of every cell profile within four (WT = 255 cells total, KO = 215 cells total) grid squares of a single thin section (about 70 nm) of each of the WT and KO GCB samples. Each cell profile was scored for mitochondrial morphology as follows: none (no mitochondria), normal (normal mitochondria), and disrupted (disrupted mitochondria), and the percentage of disrupted mitochondria was compared between WT and KO GCB cells.

### Bone marrow chimera

Bone marrow cells were isolated from *Pcbp1* BKO mice pretreated with 5-fluorouracil (150 mg/kg) 3 days before collection. The cells were cultured in Iscove’s modified Dulbecco’s medium (IMDM) with IL-3 (10 ng/ml), IL-6 (20 ng/ml), and stem cell factor (SCF, 50 ng/ml) and transduced with *Fdxr* overexpression or vector retrovirus at 20 and 40 hours after culture. Puro (2 mg/ml) was added to the culture on day 3. Cells were harvested on day 5, and 2.5 × 10^6^ cells were intravenously injected into μMT mice that had been sublethally irradiated (400 rad) 1 day before cell transfer. Six weeks after the transplantation, the chimeric mice were challenged with NP-KLH.

### Retroviral infection in bone marrow cells

Retroviruses were produced by transfecting Plat-E cells with the indicated plasmids. Fresh virus supernatant was collected 2 days after transfection and used to infect bone marrow cells at 20 and 40 hours after culture. After spin infection for 1.5 hours at 900*g*, bone marrow cells were cultured at 37°C for an additional 4 hours, and then the media were exchanged with fresh cell culture media containing IL-6, IL-3, and SCF.

### Viral infection in Namalwa B cells

To overexpress *Pcbp1* or *Fdxr* in Namalwa B cells, retroviruses were generated by transfecting 293T cells with pCL-10A1 and Migr1-OE plasmids, and virus supernatant was collected at 48 and 72 hours. Then, Namalwa B cells were spin-infected with the supernatant (900*g* for 90 min at 37°C), and polybrene (8 μg/ml) was used to enhance transduction. For gene deletion, 293T cells were transfected with LV9-shRNA, VSVG, and psPAX2 plasmids to produce lentivirus; the supernatant was collected at 48 and 72 hours; and Namalwa B cells were infected with it. The sequence of *Pcbp1* and *Fdxr* are listed in table S3.

### TMT mass spectrometry

Naïve B cells from WT or *Pcbp1* BKO mice were lysed in 1 ml of RIPA lysis buffer and then sonicated with a BioRuptor. After quantification via BCA assay (Thermo Fisher Scientific), 100 μg of protein was reduced in 10 mM DTT at 55°C for 45 min followed by alkylation in 50 mM iodoacetamide at room temperature in the dark for 30 min. Protein was precipitated using acetone and then resuspended in 100 mM triethylamonium bicarbonate. Trypsin was added at a 1:100 ratio (w/w, enzyme/protein), and the mixture was then incubated at 37°C overnight in the ThermoMixer R with shaking at 1000 rpm. After digestion, peptides were proceeded with isobaric peptide labeling with TMT6plex reagents (catalog no. 90061, Thermo Fisher Scientific). Labeled peptides were combined and desalted using an HLB cartridge (WAT094225, Waters), and the sample was injected onto a Waters XBridge BEH C18 4.6 mm–by–250 mm column using a Thermo Fisher Scientific Dinex Ultimate 3000. Bound peptides were separated using a 40-min gradient. Twenty fractions were collected and concatenated into five total fractions postrun.

The peptides were separated by a 130-min gradient elution at a flow rate 0.300 μl/min with the DIONEX UltiMate 3000 integrated nano-HPLC system, which is directly interfaced with the Thermo Fisher Scientific Orbitrap Fusion Lumos Tribrid mass spectrometer equipped with FAIMS Pro. The analytical column was a homemade fused silica capillary column (75-μm ID, 150-mm length; Upchurch, Oak Harbor, WA) packed with C-18 resin (300 A, 3 μm, Varian, Lexington, MA). Mobile phase A consisted of 0.1% formic acid, and mobile phase B consisted of 80% acetonitrile and 0.1% formic acid.

The differential protein expression of mass spectrometry proteomics data was analyzed by DEP (*58*) R package. Differential enrichment tests are based on protein-wise linear models and empirical Bayes statistics using limma. False discovery rates are estimated using fdr tool. The data are presented in table S1.

### Luciferase assay

To identify *Pcbp1* regulation for *Fdxr*, *Fdxr*- 3′UTR (WT/mutant) luciferase reporters were performed in WT *or Pcbp1*-deficient 293T cells. Briefly, a suspension of 293T cells was seeded to each well of a six-well plate at a density of ∼1.0 × 10^6^ cells/ml and incubated for 16 to 20 hours. Then, each well was transfected with 250 ng of pGL3 (*Fdxr*- -3′UTR)-Luc, 50 ng of renilla, and 2 μg of sh*Ctrl* of sh*Pcbp1*. Forty-eight hours after the transfection, activity of firefly luciferase (FL) and Renilla luciferase (RL) was determined with a dual-luciferase reporter assay (Promega), and FL activity was normalized to RL activity. The sequences of the WT and mutant *Fdxr* 3′UTRs are provided in table S3.

### Puro incorporation

For Op-puro incorporation, 1 × 10^6^ B cells from WT or *Pcbp1* BKO mice are treated with LPS (Sigma-Aldrich, at 20 μg/ml), murine rIL-2 (Novoprotein, at 200 U), and murine rIL-5 (Novoprotein, at 5 ng/ml) for 20 hours. The cells are then incubated with 10 μM op-puro (APExBIO, A8778) for 3 hours. After incubation, the cells are washed with precooled PBS and stained for surface markers for 30 min at 4°C. The cell pellets were resuspended in 200 μl of 3.7% formaldehyde (Sigma-Aldrich) for 15 min on ice and then permeabilized with 0.2% Triton X-100 for 25 min at room temperature. The Click-iT reaction mixture (Thermo Fisher Scientific) was prepared by combining 440 μl of 1× Click-iT Reaction Buffer (component A), 10 μl of CuSO_4_ (component B), 2.5 μl of Alexa Fluor 555 (AF555), and 50 μl of 1× Click-iT Cell Buffer Additive (component C). A total of 500 μl of the Click-iT reaction mixture was added to each sample and incubated for 30 min at room temperature. After the reaction, the cells were washed twice with PBS containing 1% FBS and analyzed by flow cytometry.

To assess protein synthesis in GCB, FoB, and MzB cells, splenocytes were cultured in complete RPMI 1640 medium supplemented with 10% FBS, 1% penicillin-streptomycin, and Puro (10 μg/ml) for indicated hours at 37°C in a 5% CO_2_ incubator. After incubation, the cells were harvested, washed with PBS, fixed, and permeabilized. The cells were then stained intracellularly with Alexa Fluor 647 anti-Puro antibody (clone 2A4, BioLegend, catalog no. 22088) and surface-stained with fluorochrome-conjugated antibodies against CD19, B220, GL7, CD95, CD23, and CD21 to identify GCB (CD19^+^B220^+^GL7^+^CD95^+^), FoB (CD19^+^B220^+^CD23^high^CD21^low^), and MzB (CD19^+^B220^+^CD23^low^CD21^high^) cells. Antibodies were used at optimal dilutions as recommended by the manufacturer, and the cells were incubated with antibodies for 30 min at 4°C in the dark. Stained cells were washed, resuspended in flow cytometry buffer, and analyzed using a flow cytometer. Data were analyzed using FlowJo software, and the mean fluorescence intensity (MFI) of Puro was determined for each cell population.

To evaluate the effects of mt-ROS scavenging on protein synthesis, splenocytes were resuspended in complete RPMI 1640 medium at 1 × 10^6^ cells/ml and incubated with 100 μM MitoTEMPO for 3 hours at 37°C in 5% CO_2_. The cells were then washed twice with complete RPMI 1640 to remove MitoTEMPO, followed by incubation with Puro (10 μg/ml) for 2 hours. Fow cytometry staining of Puro incorporation was performed as described above, and Puro MFI was quantified in FoB populations using FlowJo software.

### Bulk RNA-seq

For transcriptional analysis, total RNA extracted from naïve B cells or iGCB cells in WT or *Pcbp1* BKO mice and RNA quality and quantity were measured using Agilent Bioanalyzer. mRNA libraries were prepared using NEB RNA library Prep Kit (NEB, E7530S). DNA libraries were sequenced on a Hiseq 2000 platform or Hiseq X-10 platform. Two pairs of naïve B cells or three pairs of iGCB samples were included for analysis. For RNA-seq analysis, adapter sequences were first removed from the reads in the high-throughput sequencing data using Trim Galore (v.0.6.10). The clean reads were then mapped to the mouse genome (mm10) using STAR (v.2.7.11a) with GENCODE transcript annotation (*59*). Overlapping reads were counted and summarized by gene with featureCounts (*60*). DEGs between datasets were identified using the R package DESeq2 (v.1.40.2). DEGs were classified on the basis of a twofold change (up or down) with a *P* value <0.05 and an absolute log_2_ fold change (|LFC|) > 0.5. Plots were generated using R (v.4.2.1). Heat maps were created with Cluster3 and Java Treeview (*61*), and genes and samples were clustered using hierarchical clustering. GO analysis was performed using Metascape.

### scRNA-seq and data analysis

Droplet-based scRNA-seq datasets were generated using a Chromium system from 10x Genomics. Specifically, flow-sorted GC B cells (CD19^+^ B220^+^ NP^+^ GL7^+^ CD95^+^) from WT or *Pcbp1* GCB KO mice were suspended in PBS with 0.4% FBS, and the concentration was adjusted to capture 10,000 cells. The cells were then loaded into the GemCode instrument, where they were lysed and mixed with beads carrying unique barcodes in individual oil droplets. Following this, reverse transcription, emulsion breaking, cDNA amplification, shearing, and 5′ adapter and sample index attachment were performed. Libraries were sequenced in paired-end mode (150 + 150 bp) on the NovaSeq 6000 platform from Illumina.

For initial processing and gene expression estimation, we used Cell Ranger (v.6.1.2). Each library was aligned to an indexed mm10 genome using Cell Ranger Count. The resulting matrix was then imported into the Seurat R toolkit (v.4.3.0.1) for quality control and downstream analysis. Doublets were removed using Scrublet (v. 0.2.3) (*62*). The unique molecular identifier (UMI) count matrix was converted to Seurat objects in RStudio (v. 2023.09.1 + 494) using the Seurat package. Cells with fewer than 500 genes, more than 20,000 UMIs, or more than 20% of UMIs mapped to mitochondrial genes were excluded to remove red blood cell contamination. This process yielded profiles for 7464 WT and 4951 GC-KO cells.

We performed anchoring and integration of the WT and *Pcbp1* GCB KO B cell datasets using Seurat (version 4.3.01) and R (version 4.2.1) (*63*). The merged Seurat objects were normalized, and highly variable genes were identified and scaled with SCTransform (*64*). The top 3000 highly variable features were used for anchoring, and integration anchors (30 dimensions) were computed for data integration. For neighbor and cluster identification, the integrated object was scaled, and significant principal components (PCs) were determined through statistical and heuristic testing as recommended by Seurat. The clustered cells were visualized using Uniform Manifold Approximation and Projection (UMAP). The first 15 PCs were used for UMAP visualization and graph-based clustering.

DEGs were identified using the Wilcoxon rank-sum test, implemented in the Seurat functions FindMarkers and FindAllMarkers. Genes with *P* < 0.05, absolute log_2_ fold change >0.25, and minimum fraction >0.1 were considered significant DEGs, unless specified otherwise. In addition, enrichment analysis for multiple DEG lists was performed using Metascape (https://metascape.org) (*65*).

### RIP assay

iGCB (5 × 10^6^) cells differentiated for 4 days were lysed with lysis buffer [50 mM tris-HCl (pH 7.4), 100 mM NaCl, 1% NP-40, 0.1% SDS, 0.5% sodium deoxycholate, and protease Inhibitor Cocktail] and immunoprecipitation was performed with an anti-PCBP1 antibody (MBL) or normal rabbit IgG and Dynabeads M-270 Epoxy (Invitrogen, 14301) overnight at 4°C and then washed with high-salt buffer [50 mM tris-HCl (pH 7.4), 1 M NaCl, 1 mM EDTA, 1% NP-40, 0.1% SDS, and 0.5% sodium deoxycholate] and low-salt buffer [20 mM tris-HCl (pH 7.4), 10 mM MgCl_2_, and 0.2% Tween-20]. RNA was extracted and used to perform RIP-seq and real-time PCR with specific primers (table S2).

### Digital PCR assay

Total RNA naïve B cells from WT or *Pcbp1* BKO mice was isolated with HP total RNA kit (Omega), and cDNA was synthesized using PrimeScript RT reagent kit with gDNA Eraser (Takara). The synthesized cDNA was used as a template for digital PCR on the naica system. Each 25-μl reaction mixture contained 5 μl of 5× Perfecta Multiplex qScript ToughMix, 2.5 μl of 1 μM fluorescein, 1 μl of 25× Probes and Primer Mix, 1 μl of DNA template, and nuclease-free water to a final volume of 25 μl. The reactions were loaded into the wells of a Sapphire chip and subjected to the following thermal cycling conditions on the Naica Geode: 95°C for 10 min, followed by 45 cycles of 95°C for 30 s and 58°C for 15 s.

Scanning parameters were 65 nm for FAM (Gapdh) and 250 nm for VIC (IgM). Gene expression was normalized to Gapdh. The primers are listed in table S2.

### Plasmids

To generate MIGR1-*Pcbp1* OE-GFP or MIGR1-*Fdxr* OE-GFP plasmids, *Pcbp1* and Fdxr CDS were amplified from human or mouse cDNA and inserted between Xho1 and EcoR1 sites of MIGR1 vector. For luciferase reporter plasmids, WT or mutant *Fdxr* 3′UTR sequence was inserted between the XbalI sites of pGL3 basic vector (Promega). PMXS-NDI1 plasmid were got from Addgene (#72876). LV9-shRNA and LV10-shRNA plasmids were obtained from GenePharma (Shanghai, China), shRNA sequences were provided in table S3.

### NAD^+^/NADH measurement

Naïve B cells from the spleens of WT or *Pcbp1* BKO mice were purified by negative selection (Stem Cell Technology, 19854A) and stimulated with LPS, IL-2, and IL-5 for 72 hours, and the intracellular NAD^+^ and NADH levels were further quantified using an NAD^+^/NADH assay kit (Abcam, catalog no. ab65348). Briefly, 7.5 × 10^6^ naïve B cells or 1 × 10^6^ LPS-stimulated B cells with dead cells removed via Ficoll density gradient centrifugation were lysed in 450 μl of extraction buffer II, and the lysate was divided into two aliquots for total NAD^+^ (total NAD and NADH) and NADH measurements, respectively. For NADH detection, the corresponding aliquots were heated at 60°C for 30 min to decompose NAD^+^. To each sample well, 98 μl of cycling buffer I and 2 μl of NAD cycling enzyme mix were added, followed by 10 μl of developer solution II/NADH developer after 5 min. The reaction was incubated at room temperature for 3 hours, and the optical density was measured at 450 nm using a microplate reader. NAD^+^/NADH ratios were calculated using the formula: NAD^+^/NADH = (NADt − NADH) / NADH.

### ATP assay

iGCB cells differentiated for 4 days were collected with dead cells removed via Ficoll density gradient centrifugation, and the intracellular ATP levels were measured using a Luminescent ATP detection assay kit (Abcam, catalog no. ab113849). Briefly, 4 × 10^4^ live cells in 100 μl Dulbecco’s phosphate buffered saline (DPBS) per well were lysed with 50 μl of detergent for 5 min in an orbital shaker at 700 rpm, followed by 50 μl of substrate solution for another 5 min. The reaction was further incubated at room temperature in the dark for 10 min, and the luminescence was measured with a microplate reader. The amount of ATP in the sample well was calculated from the standard curve (micromolar).

### Statistical analysis

Statistical tests performed by GraphPad Prism 8 included the two-tailed, unpaired, two-sample *t* test or Dunnett’s multiple comparisons test after one-way analysis of variance (ANOVA). Data are presented as means ± SD from independent experiments.
