# Supplementary material for: RNA binding protein Pcbp1 maintains mitochondria integrity to promote antibody production and germinal center response
Source: Sci Adv. 2026 Apr 10;12(15):eadz9095. doi: 10.1126/sciadv.adz9095 (PMC13068074; doi:10.1126/sciadv.adz9095)
Supplement: Supplementary file 1 — Figs. S1 to S13 Tables S1 to S4 [file sciadv.adz9095_sm.pdf]

Science Advances
AAAS

Supplementary Materials for

RNA binding protein *Pcbp1* maintains mitochondria integrity to promote antibody production and germinal center response

Lizhen Zhu *et al.*

Corresponding author: Xing Chang, changxing@westlake.edu.cn

Sci. Adv. **12**, eadz9095 (2026)
DOI: 10.1126/sciadv.adz9095

This PDF file includes:

Figs. S1 to S13
Tables S1 to S4

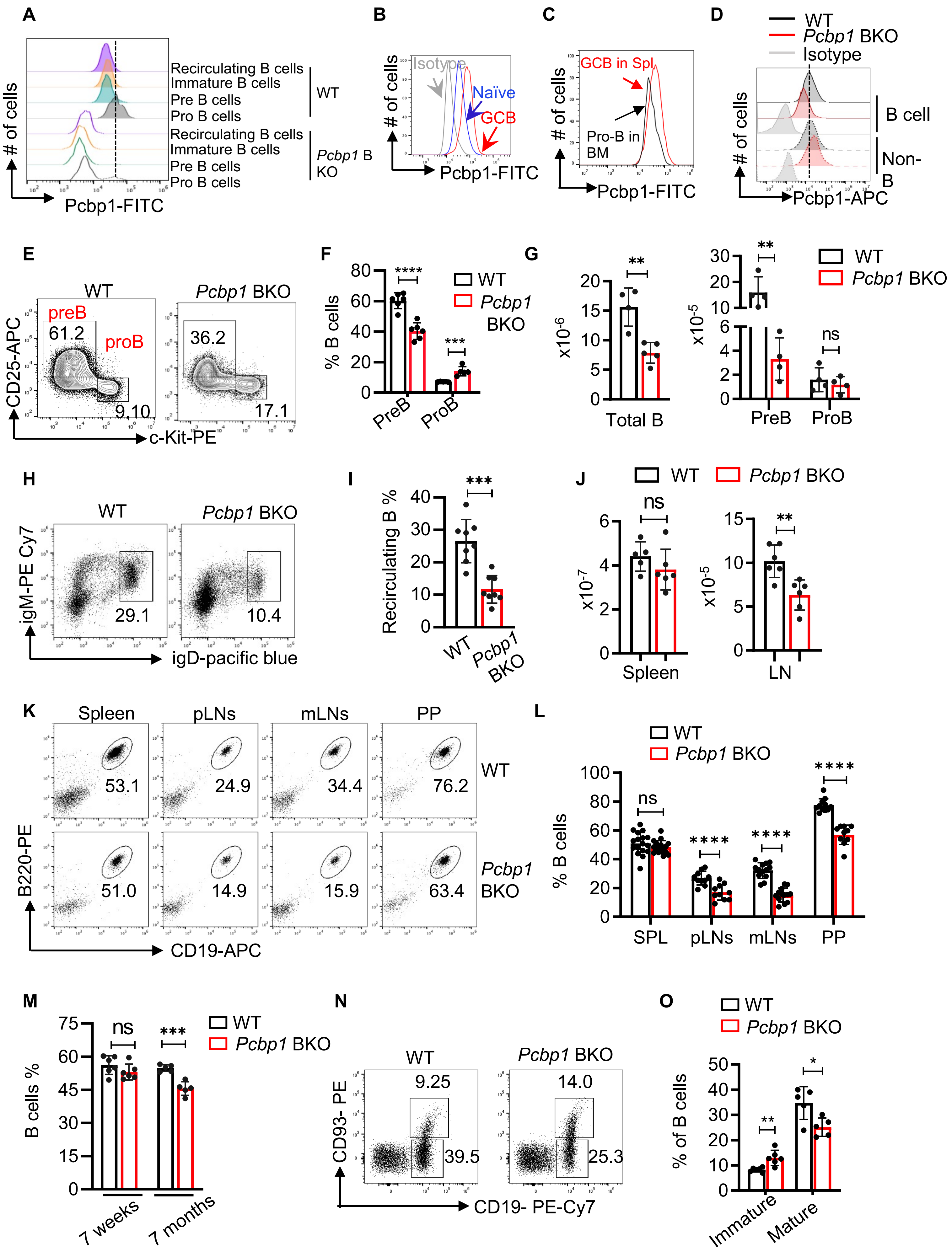

A
Recirculating B cells
Immature B cells
Pre B cells
Pro B cells
WT
Recirculating B cells
Immature B cells
Pre B cells
Pro B cells
Pcbp1 B KO
# of cells
Pcbp1-FITC
B
Isotype
Naïve
GCB
# of cells
Pcbp1-FITC
C
GCB in Spl
Pro-B in BM
# of cells
Pcbp1-FITC
D
WT
Pcbp1 BKO
Isotype
B cell
Non-B
# of cells
Pcbp1-APC
E
WT
Pcbp1 BKO
preB
proB
61.2
9.10
36.2
17.1
CD25-APC
c-Kit-PE
F
% B cells
PreB
ProB
WT
Pcbp1 BKO
G
x10-6
Total B
x10-5
PreB
ProB
WT
Pcbp1 BKO
H
WT
Pcbp1 BKO
29.1
10.4
igM-PE Cy7
igD-pacific blue
I
Recirculating B %
WT
Pcbp1 BKO
J
WT
Pcbp1 BKO
x10-7
Spleen
x10-5
LN
K
Spleen
pLNs
mLNs
PP
53.1
24.9
34.4
76.2
WT
51.0
14.9
15.9
63.4
Pcbp1 BKO
B220-PE
CD19-APC
L
WT
Pcbp1 BKO
% B cells
SPL
pLNs
mLNs
PP
M
B cells %
7 weeks
7 months
WT
Pcbp1 BKO
N
WT
Pcbp1 BKO
9.25
39.5
14.0
25.3
CD93- PE
CD19- PE-Cy7
O
WT
Pcbp1 BKO
% of B cells
Immature
Mature

Figure S1. *Pcbp1* deficiency resulted in a partial blockade of B cell development in the bone marrow.

(**A**). *Pcbp1* expression level was determined via intracellular staining across different stages of B cell development. Shown is representative flow cytometric analysis of *Pcbp1* level among pro-B cells ($B220^+IgM^-CD25^-c\text{-}Kit^+$), pre-B cells ($B220^+IgM^-CD25^+c\text{-}Kit^-$), immature B cells ($B220^+IgM^+IgD^-$), recirculating B cells ($B220^+IgM^+ IgD^+$) in WT and *Pcbp1* BKO mice from three independent experiments.

(**B**). *Pcbp1* was elevated in GCB cells. 13 days after WT mice immunized with NP-KLH in Alum, *Pcbp1* levels in follicular B cells ($CD19^+B220^+GL7^-CD95^-$) and GCB cells ($CD19^+B220^+GL7^+CD95^+$) were detected by flow cytometry. Shown are representatives from five independent experiments.

(**C**). As in (**B**), *Pcbp1* levels in spleen GCB cells ($CD19^+B220^+GL7^+CD95^+$) were compared to bone marrow pro-B cells ($B220^+IgM^-CD25^-c\text{-}Kit^+$). Data represents results from four independent experiments.

(**D**). Specific depletion of *Pcbp1* in B cells of $MB1^{Cre/+}$*Pcbp1*$^{fl/fl}$ (*Pcbp1* BKO) mice. *Pcbp1* expression in B cells ($CD45^+B220^+CD19^+$) and non-B cells ($CD45^+B220^-CD19^-$) of *Pcbp1* BKO mice was determined by intracellular staining. Data are representative of three independent experiments.

(**E-G**). *Pcbp1*-deficient B cells were partially arrested at the transition from pro-B to pre-B cell stage. (**E**). Flow cytometric analysis of pro-B cells ($B220^+IgM^-CD25^-c\text{-}Kit^+$) and pre-B cells ($B220^+IgM^-CD25^+c\text{-}Kit^-$) in the bone marrow of WT or *Pcbp1* BKO mice. (**F**). Summary of pro-B cell and pre-B cell percentages within total B cells in the bone marrow from three independent experiments. (**G**). Total B cell numbers in the bone marrow (*left*), along with pro-B and pre-B cell counts, were quantified and summarized from three independent experiments. Each dot represents one individual mouse. Statistical significance was determined using Student's *t*-test. **, $p < 0.01$, ***, $p<0.001$, and ****, $p<0.0001$.

(**H, I**). *Pcbp1* deletion diminished recirculating B cells in the bone marrow. (**H**) Representative flow cytometry analysis of recirculating B cells (IgM^+IgD^+) in WT or *Pcbp1* BKO mice. (**I**) Summary of recirculating B cell populations from three independent experiments. Statistical significance was determined using Student's *t*-test. ***, $p<0.001$.

(**J-L**). *Pcbp1* deletion slightly reduced B cell ratios in multiple lymphoid organs. (**J**). Total B cell counts in spleens and lymph nodes summarized from three independent experiments. (**K**). Flow cytometry analysis of B cells ($CD19^+B220^+$) from spleen (SPL), peripheral lymph nodes (pLNs), mesenteric lymph nodes (mLNs), or Peyer's patches (PP) of 6-8 weeks old WT or *Pcbp1* BKO mice. (**L**). Percentages of B cells within $CD45^+$ cells were summarized from three independent experiments. **, $p < 0.01$, ****, $p<0.0001$ in Student's *t* test.

(**M**). Reduction of B cells was intensified in aged *Pcbp1* BKO mice. Percentages of B cells in the spleen of wild-type (WT) and *Pcbp1* B cell knockout (KO) mice at 7 weeks or 7 months of age were analyzed by flow cytometry. Data are a summary of three independent experiments. ***, $p < 0.001$ in Student's *t* test.

(**N-O**). *Pcbp1* ablation led to a slight reduction of mature B cells. (**N**) Flow cytometry analysis of immature B cells ($CD19^+CD93^+$) and mature B cells ($CD19^+CD93^-$) from the spleen of 6-8 weeks old WT or *Pcbp1* B KO mice. (**O**). Percentages of immature and mature B cells within total B cell population were summarized from three independent experiments. Statistical analysis was performed using Student's *t*-test. *, $p < 0.05$, **, $p < 0.01$.

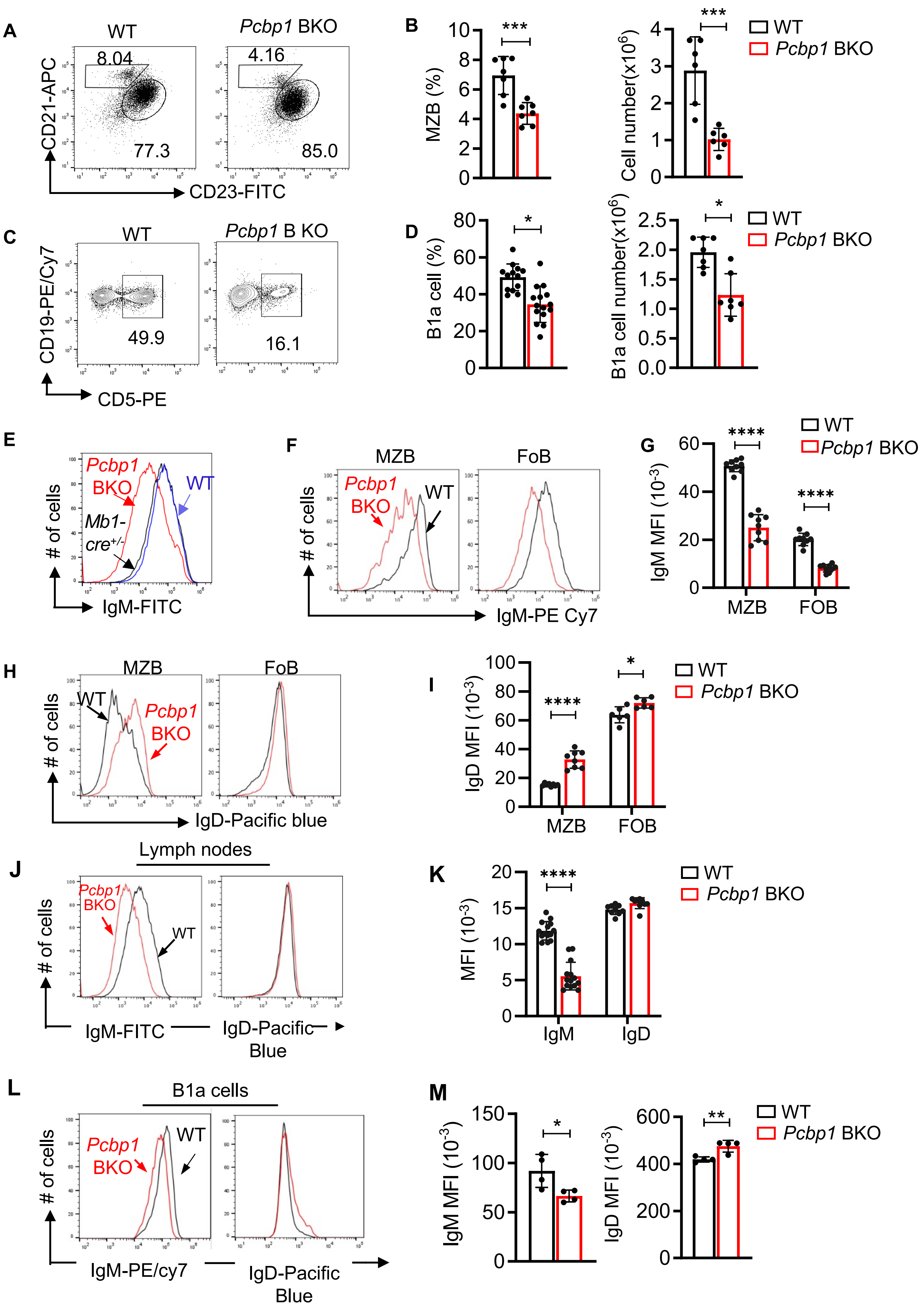

A
WT
Pcbp1 BKO
8.04
77.3
4.16
85.0
CD21-APC
CD23-FITC
B
MZB (%)
Cell number(x10^6)
WT
Pcbp1 BKO
C
WT
Pcbp1 B KO
49.9
16.1
CD19-PE/Cy7
CD5-PE
D
B1a cell (%)
B1a cell number(x10^6)
E
Pcbp1 BKO
WT
Mb1-cre+/-
# of cells
IgM-FITC
F
MZB
FoB
IgM-PE Cy7
G
IgM MFI (10^-3)
MZB
FOB
H
IgD-Pacific blue
I
IgD MFI (10^-3)
J
Lymph nodes
IgD-Pacific Blue
K
MFI (10^-3)
IgM
IgD
L
B1a cells
IgM-PE/cy7
M
IgM MFI (10^-3)
IgD MFI (10^-3)

Figure S2. *Pcbp1* promotes IgM expression across various B cell subsets

(**A, B**). *Pcbp1* deficiency resulted in diminished marginal zone B cells (MzB). (**A**) Flow cytometric analysis of MzB cells ($CD21^{+}CD23^{-}$) pregated from total B cells ($B220^{+}CD19^{+}$) in the spleen. (**B**) Summary of MzB ratios and cell numbers within the splenic B cells. Each dot represents one individual mouse. Statistical significance was determined using Student's *t*-test. ***, $p<0.001$.

(**C, D**). *Pcbp1* deficiency reduced peritoneal B1a cell frequency and numbers. (**C**) Flow cytometric analysis of ascites B1a cells ($CD19^{+}CD5^{+}$). (**D**) Quantification of B1a cell ratios (*left*) and absolute counts (*right*). Dots represent individual mice. *, $p<0.05$ in Student's *t*-test.

(**E**). Flow cytometry analysis of IgM expression in splenic B cells from WT control ($Pcbp1^{fl/fl}$), *Pcbp1* BKO, and Mb1-cre (*Mb1-Cre+/-*) mice. Data are representative from three independent experiments.

(**F-G**). *Pcbp1* promoted IgM expression in marginal zone B cells and follicular B cells. As in (**A**), IgM expression in gated follicular B cells and marginal zone B cells were determined via flow cytometry. Data are representative (**F**) or summary (**G**) of five independent experiments. ****, $p<0.0001$ in Student's *t* test.

(**H-I**). Elevated IgD expression in *Pcbp1*-deficient marginal zone B cells. As in (**A**), IgD expression in gated follicular B cells and marginal zone B cells were determined via flow cytometry. Data are representative (**H**) or summary (**I**) of five independent experiments. *, $p < 0.05$, ****, $p<0.0001$ in Student's *t* test.

(**J-K**). Diminished IgM expression in *Pcbp1*-deficient B cells in the lymph nodes. Surface IgM and IgD levels in gated B cells from lymph nodes were determined via flow cytometry. Data are representative (**J**) or summary (**K**) of three independent experiments. ****, $p<0.0001$ in Student's *t* test.

(**L-M**). *Pcbp1* promoted IgM expression in peritoneal B1a cells. B1a cells in ascites ($CD19^{+}CD5^{+}$) were gated from seven-week-old *Pcbp1* BKO or WT littermates. Surface IgM and IgD levels were determined by flow cytometry. Data are representative (**L**) or summary (**M**) of three independent experiments. *, $p<0.05$, **, $p<0.01$ in Student's *t* test.

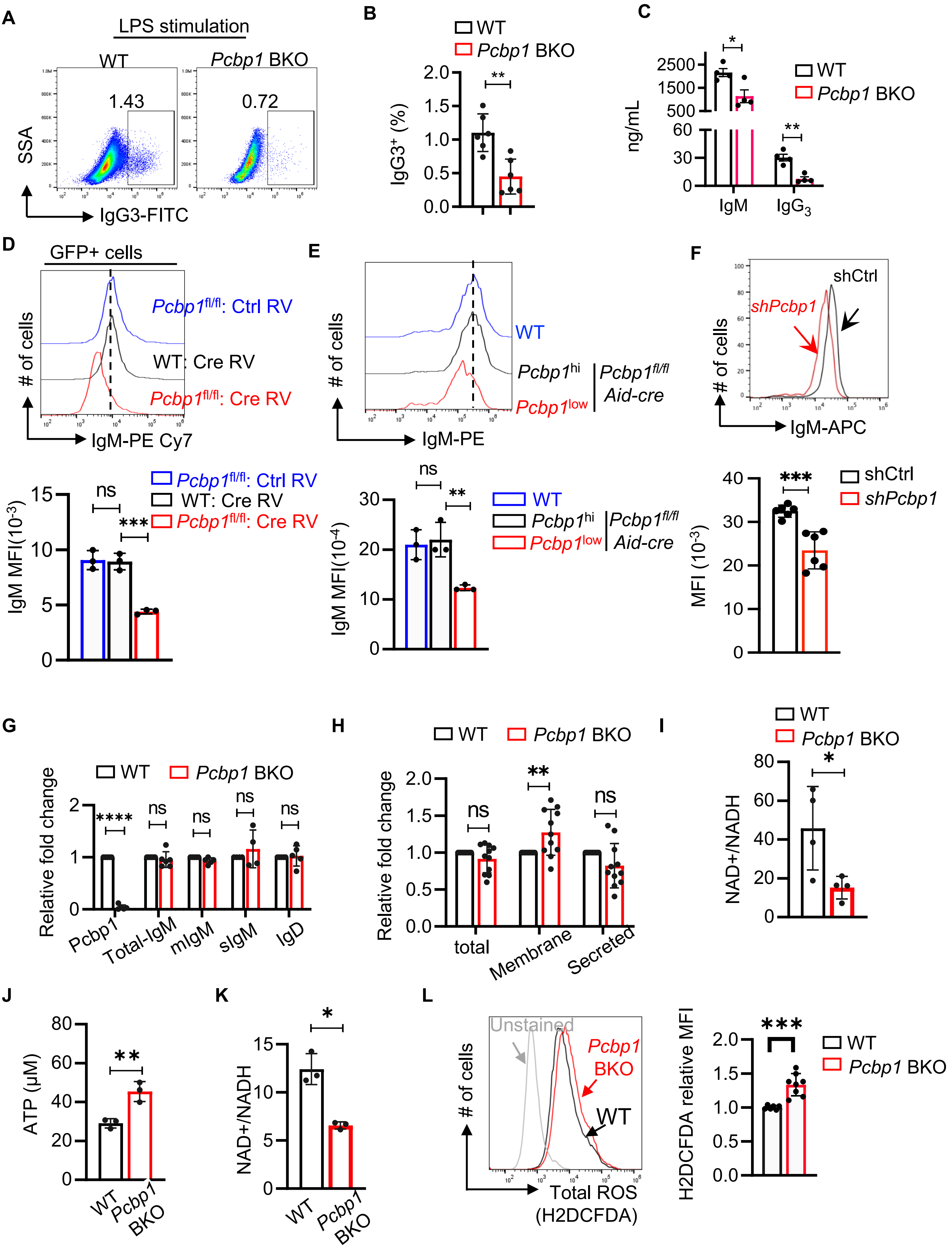

A
LPS stimulation
WT
Pcbp1 BKO
1.43
0.72
SSA
IgG3-FITC
B
WT
Pcbp1 BKO
IgG3+ (%)
**
C
ng/mL
WT
Pcbp1 BKO
IgM
IgG3
D
GFP+ cells
Pcbp1fl/fl: Ctrl RV
WT: Cre RV
Pcbp1fl/fl: Cre RV
# of cells
IgM-PE Cy7
IgM MFI(10-3)
ns
***
E
# of cells
WT
Pcbp1hi
Pcbp1low
Pcbp1fl/fl Aid-cre
IgM-PE
IgM MFI(10-4)
ns
**
F
shCtrl
shPcbp1
# of cells
IgM-APC
MFI (10-3)
***
G
WT
Pcbp1 BKO
Relative fold change
****
ns
Pcbp1
Total-IgM
mIgM
sIgM
IgD
H
WT
Pcbp1 BKO
Relative fold change
total
Membrane
Secreted
I
WT
Pcbp1 BKO
NAD+/NADH
*
J
ATP (μM)
**
WT
Pcbp1 BKO
K
NAD+/NADH
*
WT
Pcbp1 BKO
L
Unstained
Pcbp1 BKO
WT
# of cells
Total ROS (H2DCFDA)
H2DCFDA relative MFI
***
WT
Pcbp1 BKO

Figure S3. *Pcbp1* deficiency diminishes ETC complex I expression and results in mitochondria defects following various stimulation

(**A-C**). *Pcbp1* promoted IgG3 expression in B cells following LPS stimulation. Naïve B cells from WT or *Pcbp1* BKO mice were stimulated with LPS (20 μg/mL), IL2 (200 U/mL), and IL5 (5 ng/mL), and IgG3 expression was determined via intracellular staining. Data are representative (**A**) or summary (**B**) from three independent experiments. (**C**). Levels of IgM and IgG3 in the culture supernatant were determined via ELISA. *, $p<0.05$, **, $p<0.01$ in Student's *t* test.

(**D**). Acute loss of *Pcbp1* inhibited IgM expression in activated murine B cells *in vitro*. B cells isolated from $Pcbp1^{fl/fl}$ or WT mice were activated under *in vitro* germinal center B cell (iGCB) differentiation conditions and transduced with retroviruses expressing Cre–IRES–GFP or Ctrl–IRES–GFP. Three days after transduction, IgM expression in GFP^{+} cells was examined, which were shown as representative flow cytometry histograms (*top*) and quantification (*bottom*). Data are from three independent experiments. Statistical significance was assessed using Student's *t*-test. ***, $p < 0.001$.

(**E**). *In vitro* activation of Aid-Cre $Pcbp1^{flox/flox}$ primary B cells led to reduced IgM expression. B cells from $Pcbp1^{fl/fl}$ *Aid-cre* or WT mice were activated under iGCB conditions for four days. $Pcbp1^{low}$ or $Pcbp1^{hi}$ cells from $Pcbp1^{fl/fl}$ *Aid-cre* mice were identified and analyzed by flow cytometry. Comparison of IgM expression among WT, $Pcbp1^{low}$ or $Pcbp1^{hi}$ populations, shown as representative flow cytometry histograms (*top*) and quantification (*bottom*). Data are from three independent experiments. Statistical significance was assessed using Student's *t*-test. **, $p < 0.01$.

(**F**). Loss of *Pcbp1* inhibited IgM expression in Namalwa B cells. *Pcbp1* was knocked down using shRNA, and IgM levels were measured by flow cytometry. Shown are representative flow cytometry histogram (*top*) or statistical data summarized from five independent experiments (*bottom*). Data are presented as mean ± SD. Statistical significance was determined by Student's *t*-test. ***, $p < 0.001$.

(G). *Pcbp1* has no impact on the transcript levels of μ and δ transcripts. Indicated transcript levels derived from Ig μ and δ were determined in isolated splenic B cells with real-time PCR. Data represent the mean ± SD from three independent experiments. ****, $p < 0.0001$ in Student's *t* test.

(**H**). *Pcbp1* deficiency did not impact IgM transcript levels. Naïve B cells from wild-type (WT) or *Pcbp1* BKO mice were stimulated with LPS (20 μg/mL), IL2 (200 U/mL), and IL5 (5 ng/mL). mRNA levels for total IgM (IgM-total), membrane IgM (M-IgM), and secreted IgM (S-IgM) were analyzed by real-time PCR. Data are summarized from six independent experiments. **, $p < 0.01$ in Student's *t* test.

(**I**). *Pcbp1* deficiency decreased the cellular NAD^{+}/NADH ratio. Naïve B cells from wild-type (WT) and *Pcbp1* BKO mice were lysed, and the intracellular concentrations of NAD^{+} and NADH were measured using a colorimetric assay. Data are summarized from four independent experiments. *, $p<0.05$ in Student's *t* test.

(**J**). *Pcbp1* deficiency increased ATP production in iGCB cells. Naïve B cells from WT or *Pcbp1* BKO mice were cultured under iGCB condition for four days, intracellular ATP concentration was detected by a luminescent ATP detection assay kit. Data are summarized from three independent experiments. **, $p<0.01$ in Student's *t* test.

(**K**). *Pcbp1* deficiency decreased the cellular NAD^{+}/NADH ratio in activated B cells. As in **G**, Naïve B cells from wild-type (WT) and *Pcbp1* BKO mice were stimulated with LPS (20 μg/mL), IL2 (200 U/mL), and IL5 (5

ng/mL), then the intracellular concentrations of NAD^+ and NADH were measured using a colorimetric assay. Data are summarized from four independent experiments. *, $p<0.05$ in Student's *t* test.

(**L**). Loss of *Pcbp1* elevated total ROS levels in naïve B cells. Splenic naïve B cells from WT or *Pcbp1* BKO mice were stained with a cellular ROS indicator (carboxy-H2DCFDA) at 37°C for 30 minutes. Total ROS levels were measured via flow cytometry. *Left,* representative flow cytometric plots; *Right,* summary of MFI from three independent experiments. ***, $p<0.001$ in Student's *t* test.

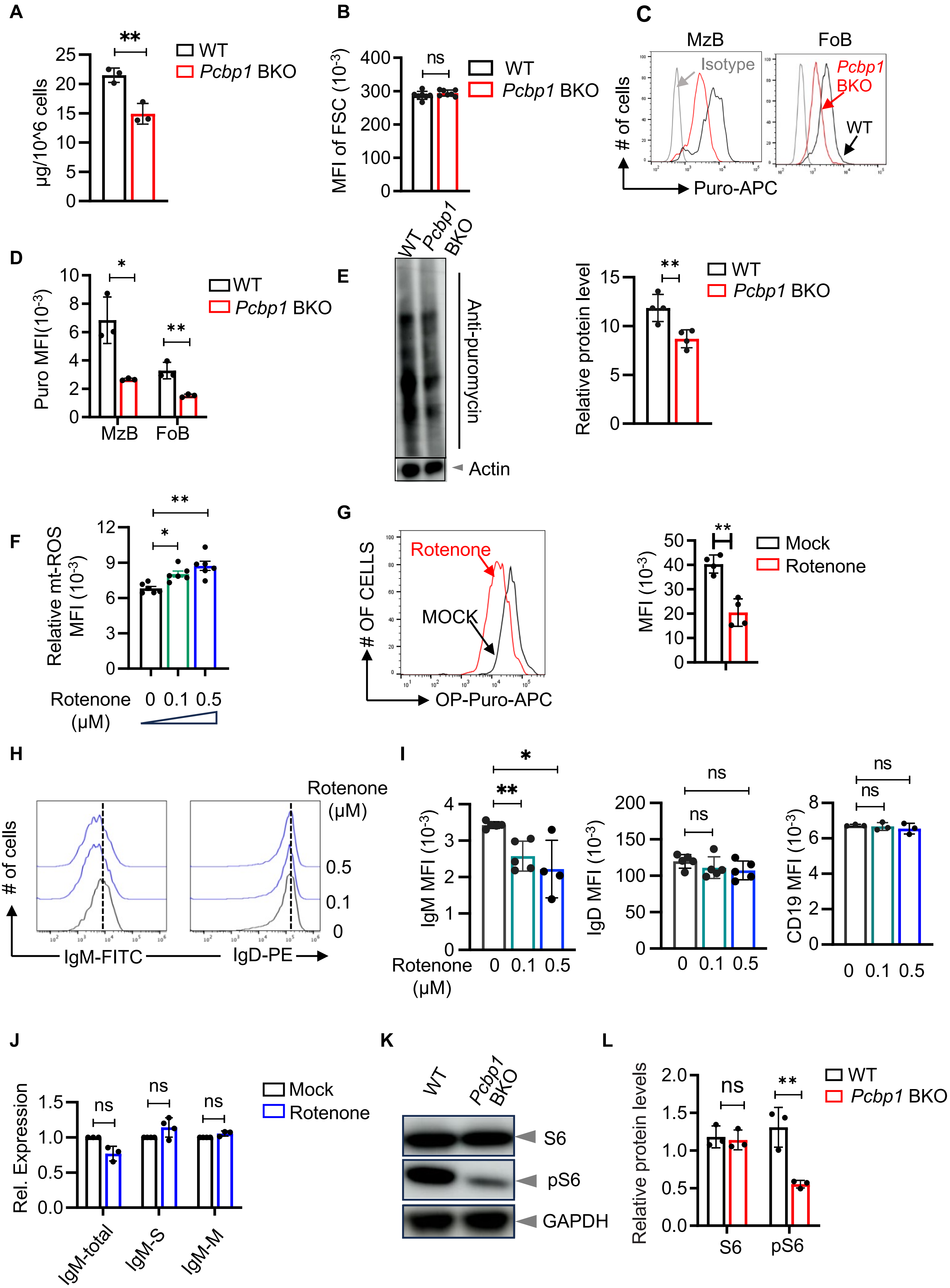
A
**
WT
Pcbp1 BKO
μg/10^6 cells
B
ns
MFI of FSC (10-3)
C
MzB
FoB
Isotype
Pcbp1 BKO
WT
# of cells
Puro-APC
D
Puro MFI(10-3)
MzB
FoB
E
WT
Pcbp1 BKO
Anti-puromycin
Actin
Relative protein level
F
Relative mt-ROS MFI (10-3)
Rotenone (μM)
0 0.1 0.5
G
Rotenone
MOCK
OF CELLS
OP-Puro-APC
MFI (10-3)
Mock
Rotenone
H
Rotenone (μM)
0.5
0.1
0
# of cells
IgM-FITC
IgD-PE
I
IgM MFI (10-3)
IgD MFI (10-3)
CD19 MFI (10-3)
J
Rel. Expression
IgM-total
IgM-S
IgM-M
K
S6
pS6
GAPDH
L
Relative protein levels
S6
pS6

Figure S4. *Pcbp1* promotes IgM translation partially via maintaining mitochondrial complex I

(**A**). Loss of *Pcbp1* decreased total protein content in mature naïve B cells. Protein content from WT and *Pcbp1* BKO naïve B cells from the spleen were determined by a BCA assay and normalized to the number of cells. Data are summaries of three independent experiments. Each dot represents one mouse. **， $p < 0.01$ in Student's *t*-test.

(**B**). B cells from wild-type (WT) and *Pcbp1* B cell-specific knockout (BKO) mice were purified, and cell size was assessed based on the mean fluorescence intensity (MFI) of FSC. Data represent the summary of three independent experiments.

(**C-D**). *Pcbp1* deficiency suppressed global protein translation in marginal zone B (MzB) and follicular B (FoB) cells. Splenocytes were cultured in complete RPMI 1640 medium supplemented with puromycin (10 μg/mL) for 2 hours. The incorporated puromycin in MzB ($CD23^{low}CD21^{high}$) and FoB ($CD23^{high}CD21^{low}$) was detected by flow cytometry using an anti-puromycin-APC antibody. (**C**), Representative FACS plot. (**D**), Puromycin MFI summarized from 2 biological replicates, with each dot representing one mouse. *, $p < 0.05$, **$p < 0.01$ in Student's *t*-test.

(**E**). *Pcbp1* deficiency inhibited global protein translation in LPS stimulated B cells. Naïve B cells from WT or *Pcbp1* BKO mice were stimulated with LPS (20 μg/mL), IL-2 (200 U/mL), and IL-5 (5 ng/mL) for 24 hours and were pulsed with OP-Puro (10 μM) for an additional one hour. OP-Puro incorporation was analyzed by immunoblotting (*left*). Levels of OP-Puro containing proteins were determined using ImageJ after being normalized to Actin (*right*). Data are presented as mean ± SD. Statistical significance was assessed using Student's *t*-test, with **, $p < 0.01$ indicating significance.

(**F**). Rotenone increased mt-ROS in B cells. WT B cells were stimulated with LPS (20 μg/mL), IL-2 (200 U/mL), and IL-5 (5 ng/mL) for 20 hours, followed by treatment with the mitochondrial complex I inhibitor rotenone (0.1 μM or 0.5 μM) for 6 hours. mt-ROS levels were measured via MitoSOX Red superoxide indicator (flow cytometry). Data represent mean ± SD from three independent experiments. Statistical significance was determined using Student's *t*-test. *, $p < 0.05$, **, $p < 0.01$.

(**G**). Rotenone inhibited global protein translation. As in (**F**), LPS stimulated B cells were treated with 0.5 μM rotenone for six hours, then pulsed with OP-Puro for one hour. *Left*, representative flow cytometry plot; *Right*, quantification of OP-Puro incorporation levels. Data represent mean ± SD from three independent experiments. Statistical significance was determined using Student's *t*-test. **, $p < 0.01$.

(**H-I**). Rotenone diminished IgM protein levels. As in (**F**), LPS-activated B cells were treated with 0.1 μM or 0.5 μM rotenone for six hours, and IgM, IgD and CD19 expression was determined by flow cytometry. (**H**). Representative flow cytometric analysis of surface levels of IgM, IgD and CD19. (**I**). Summary of IgM, IgD and CD19 levels from three independent experiments. Statistical significance was determined using Student's *t*-test. *, $p < 0.05$, **, $p < 0.01$.

(**J**). Rotenone treatment had no effect on IgM mRNA levels. As in (**F**), LPS-activated B cells were stimulated with 0.5 μM rotenone for six hours, and levels of total IgM, secreted IgM (IgM-S), and membrane-bound IgM (IgM-M) transcripts were determined by real-time PCR. Data are summarized from three independent experiments.

(**K-L**). *Pcbp1* enhanced mTORC1 activity in naïve B cells. Naïve B cells were isolated from the spleen of WT

and *Pcbp1* BKO mice. Levels of p-S6 and S6 were determined via immunoblotting. Data are representative (**K**) or summary (**L**) of three independent experiments. **, $p < 0.01$ in Student's *t*-test.

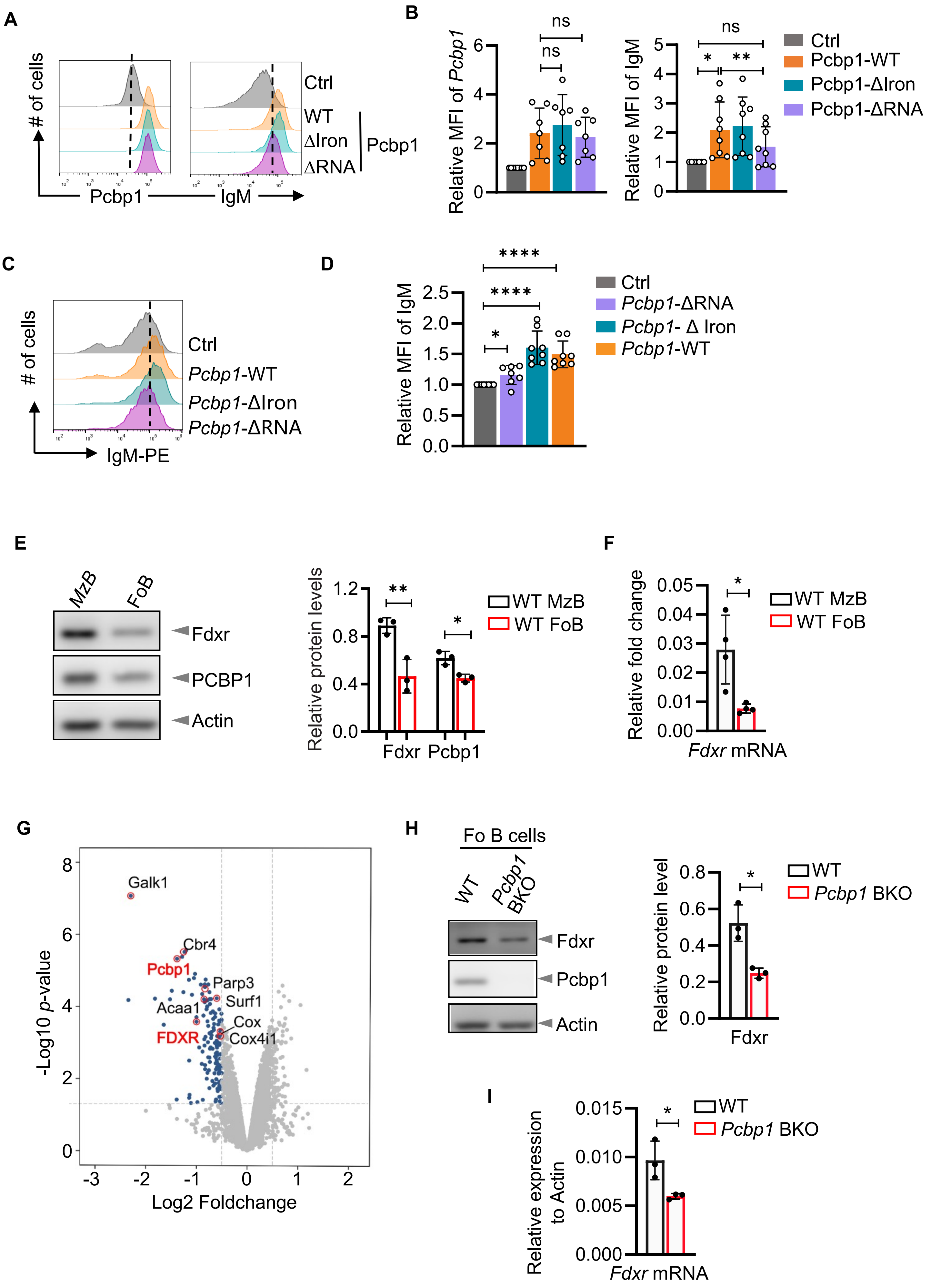
A
# of cells
Ctrl
WT
ΔIron
ΔRNA
Pcbp1
Pcbp1
IgM
B
Relative MFI of Pcbp1
Relative MFI of IgM
ns
*
**
Ctrl
Pcbp1-WT
Pcbp1-ΔIron
Pcbp1-ΔRNA
C
# of cells
Ctrl
Pcbp1-WT
Pcbp1-ΔIron
Pcbp1-ΔRNA
IgM-PE
D
Relative MFI of IgM
****
*
Ctrl
Pcbp1-ΔRNA
Pcbp1- Δ Iron
Pcbp1-WT
E
MzB
FoB
Fdxr
PCBP1
Actin
Relative protein levels
WT MzB
WT FoB
Fdxr
Pcbp1
F
Relative fold change
WT MzB
WT FoB
Fdxr mRNA
G
Galk1
Cbr4
Pcbp1
Parp3
Surf1
Acaa1
Cox
FDXR
Cox4i1
-Log10 p-value
Log2 Foldchange
H
Fo B cells
WT
Pcbp1 BKO
Fdxr
Pcbp1
Actin
Relative protein level
WT
Pcbp1 BKO
Fdxr
I
Relative expression to Actin
WT
Pcbp1 BKO
Fdxr mRNA

Figure S5. *Pcbp1* regulates *Fdxr* expression to promote IgM production

(**A, B**). *Pcbp1* enhanced IgM expression via its RNA-binding activity. Namalwa B cells were transduced with wild-type *Pcbp1* or *Pcbp1* variants specifically designed to impair RNA binding (ΔRNA) or iron binding capabilities (Δiron) (*38*). After transfection, IgM levels were determined by flow cytometry. (**A**). Representative flow cytometry analysis. (**B**). Summary of *Pcbp1* and IgM expression levels determined by flow cytometry from nine independent experiments. Statistical significance was evaluated using Student's *t*-test. *, $p < 0.05$, **, $p < 0.01$.

(**C, D**). RNA binding activity of *Pcbp1* was critical for promoting IgM expression in primary B cells. Naïve B cells isolated from *Pcbp1* BKO mice were stimulated with LPS (20 µg/mL), IL-2 (200 U/mL), and IL-5 (5 ng/mL) for 24 hours. Cells were subsequently transduced with retrovirus expressing either wild-type *Pcbp1* or its variants. Following 24-hour puromycin selection (3 µg/mL), surface IgM levels were quantified by flow cytometry at day 3 post-stimulation. Data are representative (**C**) and a summary (**D**) from two independent experiments (n=5). *, $p<0.05$, ****, $p < 0.0001$ in Student's *t* test.

(**E, F). *Fdxr*** and *Pcbp1* exhibit higher expression levels in marginal zone B cells compared to follicular B cells. (**E**). Marginal zone B cells (MzB; $CD21^{+}CD23^{-}$) and follicular B cells (FoB; $CD21^{int}CD23^{hi}$) were sorted from 6–8-week-old WT mice. Protein levels of *Fdxr* and *Pcbp1* were analyzed by immunoblotting (*left*) and quantified using ImageJ after normalization to actin (*right*).

(**F**). *Fdxr* transcripts were determined by real-time PCR. Data are presented as mean ± SD from three independent experiments. Statistical significance was assessed using Student's *t*-test (*, $p < 0.05$).

(**G**). Volcano plot depicting proteomic analysis of total cellular proteins extracted from naïve B cells of seven-week-old *Pcbp1* BKO mice or WT littermates. Differentially expressed proteins were identified using criteria of $|log2FC| >= 0.5$ and $p < 0.05$.

(**H, I**). Loss of *Pcbp1* reduced *Fdxr* level in FoB cells**.** WT and *Pcbp1* BKO mice were depleted with marginal zone B (MZB) cells. (**H**). Protein levels of *Fdxr* were analyzed by immunoblotting (*left*) and quantified using ImageJ after normalization to actin (*right*). (**I**). *Fdxr* transcripts were determined by real-time PCR. Data are presented as mean ± SD from three independent replicates. *, $p < 0.05$ in Student's *t*-test.

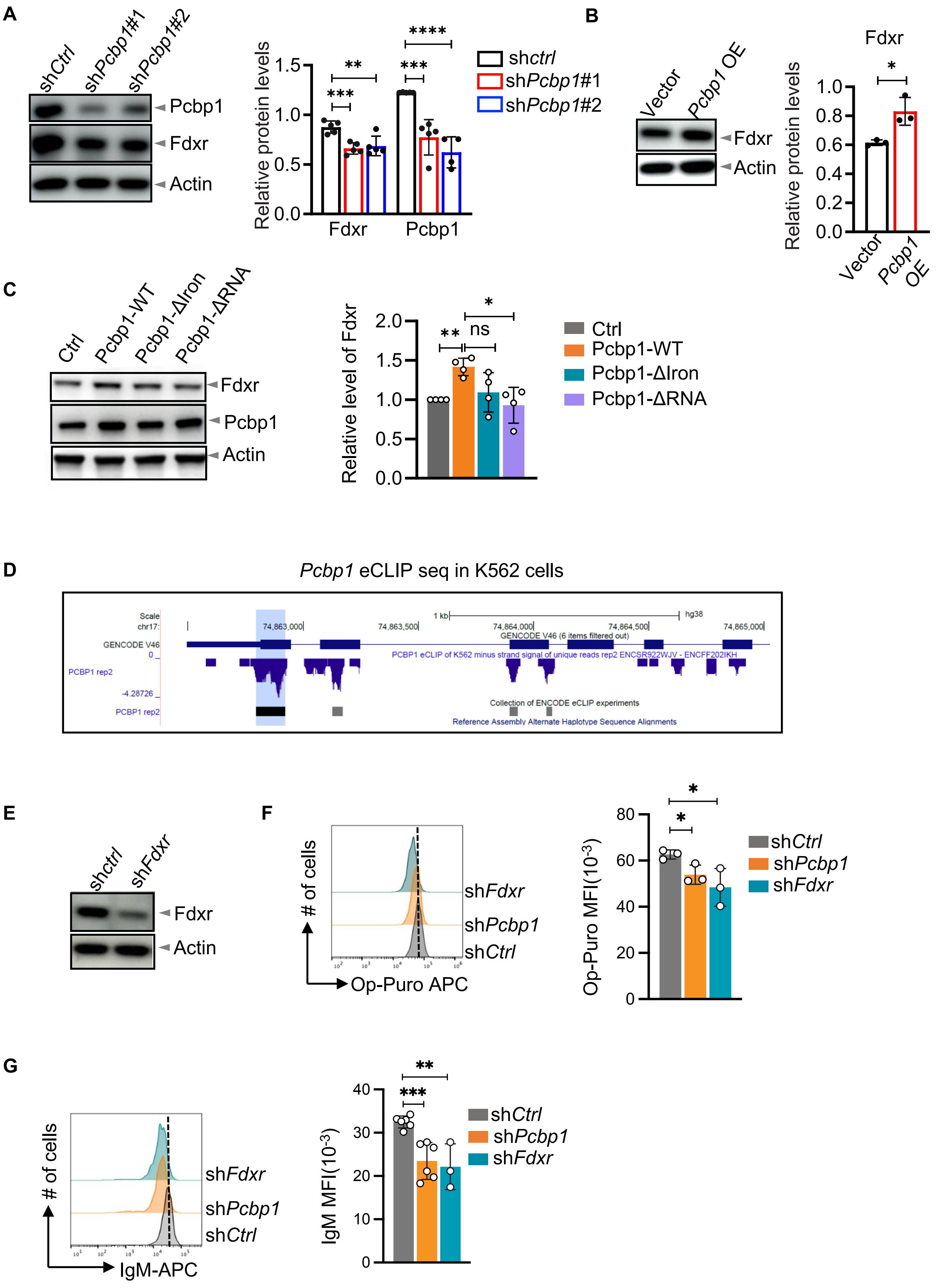
A
shCtrl
shPcbp1#1
shPcbp1#2
Pcbp1
Fdxr
Actin
Relative protein levels
Fdxr
Pcbp1
shctrl
shPcbp1#1
shPcbp1#2
B
Vector
Pcbp1 OE
Fdxr
Actin
Relative protein levels
C
Ctrl
Pcbp1-WT
Pcbp1-ΔIron
Pcbp1-ΔRNA
Fdxr
Pcbp1
Actin
Relative level of Fdxr
ns
D
Pcbp1 eCLIP seq in K562 cells
Scale
chr17:
GENCODE V46
PCBP1 rep2
hg38
GENCODE V46 (6 items filtered out)
PCBP1 eCLIP of K562 minus strand signal of unique reads rep2 ENCSR922WJV - ENCFF202IKH
Collection of ENCODE eCLIP experiments
Reference Assembly Alternate Haplotype Sequence Alignments
E
shctrl
shFdxr
Fdxr
Actin
F
# of cells
shFdxr
shPcbp1
shCtrl
Op-Puro APC
Op-Puro MFI(10^-3)
shCtrl
shPcbp1
shFdxr
G
# of cells
shFdxr
shPcbp1
shCtrl
IgM-APC
IgM MFI(10^-3)
shCtrl
shPcbp1
shFdxr

Figure S6. *Pcbp1* promotes *Fdxr* expression through its RNA-binding activity in B cells

(**A**). *Pcbp1* enhanced *Fdxr* expression in B cells. Namalwa B cells were retrovirally transduced two independent shRNAs against Pcbp1, and Fdxr expression levels were determined via immunoblotting (left) and quantified using ImageJ after normalization to actin (right). Data are presented as mean ± SD from five independent experiments. **, $p < 0.01$, ***, $p<0.001$, ****, $p < 0.0001$ in Student's *t*-test.

(**B**). Namalwa B cells were transduced with retrovirus containing *Pcbp1*, and *Fdxr* expression levels were determined via immunoblotting. Data are representative of three independent experiments. Statistical significance was assessed using Student's *t*-test. *, $p < 0.05$.

(**C**). Namalwa B cells were transduced with retroviruses encoding WT-*Pcbp1*, *Pcbp1* variants specifically designed to disrupt RNA binding (ΔRNA) or iron binding (Δiron), and *Fdxr* expression was assessed by immunoblotting (*left*) and quantified with ImageJ after normalization to actin (*right*). Data are presented as mean ± SD from five independent experiments. *, $p < 0.05$, **, $p < 0.01$ in Student's *t*-test.

(**D**). *Pcbp1* bound to the last exon of *Fdxr* mRNA. Data are derived from e-CLIP results of K562 cells (*59*).

(**E**). Namalwa B cells were transduced with lentivirus containing shRNA targeting *Fdxr*. Efficiency of *Fdxr* knockdown was assessed by immunoblotting.

(**F**). Deletion of *Fdxr* inhibited global protein translation. Namalwa B cells with *Fdxr* knockdown, as described in (**E**), were pulsed with 2 μM OP-Puro for three hours, and OP-puro incorporation was determined by intracellular staining. Namalwa B cells with *Pcbp1* knockdown were included as positive controls. *Left*, representative flow cytometry data; *Right*, summary of OP-Puro incorporation levels from three independent experiments. *, $p < 0.05$ in Student's *t*-test.

(**G**). *Fdxr* enhanced IgM expression in Namalwa B cells. As in (**E**), IgM expression in the *Fdxr* deficient Namalwa B cells were analyzed by flow cytometry. Namalwa B cells with Pcbp1 knockdown were included as positive controls. Shown are representative (*left*) or summary (*right*) of three independent experiments. **, $p < 0.01$, ***, $p<0.001$ in Student's t-test.

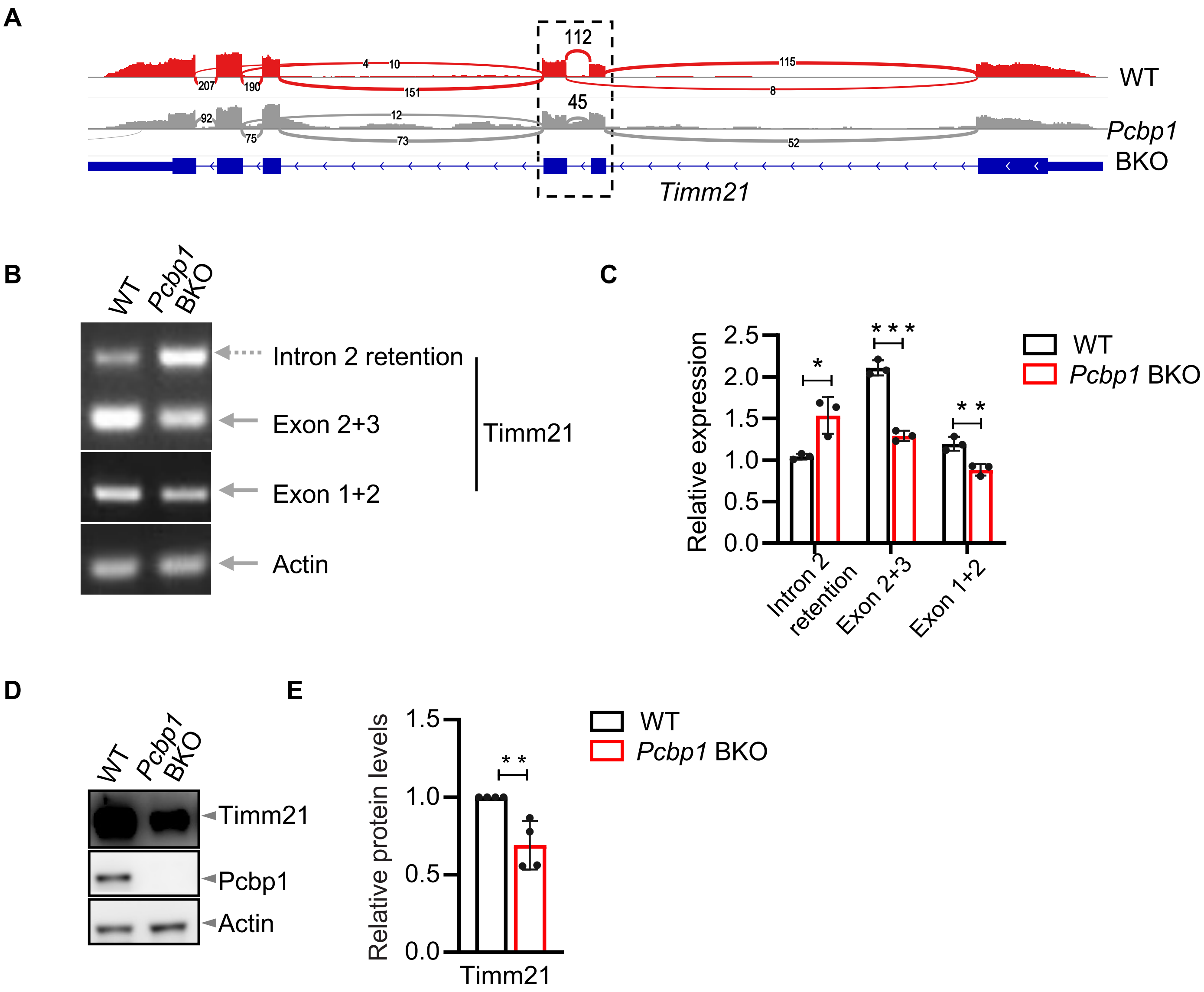
A
112
4
10
115
207
190
151
8
WT
45
92
12
75
73
52
Pcbp1
BKO
Timm21
B
WT
Pcbp1 BKO
Intron 2 retention
Exon 2+3
Exon 1+2
Actin
Timm21
C
Relative expression
2.5
2.0
1.5
1.0
0.5
0.0
*
* * *
* *
Intron 2 retention
Exon 2+3
Exon 1+2
WT
Pcbp1 BKO
D
WT
Pcbp1 BKO
Timm21
Pcbp1
Actin
E
Relative protein levels
1.5
1.0
0.5
0.0
* *
Timm21
WT
Pcbp1 BKO

Figure S7. *Pcbp1* modulates 2nd intron retention of *Timm21* in B cells

Naïve B cells from WT and *Pcbp1* BKO mice were stimulated with LPS (20 μg/mL), IL-2 (200 U/mL), and IL-5 (5 ng/mL) for three days, the alternative splicing of *Timm21* was analyzed.

(**A**). *Pcbp1* deficiency led to intron retention of *Timm21*. RNAseq analysis was performed as previously described, Sashimi plots indicating the RNA-Seq read counts and estimated intron retention levels for *Timm21* in B cells from WT and *Pcbp1* BKO. Data were representative of three independent experiments.

(**B**). Semi-quantitative RT-PCR analysis of RNA-seq predicted isoform expression in WT and *Pcbp1* BKO group for *Timm21* after 3 days of LPS stimulation, with Actin as a loading control, the dashed arrows indicate the intron 2 retention isoform.

(**C**). Statistical analysis of semi-quantitative RT-PCR. Data are from 3 biological replicates. *, $p < 0.05$, **, $p<0.01$, ***, $p<0.001$ in Student's *t*-test.

(**D, E**). Loss of *Pcbp1* impaired *Timm21* expression. As in (**B**), The protein levels were determined by western blotting (**D**, representative) and further analyzed using ImageJ after being normalized to Actin (**E**). Data are presented as mean ± SD. **, $p < 0.01$ in Student's *t* test.

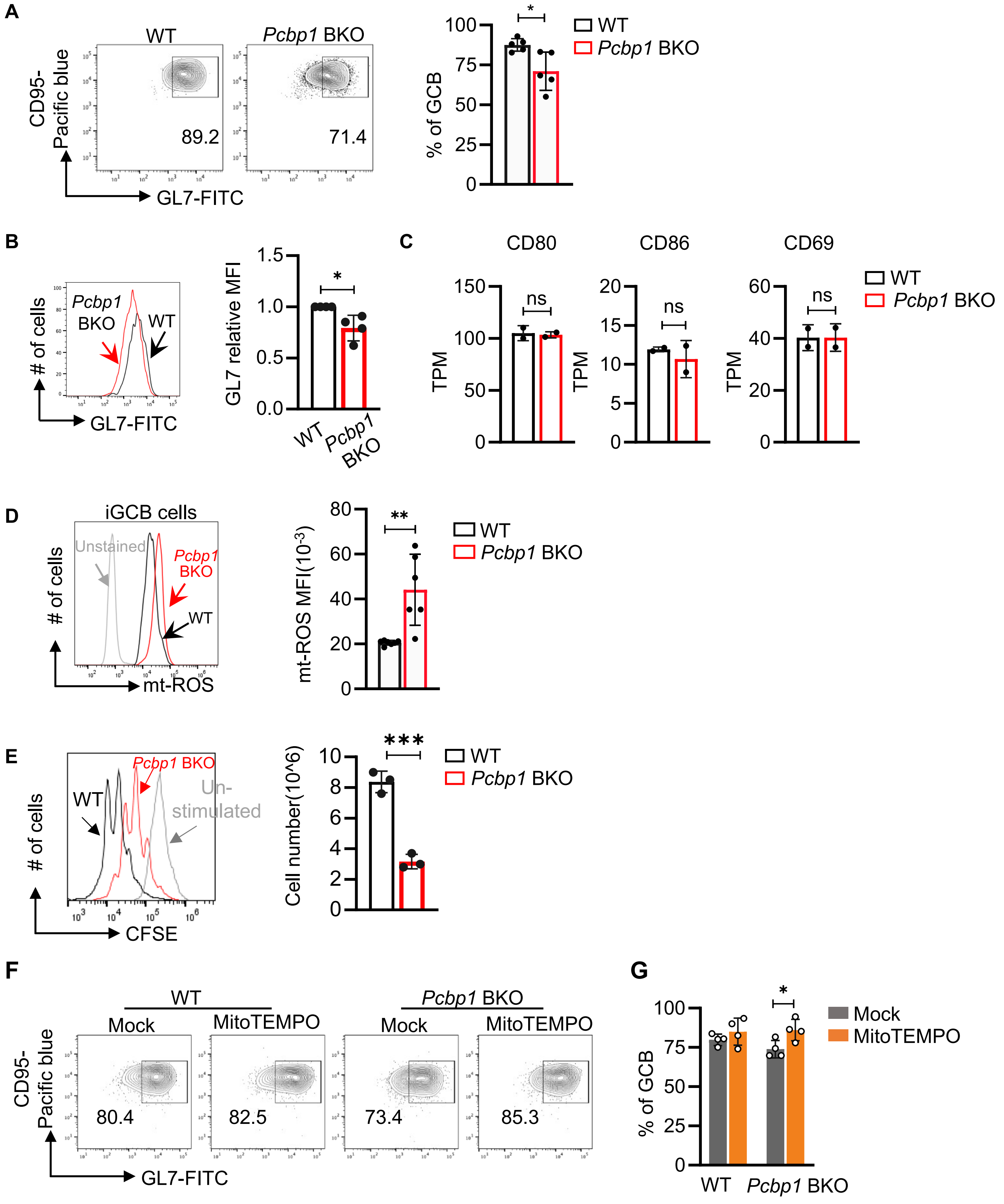
A
WT
Pcbp1 BKO
CD95-Pacific blue
GL7-FITC
89.2
71.4
% of GCB
WT
Pcbp1 BKO
B
# of cells
Pcbp1 BKO
WT
GL7-FITC
GL7 relative MFI
C
CD80
CD86
CD69
TPM
ns
D
iGCB cells
Unstained
# of cells
mt-ROS
mt-ROS MFI(10^-3)
E
WT
Pcbp1 BKO
Un-stimulated
# of cells
CFSE
Cell number(10^6)
F
WT
Pcbp1 BKO
Mock
MitoTEMPO
80.4
82.5
73.4
85.3
CD95-Pacific blue
GL7-FITC
G
% of GCB
Mock
MitoTEMPO

Figure S8. *Pcbp1* regulates GCB development by modulating mitochondrial function *in vitro*

(**A**). *Pcbp1* deficiency compromised differentiation of iGCB cells *in vitro.* Naïve B cells from WT or *Pcbp1* B KO mice were cultured under iGCB condition for three days, and GCB percentage was determined by GL7 and CD95 staining. The data are representative (*left*) or summary (*right*) of three independent experiments. *, $p<0.05$, in Student's t test.

(**B**). Pcbp1 deficiency impairs GL7 expression in iGCB cells. Naïve B cells from WT or *Pcbp1* BKO mice were cultured under iGCB conditions for three days, and GL7 expression was assessed by flow cytometry. Data are shown as representative histograms (*left*) and summarized results (*right*) from three independent experiments. *, $p < 0.05$, Student's *t*-test.

(**C**). Loss of *Pcbp1* did not affect expression of genes associated with immediate B cell activation. Naïve B cells from WT or *Pcbp1* BKO mice were cultured under *in vitro* GCB differentiation conditions for three days and analyzed by bulk RNA-seq. Expression of CD80, CD86, and CD69 was quantified as TPM.

(**D**). Loss of *Pcbp1* increased mt-ROS level in iGCB cell. As in (**A**), Naïve B cells from WT or *Pcbp1* B KO mice were cultured under iGCB condition for four days, mt-ROS levels were measured via MitoSOX Red superoxide indicator. Data represent mean ± SD from three independent experiments. Statistical significance was determined using Student's *t*-test (**, $p < 0.01$).

(**E**). *Pcbp1* deficiency compromised proliferation and expansion of iGCB cells. Induced germinal center B (iGCB) cells from *Pcbp1* BKO and WT control mice were differentiated in the 40LB system for 72 hours. Cell division was assessed by CFSE dilution using flow cytometry. *Left*, representative histograms of cell division generations. *Right*, cell counts were quantified starting from an initial 10^6 cells after 72 hours. Data are presented as mean ± SD from three independent experiments (n=3). ***, $p<0.001$, Student's *t*-test.

(**F-G**). MitoTEMPO rescued the defects of iGCB differentiation in *Pcbp1*-deficient B cells. Naïve B cells isolated from WT or *Pcbp1* BKO mice were stimulated under germinal center B (GC B) conditions in the presence of 50 μM MitoTEMPO for 72 hours. The percentage of iGCB cells ($CD95^+GL7^+$) was determined via flow cytometry. Data are representative (**F**) or summary (**G**) of four independent experiments. Student's *t*-test, *, $p < 0.05$.

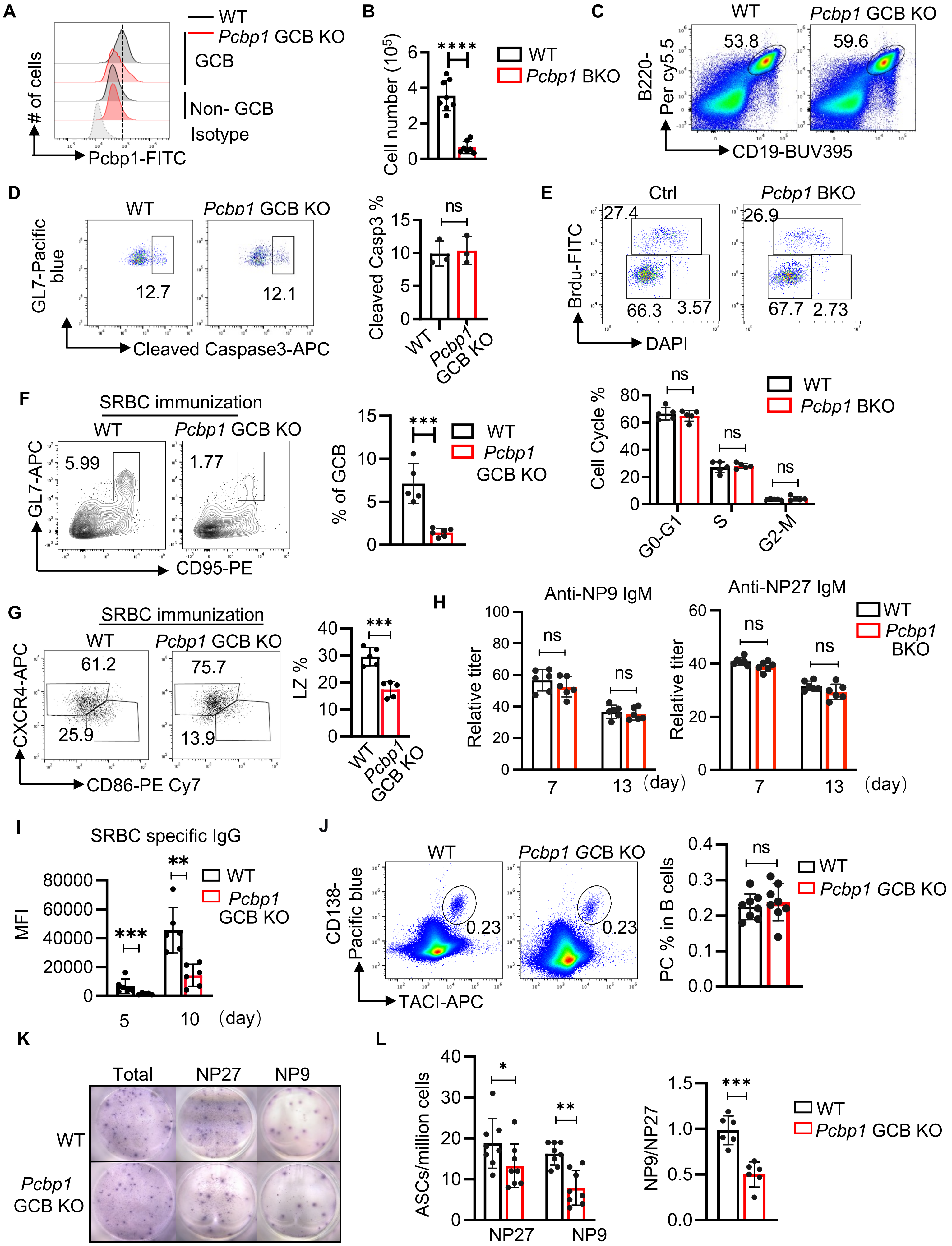
A
WT
Pcbp1 GCB KO
GCB
Non- GCB
Isotype
# of cells
Pcbp1-FITC
B
****
WT
Pcbp1 BKO
Cell number (10^5)
C
WT
Pcbp1 GCB KO
53.8
59.6
B220-Per cy5.5
CD19-BUV395
D
WT
Pcbp1 GCB KO
GL7-Pacific blue
12.7
12.1
Cleaved Caspase3-APC
Cleaved Casp3 %
ns
E
Ctrl
Pcbp1 BKO
27.4
26.9
66.3
3.57
67.7
2.73
Brdu-FITC
DAPI
Cell Cycle %
G0-G1
S
G2-M
F
SRBC immunization
5.99
1.77
GL7-APC
CD95-PE
% of GCB
***
G
SRBC immunization
61.2
75.7
25.9
13.9
CXCR4-APC
CD86-PE Cy7
LZ %
H
Anti-NP9 IgM
Anti-NP27 IgM
Relative titer
(day)
I
SRBC specific IgG
MFI
**
J
CD138-Pacific blue
TACI-APC
0.23
PC % in B cells
K
Total
NP27
NP9
L
ASCs/million cells
NP9/NP27
*

Figure S9. *Pcbp1* deficiency impairs germinal center B cell responses *in vivo*

(**A**). Specific depletion of *Pcbp1* in germinal center B cells of $AID^{Cre/+}Pcbp1^{fl/fl}$ (*Pcbp1* GCB KO) mice. 13 days following NP-KLH immunization, expression of *Pcbp1* in GCB cells ($CD19^+B220^+GL7^+CD95^+$) and non-GC B cells ($CD19^+B220^+GL7^-CD95^-$) was detected via intracellular staining. Data are representative of five independent experiments.

(**B**). Total number of germinal center B (GCB) cells in WT or *Pcbp1* GCB KO mice. Data are summary of three independent experiments, ****, $p < 0.0001$ in Student's *t* test.

(**C**). Percentage of B cells in unimmunized *Pcbp1* GCB KO mice, determined by CD19 and B220 staining via flow cytometry. Data are representative of three independent experiments.

(**D**). *Pcbp1* deficiency did not affect GCB cell apoptosis. 13 days post NP-KLH immunization, splenic GCB cells ($CD19^+B220^+FAS^+GL7^+$) were identified by flow cytometry, apoptosis GCB cells were assessed by intracellular cleaved Caspase-3 staining. *Left*, representative flow cytometry plot. *Right*, summary of 3 independent experiments.

(**E**). *Pcbp1* deficiency did not affect GCB cell cycle progression. 5 days post SRBC immunization, mice were intraperitoneally injected with 1 mg of BrdU, and were euthanized 40 minutes later. Splenic GCB cells were stained for BrdU (S phase) and DAPI (G0/G1 and M phases) and analyzed by flow cytometry. *Left*, representative flow cytometry plot. *Right*, summary of 2 independent experiments (n=5 mice).

(**F**). WT or *Pcbp1* GCB KO mice were analyzed for germinal center B cells ten days post SRBC immunization. *Top*, representative staining. *Bottom*, summary of percentages of GCB in total B cells from 3 independent experiments (n=6). ***, $p<0.001$ in Student's *t* test.

(**G**). *Pcbp1* deficiency in GC B cells impaired the differentiation of light zone B cells following SRBC immunization. *Left*, representative staining. *Right*, percentages of light zone B cells within the total GCB cells were summarized from 3 independent experiments (n=6). ***, $p<0.001$, Student's *t*-test.

(**H**). *Pcbp1* deficiency in GC B cells did not affect IgM response following NP-KLH immunization. WT or *Pcbp1* GCB KO mice were immunized with NP-KLH in Alum, and serum levels of high-affinity anti-NP IgM (NP9) (*left*) and low-affinity anti-NP IgM (NP27) (*right*) were measured by ELISA at day 7 and day 13 post-immunization. Data represents results from at least 3 independent experiments.

(**I**). Reduced serum anti-SRBC IgG levels in *Pcbp1* GCB KO mice post-SRBC immunization. As in (**F**), levels of anti-SRBC IgG in serum were determined by flow cytometry as described (*60*). Statistical significance was determined using Student's *t*-test, with significance indicated as **, $p < 0.01$, ***, $p<0.001$.

(**J**). *Pcbp1* deficiency had no effect on total plasma B cell following NP-KLH immunization. AID Cre/+*Pcbp1 fl/fl* (*Pcbp1* GCB KO) and WT mice were immunized with NP-KLH and Alum. Mice were analyzed 13-days post-immunization. *Left*, representative flow cytometry plots of plasma cells ($TACI^+CD138^+$). *Right*, quantification of plasma cell frequencies in the spleen.

(**K-L**). Quantification of bone marrow antibody-secreting cells (ASCs) with high-affinity (NP9) and low-affinity (NP27) anti-NP IgG in WT and *Pcbp1* GCB KO mice. ELISPOT assays were performed using Millipore MAHA 96-well filter plates coated overnight at 4°C with goat anti-mouse IgG (50 μg/mL), NP27-BSA (50 μg/mL), or NP7-BSA (50 μg/mL) in PBS. Plates were incubated with $1×10^6$ bone marrow cells per well for 5 hours, then

removed by washing with PBS. Plates were incubated overnight at 4°C with ALP-conjugated anti-mouse IgG (0.5 μg/mL), and developed with BCIP/NBT-plus substrate according to the manufacturer's instructions, ASCs were quantified using ImageJ. (**K**). Representative images. (**L**). Summary of anti-NP9 and anti-NP27 ASC counts from eight mice across two independent experiments, with NP9/NP27 ratios comparing WT and *Pcbp1* GCB KO mice, indicating predominant low-affinity ASC production in KO mice. *, $p < 0.05$, **, $p < 0.01$, ***, $p<0.001$ in Student's *t*-test,

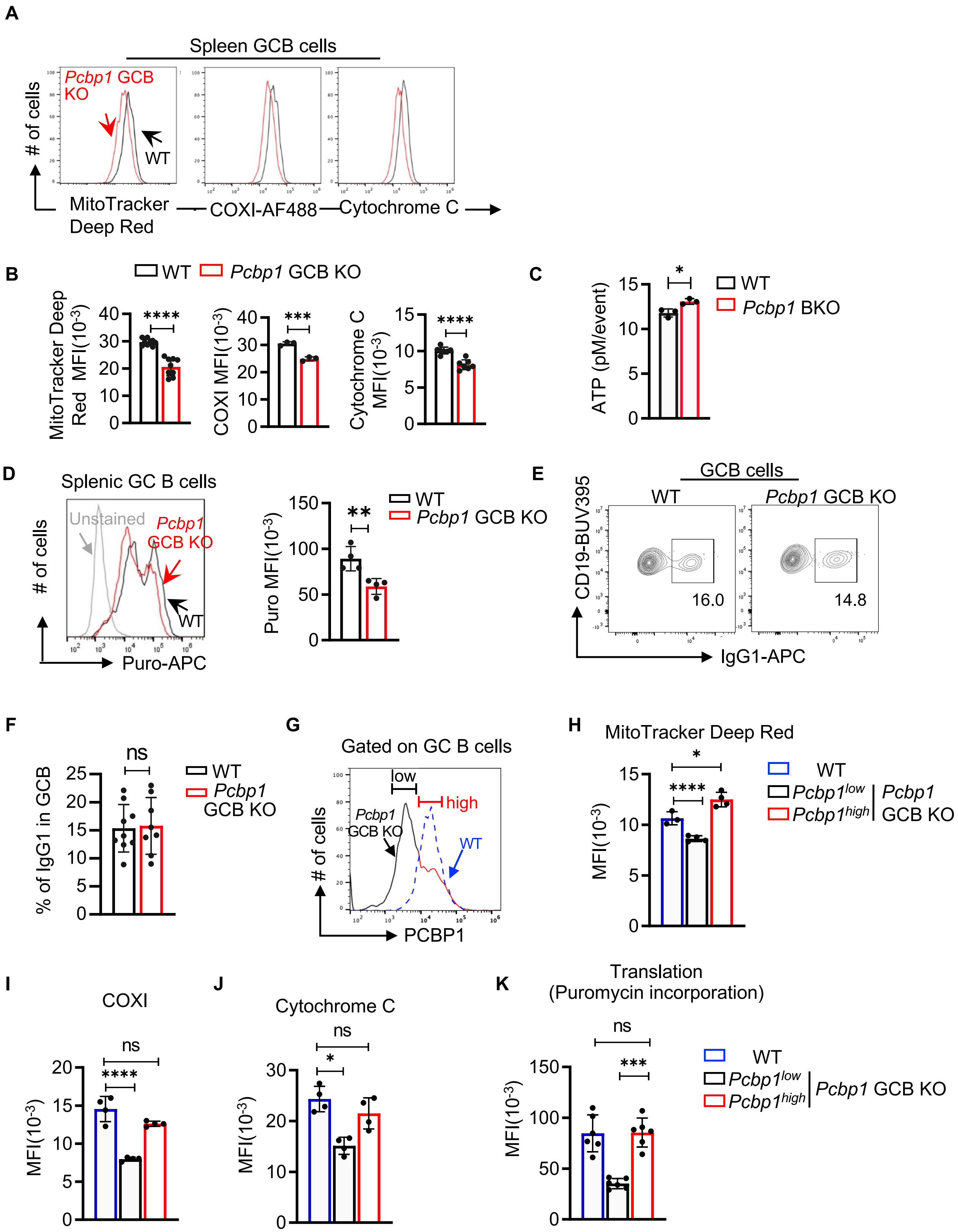
A
Spleen GCB cells
Pcbp1 GCB KO
WT
# of cells
MitoTracker Deep Red
COXI-AF488
Cytochrome C
B
WT
Pcbp1 GCB KO
MitoTracker Deep Red MFI(10^-3)
COXI MFI(10^-3)
Cytochrome C MFI(10^-3)
C
ATP (pM/event)
WT
Pcbp1 BKO
D
Splenic GC B cells
Unstained
Pcbp1 GCB KO
WT
Puro-APC
Puro MFI(10^-3)
E
GCB cells
WT
Pcbp1 GCB KO
CD19-BUV395
16.0
14.8
IgG1-APC
F
% of IgG1 in GCB
ns
WT
Pcbp1 GCB KO
G
Gated on GC B cells
low
high
Pcbp1 GCB KO
WT
PCBP1
H
MitoTracker Deep Red
MFI(10^-3)
WT
Pcbp1 low
Pcbp1 high
Pcbp1 GCB KO
I
COXI
J
Cytochrome C
K
Translation (Puromycin incorporation)
MFI(10^-3)
WT
Pcbp1 low
Pcbp1 high
Pcbp1 GCB KO

Figure S10. *Pcbp1* expression levels influence mitochondrial function and protein synthesis in germinal center B cells

(**A, B**). Loss of *Pcbp1* reduced mitochondrial mass and electron transport chain activity in germinal center B cells. WT and *Pcbp1* GCB KO mice were immunized with NP-KLH, 13 days later, mitochondrial mass and mitochondrial complex IV subunits (MT-CO1 and Cytochrome C) were assessed via intracellular staining. (**A**). Representative staining. (**B**). Quantification from three independent experiments. Statistical significance was determined using Student's *t*-test (***, $p<0.001$, ****, $p<0.0001$).

(**C**). *Pcbp1* deficiency enhanced ATP production in germinal center B (GCB) cells. GCB cells ($CD19^{+}GL7^{+}CD95^{+}$) were sorted from spleens of WT and *Pcbp1* GCB KO mice 13 days post-immunization, and intracellular ATP levels were measured using a luminescent ATP detection assay kit. Data are summarized from three independent experiments. Statistical significance was assessed using Student's *t*-test (*, $p < 0.05$).

(**D**). *Pcbp1* deficiency impaired global protein translation in GC B Cells. As in (**A**), protein synthesis in germinal center B (GCB) cells was assessed by puromycin incorporation. Splenocytes were cultured with puromycin (10 μg/mL) for 60 minutes, and puromycin incorporation in GC B cells was analyzed by flow cytometry. *Left*, representative flow cytometry staining. *Right*, quantification from three independent experiments. Data are presented as mean ± SD. Statistical significance was determined using Student's *t*-test (**, $p < 0.01$).

(**E, F**). Expression of IgG1 was determined in GCB cells via intracellular staining. Data are representative (**E**) and summary (**F**) from three independent experiments. Error bars represent mean ± SD.

(**G-K**). *Pcbp1* regulated mitochondrial homeostasis and translation efficiency in germinal center B cells through cell-autonomous mechanisms. WT and *Pcbp1* GCB KO mice were immunized with NP-KLH for 13 days, and germinal center B cells from *Pcbp1* GCB KO mice were stratified into $Pcbp1^{high}$ and $Pcbp1^{low}$ subpopulations.

(**G**). Flow cytometry analysis of *Pcbp1* expression in GC B cells: wild-type (WT, blue dashed line) versus *Pcbp1* knockout (GCB KO) cells with low ($Pcbp1^{low}$, black solid line) and high ($Pcbp1^{high}$, red solid line) expression levels.

(**H-K**). As in (**G**), quantification of mitochondrial parameters and protein synthesis across different subsets (WT GCB, blue; $Pcbp1^{low}$ GCB, black; $Pcbp1^{high}$ GCB, red). (**H**). MitoTracker Deep Red staining indicating mitochondrial mass/function. (**I**). COXI expression analysis. (**J**). Cytochrome C levels. (**K**). Puromycin incorporation assay measuring protein synthesis**.** Data represent mean ± SD from independent experiments, with individual data points shown. *, $p<0.05$, ***, $p<0.001$, ****, $p<0.0001$ in Student's *t*-test.

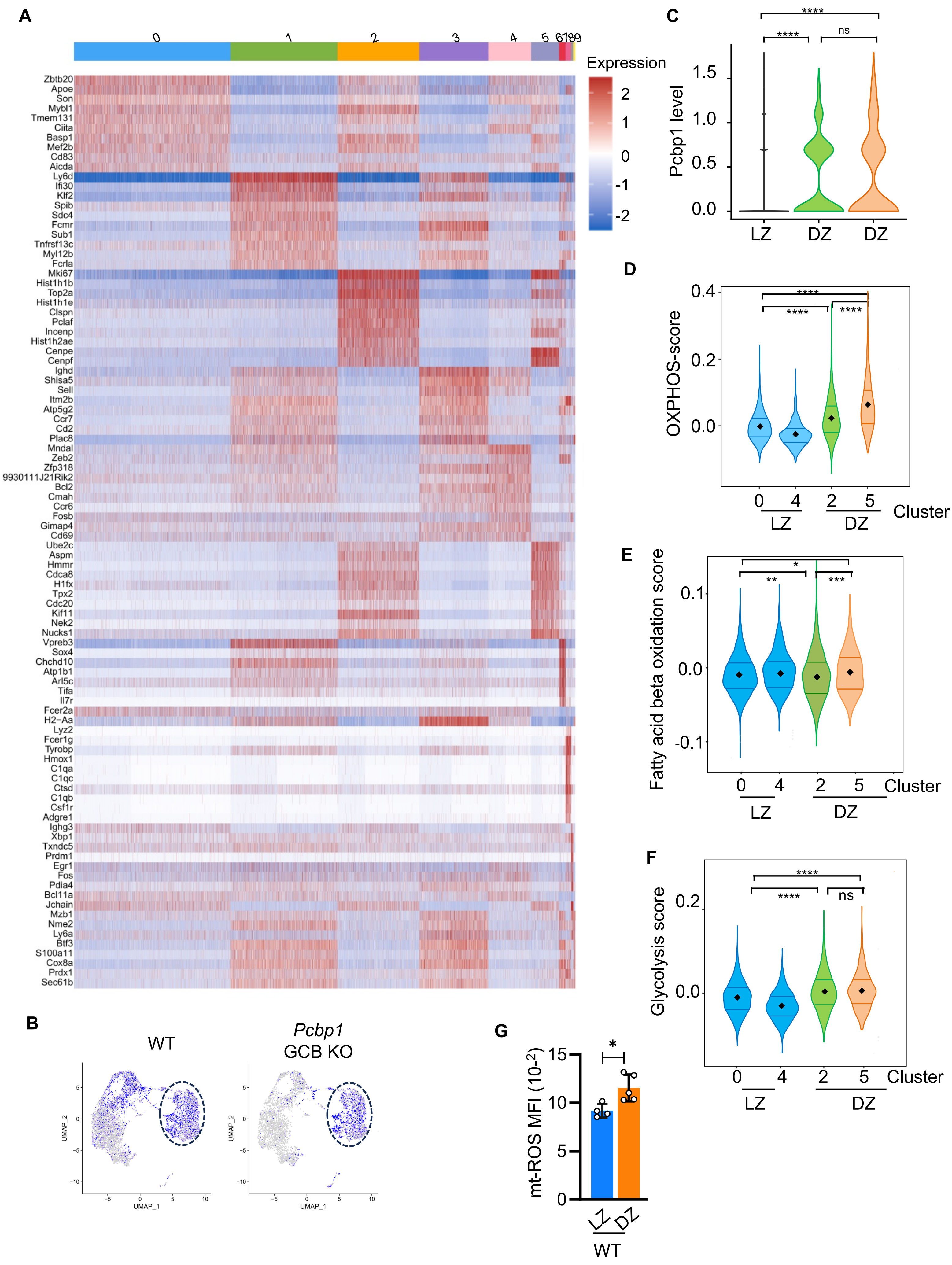
A
0
1
2
3
4
5
6789
Expression
2
1
0
-1
-2
Zbtb20
Apoe
Son
Mybl1
Tmem131
Ciita
Basp1
Mef2b
Cd83
Aicda
Ly6d
Ifi30
Klf2
Spib
Sdc4
Fcmr
Sub1
Tnfrsf13c
Myl12b
Fcrla
Mki67
Hist1h1b
Top2a
Hist1h1e
Clspn
Pclaf
Incenp
Hist1h2ae
Cenpe
Cenpf
Ighd
Shisa5
Sell
Itm2b
Atp5g2
Ccr7
Cd2
Plac8
Mndal
Zeb2
Zfp318
9930111J21Rik2
Bcl2
Cmah
Ccr6
Fosb
Gimap4
Cd69
Ube2c
Aspm
Hmmr
Cdca8
H1fx
Tpx2
Cdc20
Kif11
Nek2
Nucks1
Vpreb3
Sox4
Chchd10
Atp1b1
Arl5c
Tifa
Il7r
Fcer2a
H2−Aa
Lyz2
Fcer1g
Tyrobp
Hmox1
C1qa
C1qc
Ctsd
C1qb
Csf1r
Adgre1
Ighg3
Xbp1
Txndc5
Prdm1
Egr1
Fos
Pdia4
Bcl11a
Jchain
Mzb1
Nme2
Ly6a
Btf3
S100a11
Cox8a
Prdx1
Sec61b
B
WT
Pcbp1
GCB KO
UMAP_1
UMAP_2
C
Pcbp1 level
****
****
ns
1.5
1.0
0.5
0.0
LZ
DZ
DZ
D
OXPHOS-score
****
****
****
0.4
0.2
0.0
0
4
2
5
Cluster
LZ
DZ
E
Fatty acid beta oxidation score
*
**
***
0.1
0.0
-0.1
0
4
2
5
Cluster
LZ
DZ
F
Glycolysis score
****
****
ns
0.2
0.0
0
4
2
5
Cluster
LZ
DZ
G
mt-ROS MFI (10-2)
*
15
10
5
0
LZ
DZ
WT

Figure S11. Single cell RNA analysis of WT and *Pcbp1*-deficient GCB cells

(**A**). Heatmap showing relative expression (z-score of log2(counts per million (CPM) + 1)) of top ten marker genes (rows) for each designated cluster (columns) in Figure **6A**.

(**B**). UMAP visualization of single-cell RNA sequencing data from wild-type (WT, *left*) and *Pcbp1* GCB KO mice (*right*) with color intensity indicating *Pcbp1* expression levels.

(**C**). Expression of *Pcbp1* in light zone and dark zone B cells determined by scRNA-seq. *Pcbp1* expression in light zone B cells (clusters 0 and 4) and dark zone B cells (clusters 2 and 5) from WT mice was assessed by scRNA-seq.

(**D-F**). Violin plots showing the distribution of metabolism-related signature scores in light zone (LZ; clusters 0 and 4) and dark zone (DZ; clusters 2 and 5) B cell clusters, based on scRNA-seq data from WT mice.

(**G**). Elevated mitochondrial reactive oxygen species (mt-ROS) levels were observed in the dark zone compared to the light zone of germinal centers. mt-ROS was quantified using the MitoSOX™ Red superoxide indicator in histologically defined light and dark zone regions. Data are shown as mean ± SD from three independent experiments. Statistical analysis was performed using Student's *t*-test (*, $p < 0.05$).

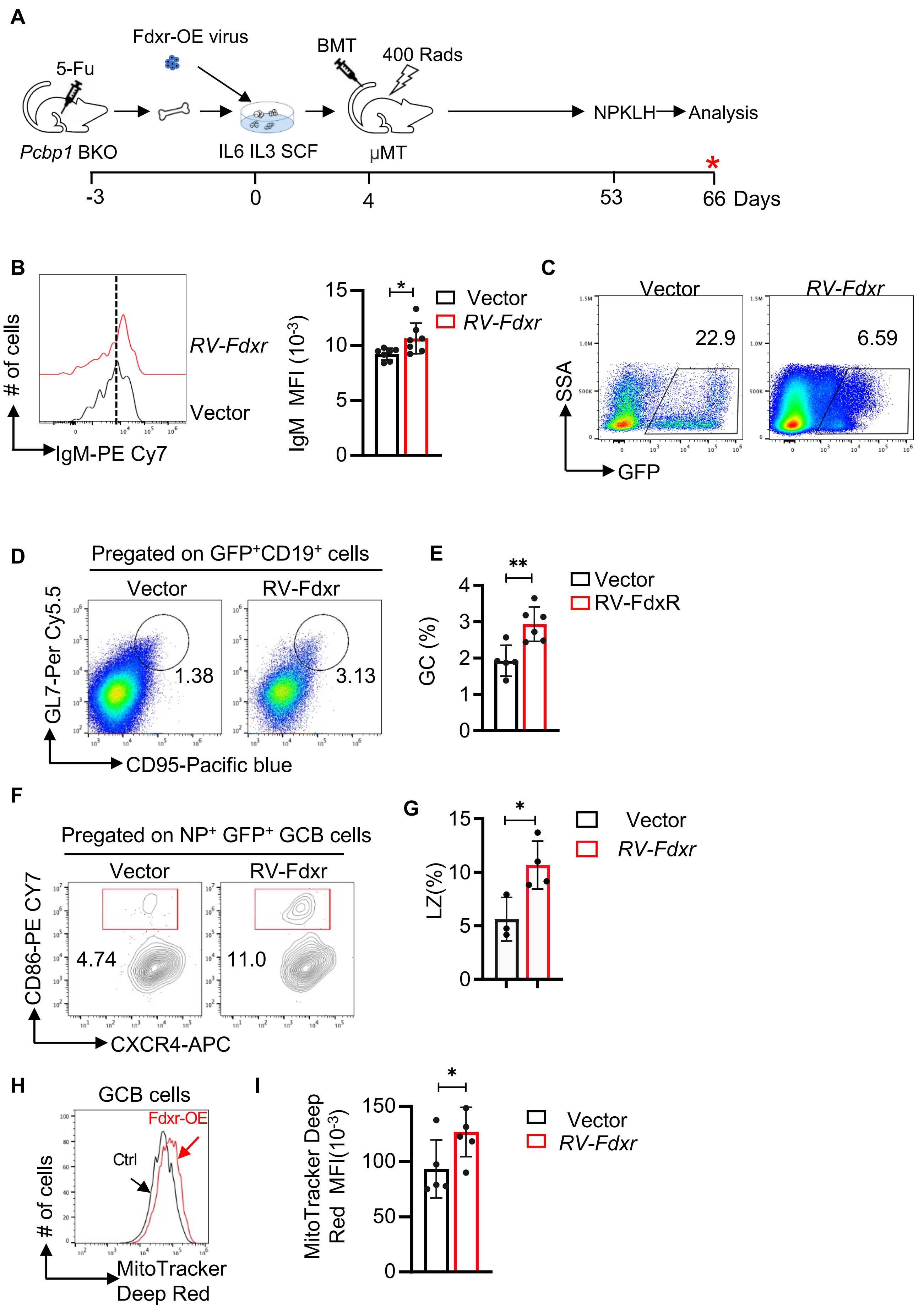
A
Fdxr-OE virus
5-Fu
BMT
400 Rads
NPKLH
Analysis
Pcbp1 BKO
IL6 IL3 SCF
μMT
-3
0
4
53
66 Days
B
# of cells
RV-Fdxr
Vector
IgM-PE Cy7
IgM MFI (10-3)
Vector
RV-Fdxr
C
Vector
RV-Fdxr
22.9
6.59
SSA
GFP
D
Pregated on GFP+CD19+ cells
Vector
RV-Fdxr
1.38
3.13
GL7-Per Cy5.5
CD95-Pacific blue
E
GC (%)
Vector
RV-FdxR
F
Pregated on NP+ GFP+ GCB cells
Vector
RV-Fdxr
4.74
11.0
CD86-PE CY7
CXCR4-APC
G
LZ(%)
Vector
RV-Fdxr
H
GCB cells
Fdxr-OE
Ctrl
# of cells
MitoTracker Deep Red
I
MitoTracker Deep Red MFI(10-3)
Vector
RV-Fdxr

Figure S12 *Pcbp1* maintains mitochondrial integrity to support IgM translation and germinal center response.

(**A**). Schematics outlining the reconstitution of *μMT* mice with *Fdxr*-overexpressing, *Pcbp1*-deficient B cells**.** Briefly, bone marrow cells were isolated from *Pcbp1* BKO mice that had been pre-treated with 5-fluorouracil (150 mg/kg) three days prior to collection. The isolated cells were cultured in medium supplemented with IL-3 (10 ng/mL), IL-6 (20 ng/mL), and SCF (50 ng/mL) and then transduced with either *Fdxr*-IRES-GFP (RV-*Fdxr)* or a control vector retrovirus at 20- and 40-hours post-culture. After puromycin selection, the cells were harvested on day 5 and transferred into *μMT* mice that had been sublethally irradiated (400 rad) one day prior to cell transfer. Six weeks after transplantation, the chimeric mice were immunized with NP-KLH. Thirteen days after the immunization, the differentiation of germinal center B (GCB) cells were analyzed.

(**B**). IgM expression in GFP^+ Splenic B Cells. IgM level was analyzed by flow cytometry in gated GFP^+ splenic B cells. (**B**). Representative flow cytometry plots. (**C**) Quantification of IgM MFI from three independent experiments. Data are shown as mean ± SD. Statistical significance was determined using Student's *t*-test (*, $p < 0.05$).

(**C**). Bone marrow cells isolated from *Pcbp1* BKO mice were cultured in medium supplemented with IL-3 (10 ng/mL), IL-6 (20 ng/mL), and SCF (50 ng/mL), transduced with either Fdxr-IRES-GFP (RV-Fdxr) or a control vector, and subsequently transferred into *μMT* mice. Six weeks after transplantation, the frequency of GFP-positive cells was assessed by flow cytometry.

(**D-I**). Bone marrow cells from *Pcbp1* BKO mice were retrovirally transduced with *Fdxr (*RV-*Fdxr)* or Ctrl and transferred into irradiated *μMT* mice. 6 weeks after the transplantation, the chimeric mice were immunized with NP-KLH. 13 days after NP-KLH immunization, germinal center B cells were analyzed:

(**D-E**). *Fdxr* over-expression restored GCB deficiency associated with *Pcbp1* deficiency. Ratios of germinal center B cells were determined by CD95 and GL7 staining, data are pregated at GFP^+ $B220^+CD19^+$ B cells in the spleen. (**D**), Representative of FACS plot. (**E**), Summary of 3 biological replicates in GFP^+ cells. **, $p < 0.01$ in Student's *t*-test.

(**F-G**). *Fdxr* promoted differentiation of light zone B cells in *Pcbp1* deficient B cells. Light zone and dark zone B cells were analyzed by CXCR4 and CD86 staining among $GFP^+NP^+CD95^+GL7^+$ B cells. (**F**), Representative of FACS plot. (**G**), Percentages of light zone B cells within GFP^+NP^+ GCB cells were summarized from 3 biological replicates, and each dot represents one mouse. *, $p < 0.05$ in Student's *t*-test.

(**H-I**). Mitochondrial Mass in GFP^+ Splenic B Cells. Mitochondrial mass was assessed in gated GFP^+ splenic B cells using MitoTracker Deep Red staining. (**H**). Representative flow cytometry plots. (**I**). Quantification from three independent experiments. Data are presented as mean ± SD. Statistical significance was determined using Student's *t*-test. *, $p < 0.05$.

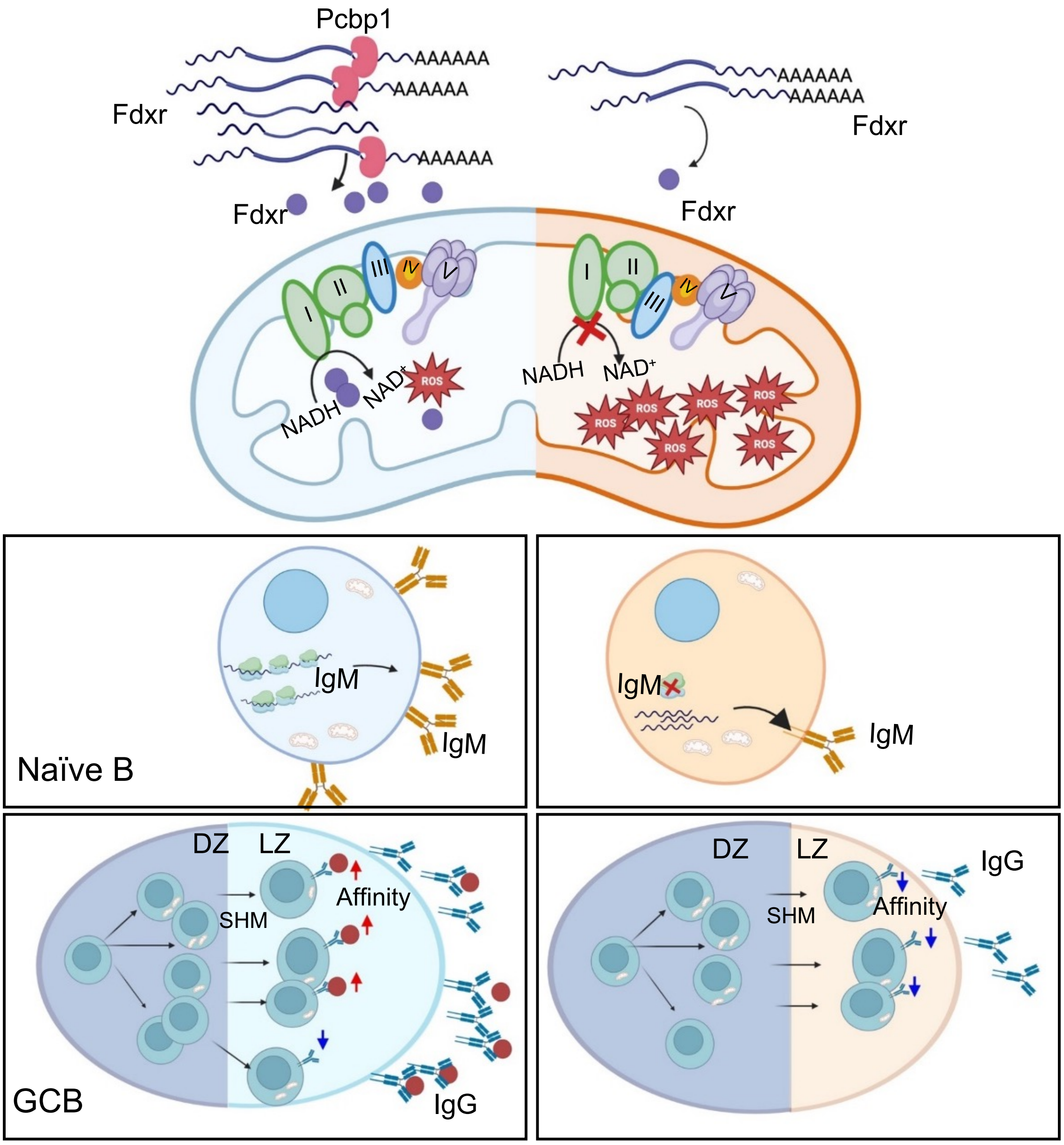

Wide type

Pcbp1–deficiency

Figure S13 A proposed model of this study

Pcbp1 binds to the 3' UTR of *Fdxr,* upregulating its expression to enhance mitochondrial complex I activity. Absence of *Pcbp1* resulted in reduced *Fdxr* expression, elevated mitochondrial ROS levels, leading to impaired IgM translation in naïve B cells and compromised germinal center responses following B cell activation.

Table S1 TMT proteomic analysis of Pcbp1 deficient naïve B cells

Entry	Protein names	Gene Names	logFC	AveExpr	t	P.Value	adj.P.Val	Subcellular location
Q8R0J4	Transmembrane protein 134	*Tmem134*	-2.341	6.201	-9.149	0.000	0.013	plasma membrane
Q9R0N0	Galactokinase (Galactose kinase)	*Galk1*	-2.293	6.226	-26.705	0.000	0.000	Cytoplasm, plasma membrane
O88673	Diacylglycerol kinase alpha	*Dgka*	-1.815	6.353	-9.286	0.000	0.013	Cytoplasm
A2AEV5	WD repeat domain 45	*Wdr45*	-1.518	6.446	-9.219	0.000	0.013	Cytoplasm
Q9QWV1	H2-O alpha (Histocompatibility 2, O region alpha locus)	*H2-Oa H2-Oalpha*	-1.483	6.453	-10.098	0.000	0.012	plasma membrane
P04104	Keratin, type II cytoskeletal 1	*Krt1 Krt2-1*	-1.388	6.248	-2.600	0.038	0.256	plasma membrane
P60335	Poly(rC)-binding protein 1 (Alpha-CP1) (Heterogeneous nuclear ribonucleoprotein E1) (hnRNP E1)	*Pcbp1*	-1.381	6.483	-14.085	0.000	0.005	nucleus
P50171	3R)-3-hydroxyacyl-CoA dehydrogenase	*Hsd17b8*	-1.280	6.504	-14.360	0.000	0.005	Mitochondrion matrix
Q9ERQ3	Zinc finger protein 704 (Glucocorticoid-induced gene 1 protein)	*Znf704 Gig1 Zfp704*	-1.254	6.507	-9.944	0.000	0.012	nucleus
Q91VT4	3-oxoacyl-[acyl-carrier-protein] reductase	*Cbr4*	-1.235	6.514	-15.104	0.000	0.005	Mitochondrion matrix
P05201	Vesicle transport protein GOT1A	*Got1*	-1.197	6.522	-15.375	0.000	0.005	Cytoplasm.
P70218	Mitogen-activated protein kinase kinase kinase kinase 1	*Map4k1*	-1.146	6.530	-11.326	0.000	0.011	Cytoplasm, plasma membrane
Q8BIG7	Catechol O-methyltransferase domain-containing protein 1	*Comtd1*	-1.142	6.514	-4.587	0.003	0.082	plasma membrane
Q91XB0	Three-prime repair exonuclease 1 (EC 3.1.11.2) (3'-5' exonuclease TREX1)	*Trex1*	-1.142	6.469	-2.678	0.034	0.246	Nucleus , plasma membrane
Q9DBK7	E1 ubiquitin-activating enzyme (EC 6.2.1.45)	*Uba7 Ube1l*	-1.133	6.474	-2.816	0.029	0.226	Nucleus, Cytoplasm
Q6P3E7	Polyamine deacetylase HDAC10 (EC 3.5.1.48) (EC 3.5.1.62) (Histone deacetylase 10) (HD10)	*Hdac10*	-1.086	6.537	-9.676	0.000	0.013	nuckeus+ Cytoplasm
Q8K4Q6	Endonuclease 8-like 1	*Neil1*	-1.047	6.548	-11.528	0.000	0.011	Cytoplasm, cytoskeleton,Nucleus
A0A0R4IZY9	tRNA-dihydrouridine(47) synthase [NAD(P)(+)]-like	*Dus3l*	-1.025	6.553	-12.044	0.000	0.011	Cytoplasm
Q3TIR3	Synembryn-A (Protein Ric-8A)	*Ric8a Ric8*	-0.997	6.552	-7.602	0.000	0.024	Cytoplasm
Q61578	NADPH:adrenodoxin oxidoreductase, mitochondrial	*Fdxr*	-0.996	6.554	-7.368	0.000	0.026	Mitochondrion matrix
P11911	B-cell antigen receptor complex-associated protein alpha chain	*Cd79a*	-0.979	6.503	-2.477	0.046	0.277	plasma membrane
Q8HWB2	Histocompatibility 2, Q region locus 4	*H2-Q4 H2-Gs10 H2-gs10 H2-Q1*	-0.944	6.555	-5.386	0.001	0.052	plasma membrane
Q9D2V7	Coronin-7 (Crn7) (70 kDa WD repeat tumor rejection antigen homolog)	*Coro7*	-0.911	6.572	-10.773	0.000	0.011	Golgi
Q5NCI3	DNA-directed DNA/RNA polymerase mu (EC 2.7.7.7)	*Polm*	-0.891	6.560	-4.396	0.004	0.090	nucleus
Q9EPB5	Serine hydrolase-like protein (SHL) (EC 3.1.-.-)	*Serhl*	-0.884	6.574	-9.755	0.000	0.013	Cytoplasm, Peroxisome
Q3UBZ5	MIF4G domain-containing protein	*Mif4gd*	-0.878	6.568	-5.126	0.002	0.061	Cytoplasm. Nucleus
Q8BHL7	CDC42 small effector protein 1	*Cdc42se1*	-0.872	6.539	-2.585	0.039	0.259	Cytoplasm, cytoskeleton, plasma membrane , Lipid-anchor
Q921H8	3-ketoacyl-CoA thiolase, peroxisomal	*Acaa1a Acaa1*	-0.848	6.580	-9.223	0.000	0.013	Peroxisome
O35424	Histocompatibility 2, O region beta locus (IAbeta2 subunit)	*H2-Ob H2-IAbeta2*	-0.842	6.577	-5.929	0.001	0.043	plasma membrane
Q78HU3	Multivesicular body subunit 12A (ESCRT-I complex subunit MVB12A) (Protein FAM125A)	*Mvb12a Fam125a*	-0.837	6.572	-5.511	0.001	0.050	Cytoplasm, cytoskeleton , Nucleus, Endosome
Q8VI75	Importin-4 (Imp4) (Importin-4a) (Imp4a) (Ran-binding protein 4) (RanBP4)	*Ipo4 Imp4a Ranbp4*	-0.833	6.582	-8.944	0.000	0.013	Cytoplasm, Nucleus

Q9DB05	Alpha-soluble NSF attachment protein (SNAP-alpha) (N-ethylmaleimide-sensitive factor attachment protein alpha)	*Napa Snapa*	-0.832	6.583	-10.796	0.000	0.011	plasma membrane
Q3ULW8	Protein mono-ADP-ribosyltransferase PARP3	*Parp3*	-0.829	6.584	-10.502	0.000	0.011	Nucleus, Cytoplasm, cytoskeleton
P04627	Serine/threonine-protein kinase A-Raf (EC 2.7.11.1) (Proto-oncogene A-Raf)	*Araf A-raf Araf1*	-0.827	6.567	-4.330	0.004	0.093	Cytoplasm,Mitochondrion
Q8VI94	2'-5'-oligoadenylate synthase-like protein 1 (2',5'-oligoadenylate synthetase-like 9)	*Oasl1 oasl9*	-0.826	6.564	-4.105	0.006	0.102	Nucleus, Cytoplasm
Q99M15	Proline-serine-threonine phosphatase-interacting protein 2 (PEST phosphatase-interacting protein 2) (Macrophage actin-associated tyrosine-phosphorylated protein) (pp37)	*Pstpip2 Mayp*	-0.820	6.578	-6.099	0.001	0.040	Cytoplasm, cytoskeleton , Nucleus, Endosome
Q7TQK5	Coiled-coil domain-containing protein 93	*Ccdc93*	-0.816	6.586	-11.357	0.000	0.011	Early endosome
O70400	PDZ and LIM domain protein 1 (C-terminal LIM domain protein 1) (Elfin) (LIM domain protein CLP-36)	*Pdlim1 Clim1*	-0.798	6.587	-8.286	0.000	0.017	Cytoplasm, cytoskeleton
P54726	UV excision repair protein RAD23 homolog A (HR23A) (mHR23A)	*Rad23a Mhr23a*	-0.795	6.570	-3.603	0.010	0.138	Nucleus
Q6ZPE2	Myotubularin-related protein 5 (Inactive phosphatidylinositol 3-phosphatase 5) (SET-binding factor 1) (Sbf1)	*Sbf1 Kiaa3020 Mtmr5*	-0.787	6.589	-8.534	0.000	0.016	Cytoplasm.
I1E4X5	VPS35 endosomal protein-sorting factor-like	*Vps35l 9030624J02Rik Vsp35l*	-0.783	6.589	-8.559	0.000	0.016	Endosome
Q8R1M0	Abasic site processing protein HMCES	*Hmces Srap1 Srapd1*	-0.781	6.574	-3.911	0.007	0.112	Nucleus
Q9QUG9	RAS guanyl-releasing protein 2 (Calcium and DAG-regulated guanine nucleotide exchange factor I) (CalDAG-GEFI) (F25B3.3 kinase-like protein)	*Rasgrp2*	-0.780	6.589	-7.257	0.000	0.027	Cytoplasm, plasma membrane
Q60841	Reelin (EC 3.4.21.-) (Reeler protein)	*Reln Rl*	-0.770	6.587	-5.731	0.001	0.047	Secreted
Q9D8Y0	EF-hand domain-containing protein D2 (Swiprosin-1)	*Efhd2 Sws1*	-0.764	6.583	-4.780	0.003	0.076	plasma membrane
Q8VD04	GRIP1-associated protein 1 (GRASP-1) (HCMV-interacting protein) [Cleaved into: GRASP-1 C-terminal chain (30kDa C-terminus form)]	*Gripap1 DXImx47e Kiaa1167*	-0.763	6.593	-10.787	0.000	0.011	Endosome
P35329	B-cell receptor CD22 (B-lymphocyte cell adhesion molecule) (BL-CAM) (Sialic acid-binding Ig-like lectin 2) (Siglec-2) (T-cell surface antigen Leu-14) (CD antigen CD22)	*Cd22 Lyb-8 Siglec2*	-0.752	6.582	-4.048	0.006	0.105	plasma membrane
A0A0G2JFZ3	Immunoglobulin kappa chain variable 19-93	*Igkv19-93*	-0.742	6.562	-3.137	0.019	0.179	plasma
A0A0H2UH19	Euchromatic histone methyltransferase 1	*plasma*	-0.740	6.596	-9.947	0.000	0.012	Nucleus
Q91X52	L-xylulose reductase (XR) (EC 1.1.1.10) (Dicarbonyl/L-xylulose reductase)	*Dcxr*	-0.740	6.588	-4.759	0.003	0.077	plasma membrane
Q7TNG5	Echinoderm microtubule-associated protein-like 2 (EMAP-2)	*Eml2*	-0.739	6.592	-6.355	0.001	0.037	Cytoplasm, cytoskeleton
P70268	Serine/threonine-protein kinase N1	*Pkn1*	-0.736	6.596	-9.243	0.000	0.013	Cytoplasm ,Nucleus
Q6GTM0	Ifit2 protein (Interferon-induced protein with tetratricopeptide repeats 2)	*Ifit2*	-0.733	6.587	-4.509	0.003	0.085	Cytoplasm,ER
P97314	Cysteine and glycine-rich protein 2 (Cysteine-rich protein 2) (CRP2) (Double LIM protein 1) (DLP-1)	*Csrp2 Dlp1*	-0.731	6.591	-5.311	0.001	0.055	Nucleus
Q9JLV6	Bifunctional polynucleotide phosphatase/kinase	*Pnkp*	-0.730	6.597	-8.773	0.000	0.015	Nucleus
O35654	DNA polymerase delta subunit 2 (DNA polymerase delta subunit p50)	*Pold2*	-0.716	6.593	-5.384	0.001	0.052	Nucleus
O88512	AP-1 complex subunit gamma-like 2 (Gamma2-adaptin) (G2ad)	*Ap1g2*	-0.714	6.598	-7.958	0.000	0.021	Golgi apparatus membrane Endosome membrane
E9Q0Y4	Signal-induced proliferation associated gene 1	*Sipa1*	-0.709	6.600	-9.244	0.000	0.013	Cytoplasm, Nucleus
P01901	H-2 class I histocompatibility antigen, K-B alpha chain (H-2K(B))	*H2-K1 H2-K*	-0.707	6.595	-6.013	0.001	0.042	plasma membrane
Q9QXJ2	Signal transducer and activator of transcription	*Stat2*	-0.700	6.597	-6.444	0.001	0.035	Cytoplasm, Nucleus

Q8K2Q0	COMM domain-containing protein 9	*Commd9*	-0.700	6.598	-6.204	0.001	0.039	Nucleus
F6U529	glutaminase (EC 3.5.1.2)	*Gls*	-0.678	6.598	-5.305	0.001	0.055	Mitochondrion
Q8VDP3	[F-actin]-monooxygenase MICAL1	*Mical1*	-0.673	6.603	-8.096	0.000	0.019	Cytoplasm,cytoskeleton ,Endosome membrane
Q08642	Protein-arginine deiminase type-2 (EC 3.5.3.15) (Peptidylarginine deiminase II) (Protein-arginine deiminase type II)	*Padi2 Pad2 Pdi Pdi2*	-0.667	6.603	-7.459	0.000	0.026	SUBCELLULAR LOCATION: Cytoplasm
P08920	T-cell surface antigen CD2 (LFA-2) (LFA-3 receptor) (Lymphocyte antigen 37) (Ly-37) (T-cell surface antigen T11/Leu-5) (CD antigen CD2)	*Cd2 Ly-37*	-0.661	6.589	-3.263	0.016	0.168	plasma membrane
Q9CZG3	COMM domain-containing protein 8	*Commd8*	-0.660	6.603	-6.753	0.000	0.032	Cytoplasm,Nucleus
Q61990	Poly(rC)-binding protein 2 (Alpha-CP2) (CTBP) (CBP) (Putative heterogeneous nuclear ribonucleoprotein X) (hnRNP X)	*Pcbp2 Cbp Hnrnpx Hnrpx*	-0.654	6.604	-6.594	0.000	0.034	Cytoplasm,Nucleus
F8VPK0	Superkiller complex protein 3 (Ski3)	*Skic3*	-0.650	6.605	-7.054	0.000	0.029	Cytoplasm,Nucleus
Q5U405	Transmembrane protease serine 13 (EC 3.4.21.-) (Membrane-type mosaic serine protease) (Mosaic serine protease)	*Tmprss13 Msp*	-0.641	6.595	-3.889	0.007	0.113	plasma membrane
O09046	L-amino-acid oxidase (LAAO) (LAO) (EC 1.4.3.2) (EC 1.4.3.25) (Interleukin-4-induced protein 1) (IL4-induced protein 1) (mIL4I1) (Protein Fig-1) (mFIG1)	*Il4i1 Fig1*	-0.633	6.605	-5.680	0.001	0.048	Secreted
P52503	NADH dehydrogenase [ubiquinone] iron-sulfur protein 6, mitochondrial (Complex I-13kD-A) (CI-13kD-A) (NADH-ubiquinone oxidoreductase 13 kDa-A subunit)	*Ndufs6 Ip13*	-0.630	6.593	-3.363	0.014	0.157	Mitochondrion
P35330	Intercellular adhesion molecule 2 (ICAM-2) (Lymphocyte function-associated AG-1 counter-receptor) (CD antigen CD102)	*Icam2 Icam-2*	-0.629	6.608	-7.618	0.000	0.024	plasma membrane
Q8R059	UDP-glucose 4-epimerase	*Gale*	-0.628	6.603	-4.824	0.002	0.074	Cytoplasm
P00920	Carbonic anhydrase 2 (EC 4.2.1.1) (Carbonate dehydratase II) (Carbonic anhydrase II) (CA-II) (Cyanamide hydratase CA2) (EC 4.2.1.69)	*Ca2 Car2*	-0.628	6.608	-7.924	0.000	0.021	Cytoplasm, plasma membrane
Q9QUH0	Glutaredoxin-1 (Thioltransferase-1) (TTase-1)	*Glrx Glrx1 Grx Grx1*	-0.620	6.573	-2.512	0.043	0.269	Cytoplasm.
O35075	Vacuolar protein sorting-associated protein 26C (Down syndrome critical region protein 3 homolog) (Down syndrome critical region protein A homolog)	*Vps26c Dcra Dscr3 Dscra*	-0.618	6.603	-4.439	0.004	0.088	Endosome
Q3V4B5	COMM domain-containing protein 6	*Commd6*	-0.615	6.604	-5.005	0.002	0.065	Nucleus ,Cytoplasm
P01887	Beta-2-microglobulin	*B2m*	-0.613	6.605	-4.763	0.003	0.077	Secreted.
P01644	Ig kappa chain V-V region HP R16.7		-0.612	6.604	-4.094	0.006	0.102	Cytoplasm
Q9JL16	Interferon-stimulated gene 20 kDa protein (EC 3.1.13.1) (Promyelocytic leukemia nuclear body-associated protein ISG20) (Protein DnaQL)	*Isg20*	-0.610	6.590	-2.602	0.038	0.256	Nucleus ,Cytoplasm , Nucleus, P-body
A0A0R4J0F5	Cytokine receptor-like factor 2	*Crlf2*	-0.605	6.601	-3.580	0.010	0.141	plasma membrane
P50396	Rab GDP dissociation inhibitor alpha (Rab GDI alpha) (Guanosine diphosphate dissociation inhibitor 1) (GDI-1)	*Gdi1 Rabgdia*	-0.602	6.606	-5.015	0.002	0.065	Cytoplasm , Golgi
Q9R117	Non-receptor tyrosine-protein kinase TYK2 (EC 2.7.10.2)	*Tyk2*	-0.599	6.610	-5.830	0.001	0.046	Cytoplasm ,Nucleus, plasma membrane
B8JJB3	PDZ and LIM domain 7	*Pdlim7*	-0.598	6.609	-5.506	0.001	0.050	Cytoplasm , cytoskeleton
A0A140T8N3	Immunoglobulin kappa chain variable 13-84	*Igkv13-84*	-0.598	6.607	-4.606	0.003	0.081	Cytoplasm
A0A0A6YVR9	SURF1-like protein	*Surf1*	-0.597	6.613	-9.323	0.000	0.013	Mitochondrion

P49615	Cyclin-dependent kinase 5	*Cdk5*	-0.594	6.606	-4.198	0.005	0.098	Nucleus,Cytoplasm ,plasma membrane
E9PWT4	Histocompatibility 2, Q region locus 7	*H2-Q7 H2-Q4*	-0.593	6.606	-4.498	0.004	0.085	plasma membrane
O55125	Protein NipSnap homolog 1 (NipSnap1)	*Nipsnap1*	-0.592	6.607	-5.075	0.002	0.062	Mitochondrion
E9Q4M2	Hormone-sensitive lipase (EC 3.1.1.23) (EC 3.1.1.79) (Monoacylglycerol lipase LIPE) (Retinyl ester hydrolase)	*Lipe*	-0.591	6.609	-5.377	0.001	0.052	plasma membrane ,Cytoplasm, Lipid droplet
Q8CBA2	Schlafen family member 5	*Slfn5*	-0.588	6.611	-6.149	0.001	0.040	Nucleus
P20693	Low affinity immunoglobulin epsilon Fc receptor (Fc-epsilon-RII) (Lymphocyte IgE receptor) (CD antigen CD23)	*Fcer2 Fcer2a*	-0.584	6.600	-3.102	0.019	0.185	plasma membrane , Lipid-anchor
Q99388	Component of Sp100-rs	*Csprs D1Lub1*	-0.577	6.600	-2.997	0.022	0.198	Nucleus
Q9QZK7	Docking protein 3 (Downstream of tyrosine kinase 3) (p62(dok)-like protein) (DOK-L)	*Dok3 Dokl*	-0.573	6.612	-5.716	0.001	0.047	plasma membrane ,Cytoplasm
Q3U1Y4	DENN domain-containing protein 4B (Brain-specific gene 4 protein) (Brain specific protein 4)	*Dennd4b Bsg4 Bsp4 Kiaa0476*	-0.573	6.611	-4.940	0.002	0.067	Golgi apparatus.
Q9R060	Cytosolic Fe-S cluster assembly factor NUBP1 (Nucleotide-binding protein 1) (NBP 1)	*Nubp1*	-0.571	6.609	-4.380	0.004	0.090	Cytoplasm,,Nucleus , cytoskeleton
Q99LC3	NADH dehydrogenase [ubiquinone] 1 alpha subcomplex subunit 10, mitochondrial	*Ndufa10*	-0.569	6.609	-4.303	0.004	0.093	Mitochondrion matrix
P55194	SH3 domain-binding protein 1 (3BP-1)	*Sh3bp1 3bp1*	-0.568	6.615	-7.014	0.000	0.029	Cell junction,Nucleus, Cytoplasm.
P28650	Adenylosuccinate synthetase isozyme 1	*Adss1 Adssl1*	-0.565	6.612	-5.592	0.001	0.049	Cytoplasm,plasma membrane
Q8R3I9	UDP-GlcNAc:betaGal beta-1,3-N-acetylglucosaminyltransferase 8	*B3gnt8 B3galt7*	-0.559	6.607	-3.868	0.007	0.114	Golgi
Q924T7	E3 ubiquitin-protein ligase RNF31	*Rnf31 Paul*	-0.555	6.606	-3.390	0.013	0.154	Cytoplasm
Q99J87	ATP-dependent RNA helicase DHX58	*Dhx58 D11lgp2e Lgp2*	-0.551	6.617	-7.315	0.000	0.026	Cytoplasm
Q8BK30	NADH dehydrogenase [ubiquinone] flavoprotein 3, mitochondrial (Complex I-9kD) (CI-9kD) (NADH-ubiquinone oxidoreductase 9 kDa subunit)	*Ndufv3*	-0.547	6.606	-3.234	0.016	0.171	Mitochondrion
P30416	Peptidyl-prolyl cis-trans isomerase FKBP4	*Fkbp4 Fkpb52*	-0.546	6.618	-8.281	0.000	0.017	Cytoplasm,,Mitochondrion, Nucleus , cytoskeleton
Q9DB52	P2R1A-PPP2R2A-interacting phosphatase regulator 1 (PABIR family member 1)	*Pabir1 Fam122a*	-0.545	6.602	-2.723	0.032	0.238	Nucleus, Cytoplasm .
A0A0R4J288	Decapping nuclease (EC 3.6.1.-)	*Dxo*	-0.543	6.610	-4.075	0.006	0.103	Nucleus
Q9R233	Tapasin (TPN) (TPSN) (TAP-associated protein) (TAP-binding protein)	*Tapbp Tapa*	-0.543	6.615	-5.558	0.001	0.049	ER
Q8CG76	Aflatoxin B1 aldehyde reductase member 2 (EC 1.1.1.n11) (Succinic semialdehyde reductase) (SSA reductase)	*Akr7a2 Afar Akr7a5*	-0.543	6.608	-3.457	0.012	0.149	Mitochondrion,Golgi apparatus,Cytoplasm
Q9DCJ5	NADH dehydrogenase [ubiquinone] 1 alpha subcomplex subunit 8	*Ndufa8*	-0.542	6.608	-3.248	0.016	0.169	Mitochondrion,
O35648	Centrin-3	*Cetn3 Cen3*	-0.541	6.613	-4.298	0.004	0.093	Cytoplasm, cytoskeleton,Nucleus
A2AS03	Helicase with zinc finger 2, transcriptional coactivator	*Helz2 BC006779*	-0.539	6.617	-6.087	0.001	0.040	Nucleus
Q80ZM5	H1 histone family, member X (H1.10 linker histone)	*H1f10 H1fx*	-0.536	6.592	-2.521	0.043	0.269	Nucleus

Q9D8N2	DENN domain-containing protein 10 (Protein FAM45A)	*Dennd10 Fam45a*	-0.533	6.616	-5.638	0.001	0.048	Late endosome
P36371	Antigen peptide transporter 2 (APT2) (EC 7.4.2.14) (ATP-binding cassette sub-family B member 3) (Histocompatibility antigen modifier 2)	*Tap2 Abcb3 Ham-2 Ham2*	-0.530	6.616	-4.717	0.003	0.077	ER
P17665	Cytochrome c oxidase subunit 7C, mitochondrial (Cytochrome c oxidase polypeptide VIIc)	*Cox7c Cox7c1*	-0.529	6.618	-6.472	0.001	0.035	Mitochondrion,
B1ATB3	Transmembrane channel-like protein	*Tmc6*	-0.529	6.601	-2.730	0.032	0.237	plasma membrane
Z4YKM2	CDGSH iron sulfur domain 3	*Cisd3*	-0.527	6.618	-6.544	0.000	0.034	Mitochondrion,
Q923D2	Flavin reductase (NADPH)	*Blvrb*	-0.526	6.618	-6.147	0.001	0.040	Cytoplasm
Q9Z0G0	PDZ domain-containing protein GIPC1	*Gipc1*	-0.525	6.613	-4.214	0.005	0.098	Cytoplasm,plasma membrane
Q9CQJ8	NADH dehydrogenase [ubiquinone] 1 beta subcomplex subunit 9 (Complex I-B22) (CI-B22) (NADH-ubiquinone oxidoreductase B22 subunit)	*Ndufb9*	-0.524	6.617	-4.969	0.002	0.066	Mitochondrion,
Q80X90	Filamin-B (FLN-B) (ABP-280-like protein) (Actin-binding-like protein) (Beta-filamin)	*Flnb*	-0.516	6.620	-7.235	0.000	0.027	Cytoplasm, , cytoskeleton.
P48771	Cytochrome c oxidase subunit 7A2, mitochondrial (Cytochrome c oxidase subunit VIIa-liver/heart) (Cytochrome c oxidase subunit VIIa-L)	*Cox7a2 Cox7a3 Cox7al*	-0.515	6.616	-4.286	0.004	0.093	Mitochondrion,
P03921	NADH-ubiquinone oxidoreductase chain 5 (EC 7.1.1.2) (NADH dehydrogenase subunit 5)	*Mtnd5 mt-Nd5 Nd5*	-0.514	6.610	-3.096	0.020	0.185	Mitochondrion,
Q3V089	RNA-binding protein 44 (RNA-binding motif protein 44)	*Rbm44 Gm817*	-0.513	6.603	-2.441	0.048	0.284	Cytoplasm
P52431	DNA polymerase delta catalytic subunit (EC 2.7.7.7) (3'-5' exodeoxyribonuclease) (EC 3.1.11.-)	*Pold1*	-0.505	6.621	-7.897	0.000	0.021	Nucleus, Cytoplasm.
E9Q8I9	Protein furry homolog	*Fry Kiaa4143*	-0.504	6.614	-3.511	0.011	0.144	Cytoplasm,cytoskeleton
Q3TBA3	Transporter 1, ATP-binding cassette, sub-family B (MDR/TAP)	*Tap1*	-0.504	6.622	-7.402	0.000	0.026	plasma membrane
F8WIW2	Lysozyme g-like protein (EC 3.2.1.-)	*Lyg1*	-0.501	6.615	-3.904	0.007	0.113	Secreted
F6WIU1	Protein KRI1 homolog	*Kri1*	-0.498	6.613	-3.390	0.013	0.154	Nucleus
Q9CZN7	Serine hydroxymethyltransferase, mitochondrial	*Shmt2*	-0.497	6.618	-4.405	0.004	0.089	Mitochondrion,
P01899	H-2 class I histocompatibility antigen, D-B alpha chain (H-2D(B))	*H2-D1*	-0.496	6.622	-7.340	0.000	0.026	plasma membrane
Q60596	DNA repair protein XRCC1 (X-ray repair cross-complementing protein 1)	*Xrcc1 Xrcc-1*	-0.495	6.621	-5.712	0.001	0.047	Nucleus
D6RFU9	Synaptophysin-like protein	*Sypl1 Sypl*	-0.495	6.610	-2.756	0.031	0.234	plasma membrane
Q9JIG7	Coiled-coil domain-containing protein 22	*Ccdc22 DXImx40e*	-0.493	6.622	-6.982	0.000	0.029	Endosome,Cytoplasm, cytoskeleton
Q9ET39	SLAM family member 6 (Lymphocyte antigen 108) (CD antigen CD352)	*Slamf6 Ly108*	-0.492	6.620	-4.720	0.003	0.077	plasma membrane
Z4YNA3	MAU2 chromatid cohesion factor homolog (Cohesin loading complex subunit SCC4 homolog)	*Mau2*	-0.491	6.615	-3.465	0.012	0.147	Nucleus
F6ZBR8	Widely-interspaced zinc finger motifs	*Wiz*	-0.484	6.618	-4.311	0.004	0.093	Nucleus
P23591	GDP-L-fucose synthase	*Gfus*	-0.483	6.622	-5.934	0.001	0.043	Mitochondrion,
P59941	NAD-dependent protein deacylase sirtuin-6	*Sirt6 Sir2l6*	-0.482	6.609	-2.746	0.031	0.235	Nucleus
Q9WUB0	RanBP-type and C3HC4-type zinc finger-containing protein 1	*Rbck1 Rbck Ubce7ip3 Uip28*	-0.480	6.622	-5.982	0.001	0.042	Cytoplasm

Q3UIU2	NADH dehydrogenase [ubiquinone] 1 beta subcomplex subunit 6 (Complex I-B17) (CI-B17) (NADH-ubiquinone oxidoreductase B17 subunit)	*Ndufb6 Gm137*	-0.480	6.620	-4.461	0.004	0.086	Mitochondrion,
Q9CY21	18S rRNA (guanine-N(7))-methyltransferase	*Bud23 Wbscr22*	-0.476	6.617	-3.307	0.015	0.163	Nucleus, Cytoplasm
Q7TMF3	NADH dehydrogenase [ubiquinone] 1 alpha subcomplex subunit 12	*Ndufa12*	-0.474	6.613	-2.838	0.028	0.224	Mitochondrion,
Q3UZ35	CD72 antigen	*Cd72*	-0.474	6.621	-4.708	0.003	0.077	plasma membrane
Q91WA6	Sharpin (Shank-associated RH domain-interacting protein) (Shank-interacting protein-like 1) (mSIPL1)	*Sharpin Cpdm Sipl1*	-0.473	6.619	-4.172	0.005	0.098	Cytoplasm
Q9Z1K6	E3 ubiquitin-protein ligase ARIH2	*Arih2*	-0.473	6.615	-3.125	0.019	0.180	Nucleus, Cytoplasm.
A0A0G2JFE9	Immunoglobulin heavy variable 1-76	*Ighv1-76*	-0.466	6.623	-5.006	0.002	0.065	Secreted
Q8CB96	Ras association domain-containing protein 4	*Rassf4*	-0.466	6.623	-5.146	0.002	0.060	Cytoplasm
Q9CY94	DNA replication complex GINS protein PSF3 (GINS complex subunit 3)	*Gins3 Psf3*	-0.465	6.616	-3.135	0.019	0.179	Nucleus
Q9WUM4	Coronin-1C (Coronin-3)	*Coro1c*	-0.465	6.622	-4.896	0.002	0.070	plasma membrane
P19783	Cytochrome c oxidase subunit 4 isoform 1, mitochondrial	*Cox4i1 Cox4 Cox4a*	-0.462	6.623	-5.185	0.002	0.059	Mitochondrion,
E9Q6W2	Cytochrome c oxidase assembly factor 3	*Coa3 Ccdc56*	-0.459	6.621	-4.321	0.004	0.093	plasma membrane
P03958	Adenosine deaminase (EC 3.5.4.4) (Adenosine aminohydrolase)	*Ada*	-0.459	6.622	-4.305	0.004	0.093	plasma membrane
D3Z1Y1	Synovial apoptosis inhibitor 1, synoviolin	*Syvn1*	-0.458	6.619	-3.277	0.015	0.167	ER
Q8VH51	RNA-binding protein 39	*Rbm39 Caper Rnpc2*	-0.456	6.617	-3.245	0.016	0.169	Nucleus
P16045	Galectin-1	*Lgals1 Gbp*	-0.455	6.620	-3.852	0.007	0.114	Secreted
Q6NZR5	Superkiller complex protein 2 (Ski2) (EC 3.6.4.13)	*Skic2*	-0.455	6.614	-2.686	0.034	0.245	Nucleus {ECO:0000250|UniProtKB:Q15477}. Cytoplasm {ECO:0000250|UniProtKB:Q15477}.
P11531	Dystrophin	*Dmd*	-0.453	6.617	-3.075	0.020	0.189	plasma membrane
P98192	Dihydroxyacetone phosphate acyltransferase	*Gnpat Dhapat*	-0.452	6.626	-6.749	0.000	0.032	Peroxisome membrane
P70227	Inositol 1,4,5-trisphosphate-gated calcium channel ITPR3	*Itpr3*	-0.450	6.626	-6.334	0.001	0.038	ER
Q9CWP8	DNA polymerase delta subunit 4 (DNA polymerase delta subunit p12)	*Pold4*	-0.449	6.621	-3.869	0.007	0.114	Nucleus
Q99JZ4	SAR1 gene homolog A (S. cerevisiae) (Secretion associated Ras related GTPase 1A)	*Sar1a Sara1*	-0.449	6.623	-4.552	0.003	0.084	ER, GOLGi
B2RUP2	Protein unc-13 homolog D (Munc13-4)	*Unc13d*	-0.447	6.617	-3.005	0.022	0.197	Cytoplasm
P57780	Alpha-actinin-4 (Non-muscle alpha-actinin 4)	*Actn4*	-0.444	6.625	-5.428	0.001	0.051	Nucleus, Cytoplasm.
Q9Z0F4	Calcium and integrin-binding protein 1 (CIB) (Calmyrin) (DNA-PKcs-interacting protein) (Kinase-interacting protein) (KIP)	*Cib1 Cib Kip Prkdcip*	-0.443	6.619	-3.243	0.016	0.169	plasma membrane ; Lipid-anchor
P70122	Ribosome maturation protein SBDS (Protein 22A3) (Shwachman-Bodian-Diamond syndrome protein homolog)	*Sbds*	-0.441	6.625	-5.431	0.001	0.051	Cytoplasm. Nucleus
Q9CPP6	NADH dehydrogenase [ubiquinone] 1 alpha subcomplex subunit 5	*Ndufa5*	-0.441	6.620	-3.266	0.016	0.168	Mitochondrion,
A0A0R4J073	BRCA1-A complex subunit RAP80 (Receptor-associated protein 80) (Ubiquitin interaction motif-containing protein 1)	*Uimc1*	-0.440	6.621	-3.644	0.010	0.134	Nucleus

Q9CQ02	COMM domain-containing protein 4	*Commd4*	-0.440	6.623	-4.221	0.005	0.098	Cytoplasm {ECO:0000250|UniProtKB:Q9H0A8}. Nucleus {ECO:0000250|UniProtKB:Q9H0A8}.
Q8BMK1	tRNA N(3)-methylcytidine methyltransferase METTL2 (EC 2.1.1.-) (Methyltransferase-like protein 2)	*Mettl2 D11Ertd768e*	-0.439	6.620	-3.473	0.012	0.146	Cytoplasm
Q9JM14	5'(3')-deoxyribonucleotidase, cytosolic type (EC 3.1.3.-) (Cytosolic 5',3'-pyrimidine nucleotidase) (Deoxy-5'-nucleotidase 1) (dNT-1)	*Nt5c Dnt1*	-0.439	6.626	-5.634	0.001	0.048	Cytoplasm {ECO:0000269|PubMed:10681516}.
Q811U4	Mitofusin-1 (EC 3.6.5.-) (Transmembrane GTPase MFN1)	*Mfn1*	-0.438	6.616	-2.628	0.037	0.254	Mitochondrion,
A0A0R4J140	Clustered mitochondria protein homolog	*Cluh*	-0.437	6.622	-3.688	0.009	0.129	Cytoplasm
P01631	Ig kappa chain V-II region 26-10		-0.435	6.614	-2.499	0.044	0.272	secreted
P14069	Protein S100-A6 (5B10) (Calcyclin) (Prolactin receptor-associated protein) (S100 calcium-binding protein A6)	*S100a6 Cacy*	-0.434	6.617	-2.913	0.025	0.210	Nucleus ,Cytoplasm {ECO:0000250}. plasma membrane
Q922B1	ADP-ribose glycohydrolase MACROD1	*Macrod1 Lrp16*	-0.430	6.619	-3.136	0.019	0.179	Nucleus
Q9WUD1	E3 ubiquitin-protein ligase CHIP (EC 2.3.2.27) (Carboxy terminus of Hsp70-interacting protein) (RING-type E3 ubiquitin transferase CHIP) (STIP1 homology and U box-containing protein 1)	*Stub1 Chip*	-0.429	6.621	-3.223	0.017	0.172	Cytoplasm {ECO:0000269|PubMed:11435423}. Nucleus
A0A0R3P9C8	NADH:ubiquinone oxidoreductase subunit A9	*Ndufa9*	-0.425	6.627	-5.096	0.002	0.062	Mitochondrion,
Q8CIS0	Caspase recruitment domain-containing protein 11 (CARD-containing MAGUK protein 1) (Carma 1)	*Card11 Carma1*	-0.424	6.628	-6.186	0.001	0.039	Cytoplasm ,plasma membrane
Q64324	Syntaxin-binding protein 2 (MUSEC1) (Protein unc-18 homolog 2) (Munc18-2) (Unc18-2) (Protein unc-18 homolog B) (Unc-18B)	*Stxbp2 Unc18b*	-0.420	6.628	-6.216	0.001	0.039	plasma membrane ,Cytoplasm,EXOSOME
E9Q3U1	Ubiquitin-conjugating enzyme E2H	*Ube2h*	-0.420	6.626	-4.713	0.003	0.077	Cytoplasm, Nucleus
Q9DBC7	cAMP-dependent protein kinase type I-alpha regulatory subunit [Cleaved into: cAMP-dependent protein kinase type I-alpha regulatory subunit, N-terminally processed]	*Prkar1a*	-0.419	6.628	-6.060	0.001	0.041	plasma membrane
Q2YDW2	Protein misato homolog 1	*Msto1*	-0.417	6.625	-4.109	0.005	0.102	Mitochondrion,
Q9DCS9	NADH dehydrogenase [ubiquinone] 1 beta subcomplex subunit 10	*Ndufb10*	-0.416	6.622	-3.077	0.020	0.189	Mitochondrion
Q920E5	Farnesyl pyrophosphate synthase	*Fdps*	-0.415	6.620	-2.888	0.026	0.214	Cytoplasm
Q9Z2B9	Ribosomal protein S6 kinase alpha-4	*Rps6ka4 Msk2*	-0.412	6.627	-5.017	0.002	0.065	Nucleus
P58137	Acyl-coenzyme A thioesterase 8	*Acot8 Pte1*	-0.412	6.625	-3.964	0.007	0.110	Peroxisome matrix
B1ATP7	Zinc phosphodiesterase ELAC protein 2 (EC 3.1.26.11) (ElaC homolog protein 2) (Ribonuclease Z 2) (tRNA 3 endonuclease 2) (tRNase Z 2)	*Elac2*	-0.411	6.627	-4.604	0.003	0.081	Mitochondrion
D3YUM1	NADH dehydrogenase [ubiquinone] flavoprotein 1, mitochondrial (EC 7.1.1.2)	*Ndufv1*	-0.410	6.625	-3.825	0.008	0.116	Mitochondrion,
P14483	H-2 class II histocompatibility antigen, A beta chain	*H2-Ab1 H2-iabeta*	-0.407	6.628	-5.176	0.002	0.059	plasma membrane
B1ATS5	Calcium-transporting ATPase (EC 7.2.2.10)	*Atp2a3*	-0.404	6.629	-6.103	0.001	0.040	ER
Q3U7R1	Extended synaptotagmin-1 (E-Syt1) (Membrane-bound C2 domain-containing protein)	*Esyt1 Fam62a Mbc2*	-0.403	6.630	-6.423	0.001	0.036	ER

P00405	Cytochrome c oxidase subunit 2 (EC 7.1.1.9) (Cytochrome c oxidase polypeptide II)	*Mtco2 COII COX2 mt-Co2*	-0.403	6.629	-6.121	0.001	0.040	Mitochondrion,
Q9EPQ7	StAR-related lipid transfer protein 5 (START domain-containing protein 5) (StARD5)	*Stard5*	-0.401	6.625	-3.694	0.009	0.129	Cytoplasm
Q9Z256	P2X purinoceptor	*P2rx4*	-0.398	6.619	-2.561	0.041	0.263	plasma membrane
Q3U5Q7	UMP-CMP kinase 2, mitochondrial (EC 2.7.4.14) (Nucleoside-diphosphate kinase) (EC 2.7.4.6) (Thymidylate kinase LPS-inducible member) (TYKi)	*Cmpk2 Tyki*	-0.397	6.628	-4.634	0.003	0.080	Mitochondrion,
Q9WTL7	Acyl-protein thioesterase 2 (APT-2) (EC 3.1.2.-) (Lysophospholipase 2) (Lysophospholipase II) (LPL-II) (LysoPLA II) (mLyso II) (Palmitoyl-protein hydrolase) (EC 3.1.2.22)	*Lypla2*	-0.396	6.630	-5.541	0.001	0.050	Cytoplasm
Q9D6J5	NADH dehydrogenase [ubiquinone] 1 beta subcomplex subunit 8	*Ndufb8*	-0.395	6.625	-3.490	0.012	0.144	Mitochondrion,
Q9EQQ9	Protein O-GlcNAcase (OGA) (EC 3.2.1.169) (Beta-N-acetylhexosaminidase) (Beta-hexosaminidase) (Bifunctional protein NCOAT) (Meningioma-expressed antigen 5) (N-acetyl-beta-D-glucosaminidase) (N-acetyl-beta-glucosaminidase)	*Oga Hexc Kiaa0679 Mgea5*	-0.394	6.628	-4.426	0.004	0.088	Nucleus {ECO:0000250}. Cytoplasm {ECO:0000250}.
Q3UUT0	Eukaryotic translation initiation factor 4E member 2	*Eif4e2*	-0.392	6.627	-3.638	0.010	0.134	Cytoplasm.P-BODY
Q4VBD2	Transmembrane anterior posterior transformation protein 1	*Tapt1*	-0.391	6.624	-3.177	0.018	0.175	Cytoplasm, cytoskeleton,Cytoplasm
P12787	Cytochrome c oxidase subunit 5A, mitochondrial (Cytochrome c oxidase polypeptide Va)	*Cox5a*	-0.390	6.625	-3.179	0.018	0.175	Mitochondrion,
Q61635	GTP-binding protein (Interferon gamma inducible protein 47) (Interferon-gamma-inducible GTPase Ifggc1 protein)	*Ifi47 Ifggc1 IRG-47*	-0.389	6.627	-3.599	0.010	0.138	ER
Q3U429	Tetraspanin	*Cd37*	-0.387	6.630	-5.260	0.002	0.056	plasma membrane
Q3UDT3	phosphatidylinositol-4,5-bisphosphate 3-kinase (EC 2.7.1.153)	*Pik3cd*	-0.386	6.630	-5.929	0.001	0.043	Cytoplasm
E9QAN8	Phosphatidylinositol-4-phosphate 3-kinase catalytic subunit type 2 beta	*Pik3c2b*	-0.386	6.630	-5.338	0.001	0.054	Cytoplasm. Plasma membrabe
Q9R061	Cytosolic Fe-S cluster assembly factor NUBP2 (Nucleotide-binding protein 2) (NBP 2)	*Nubp2*	-0.386	6.625	-3.099	0.019	0.185	Nucleus . Cytoplasm,
E9QP49	EH domain binding protein 1-like 1	*Ehbp1l1*	-0.386	6.620	-2.515	0.043	0.269	Endosome
Q9D4V0	Ethanolamine kinase 1 (EKI 1) (EC 2.7.1.82)	*Etnk1 Eki1*	-0.385	6.629	-4.435	0.004	0.088	Cytoplasm
Q8C0G2	TRAF3-interacting JNK-activating modulator (TRAF3-interacting protein 3)	*Traf3ip3 T3jam*	-0.385	6.622	-2.633	0.037	0.254	plasma membrane , Golgi apparatus ,Lysosome membrane ,Mitochondrion
Q920A9	Fc receptor-like A	*Fcrla*	-0.383	6.628	-3.817	0.008	0.117	Cytoplasm , secreted.
Q63829	COMM domain-containing protein 3 (Bmi-1 upstream gene protein) (Bup protein)	*Commd3 Bup*	-0.382	6.629	-4.369	0.004	0.090	Cytoplasm , Nucleus
Q4PZA2	Endothelin-converting enzyme 1 (ECE-1) (EC 3.4.24.71)	*Ece1*	-0.381	6.627	-3.436	0.013	0.151	plasma membrane
O35683	NADH dehydrogenase [ubiquinone] 1 alpha subcomplex subunit 1 (Complex I-MWFE) (CI-MWFE) (NADH-ubiquinone oxidoreductase MWFE subunit)	*Ndufa1*	-0.380	6.625	-2.956	0.024	0.204	Mitochondrion,
A0A0A6YWV7	Interferon-related developmental regulator 2	*Ifrd2*	-0.379	6.630	-4.752	0.003	0.077	Nucleus,

P57725	SAM domain-containing protein SAMSN-1 (SAM domain, SH3 domain and nuclear localization signals protein 1) (SH3 protein expressed in lymphocytes 2) (SH3-lymphocyte protein 2) (SLy2)	*Samsn1*	-0.377	6.624	-2.762	0.031	0.233	Nucleus. Cytoplasm,cytoskeleton
P08103	Tyrosine-protein kinase HCK (EC 2.7.10.2) (B-cell/myeloid kinase) (BMK) (Hematopoietic cell kinase) (Hemopoietic cell kinase) (p56-HCK/p59-HCK)	*Hck*	-0.377	6.631	-5.255	0.002	0.056	Cytoplasm
Q62425	Cytochrome c oxidase subunit NDUFA4	*Ndufa4*	-0.374	6.627	-3.510	0.011	0.144	Mitochondrion,
P17918	Proliferating cell nuclear antigen (PCNA) (Cyclin)	*Pcna*	-0.374	6.629	-4.203	0.005	0.098	Nucleus
A0A286YCS4	Anion exchange protein	*Slc4a7*	-0.373	6.623	-2.697	0.034	0.243	plasma membrane
Q8BSH9	Nucleosome assembly protein 1-like 1	*Nap1l1*	-0.367	6.630	-4.019	0.006	0.107	Cytoplasm, Nucleus
P06328	Ig heavy chain V region 1-72 (Ig heavy chain V region VH558 B4)	*Ighv1-72*	-0.366	6.625	-2.826	0.028	0.225	secreted
Q9D6X6	Serine protease 23 (EC 3.4.21.-)	*Prss23*	-0.365	6.625	-2.799	0.029	0.227	Secreted
P14434	H-2 class II histocompatibility antigen, A-B alpha chain (IAalpha)	*H2-Aa*	-0.365	6.630	-3.956	0.007	0.110	plasma membrane
H7BX88	Carnitine acetyltransferase	*Crat*	-0.365	6.631	-4.380	0.004	0.090	ER.Mitochondrion,
Q9ERI5	Bifunctional arginine demethylase and lysyl-hydroxylase JMJD6	*Jmjd6 Kiaa0585 Ptdsr*	-0.363	6.625	-2.890	0.026	0.214	Nucleus,
Q99MK8	Beta-adrenergic receptor kinase 1 (Beta-ARK-1) (EC 2.7.11.15) (G-protein-coupled receptor kinase 2)	*Grk2 Adrbk1*	-0.363	6.627	-2.963	0.023	0.203	Cytoplasm {ECO:0000250|UniProtKB:P26817}. Cell membrane {ECO:0000250
F8VQL9	Nuclear receptor co-repressor 2	*Ncor2*	-0.362	6.631	-4.386	0.004	0.090	Nucleus
Q80U62	Run domain Beclin-1-interacting and cysteine-rich domain-containing protein (Rubicon)	*Rubcn Kiaa0226*	-0.361	6.630	-3.841	0.008	0.115	Late endosome, Lysosome ,Early endosome;
A2AUM9	Centrosomal protein of 152 kDa (Cep152)	*Cep152 Kiaa0912*	-0.361	6.627	-3.186	0.017	0.175	Cytoplasm, cytoskeleton,
Q8K285	F-BAR domain only protein 1	*Fcho1*	-0.360	6.629	-3.493	0.012	0.144	plasma membrane
P97369	Neutrophil cytosol factor 4 (NCF-4) (Neutrophil NADPH oxidase factor 4) (p40-phox) (p40phox)	*Ncf4*	-0.358	6.628	-3.239	0.016	0.170	Cytoplasm, ,Endosome membrane
E9QLK3	BRCA1-associated ATM activator 1	*Brat1 Baat1*	-0.357	6.628	-3.003	0.022	0.197	nuckeus+ Cytoplasm
D3YWD5	Major facilitator superfamily domain containing 10	*Mfsd10*	-0.357	6.629	-3.559	0.011	0.142	plasma membrane
P19536	Cytochrome c oxidase subunit 5B, mitochondrial (Cytochrome c oxidase polypeptide Vb)	*Cox5b*	-0.356	6.631	-4.516	0.003	0.085	Mitochondrion inner membrane ; Peripheral membrane protein ; Matrix side
Q9D6J6	NADH dehydrogenase [ubiquinone] flavoprotein 2, mitochondrial (EC 7.1.1.2) (NADH-ubiquinone oxidoreductase 24 kDa subunit)	*Ndufv2*	-0.355	6.630	-3.571	0.011	0.141	Mitochondrion inner membrane ; Peripheral membrane protein; Matrix side
Q3ULJ0	Glycerol-3-phosphate dehydrogenase 1-like protein (EC 1.1.1.8)	*Gpd1l Kiaa0089*	-0.354	6.631	-4.100	0.006	0.102	Cytoplasm {ECO:0000305}.
E9QJV4	Pre-mRNA processing factor 39	*Prpf39*	-0.354	6.631	-4.494	0.004	0.085	Nucleus
Q9CQH3	NADH dehydrogenase [ubiquinone] 1 beta subcomplex subunit 5, mitochondrial (Complex I-SGDH) (CI-SGDH) (NADH-ubiquinone oxidoreductase SGDH subunit)	*Ndufb5*	-0.353	6.631	-3.985	0.006	0.109	Mitochondrion inner membrane ; Single-pass membrane protein; Matrix side
E9Q9M5	ubiquitinyl hydrolase 1 (EC 3.4.19.12)	*Usp19*	-0.353	6.629	-3.346	0.014	0.159	ER
Q9CY27	Very-long-chain enoyl-CoA reductase (EC 1.3.1.93) (Synaptic glycoprotein SC2) (Trans-2,3-enoyl-CoA reductase) (TER)	*Tecr Gpsn2*	-0.353	6.625	-2.557	0.041	0.263	Endoplasmic reticulum membrane {ECO:0000250|UniProtKB:Q9NZ01}; Multi-pass membrane protein {ECO:0000255}.
A2BE28	Ribosomal biogenesis protein LAS1L (Protein LAS1 homolog)	*Las1l*	-0.348	6.629	-3.221	0.017	0.172	Nucleus, Cytoplasm

Q8BTM8	Filamin-A (FLN-A) (Actin-binding protein 280) (ABP-280) (Alpha-filamin) (Endothelial actin-binding protein) (Filamin-1) (Non-muscle filamin)	*Flna Fln Fln1*	-0.346	6.632	-4.362	0.004	0.090	Cytoplasm
Q91VD9	NADH-ubiquinone oxidoreductase 75 kDa subunit, mitochondrial (EC 7.1.1.2) (Complex I-75kD) (CI-75kD)	*Ndufs1*	-0.346	6.631	-3.861	0.007	0.114	Mitochondrion inner membrane ; Peripheral membrane protein.
A0A087WRH2	Fas apoptotic inhibitory molecule	*Faim*	-0.345	6.630	-3.341	0.014	0.159	secreted,
E9Q405	Myosin XVIIIA	*Myo18a*	-0.344	6.633	-4.739	0.003	0.077	golgi, plasma membrane, CYTOSkeleton
Q8BQY8	Checkpoint protein HUS1 (mHUS1)	*Hus1*	-0.343	6.629	-3.053	0.021	0.192	Nucleus;Cytoplasm
Q61081	Hsp90 co-chaperone Cdc37 (Hsp90 chaperone protein kinase-targeting subunit) (p50Cdc37) [Cleaved into: Hsp90 co-chaperone Cdc37, N-terminally processed]	*Cdc37*	-0.342	6.630	-3.479	0.012	0.145	Cytoplasm
E9Q4S7	protein-tyrosine-phosphatase (EC 3.1.3.48)	*Ptprj*	-0.342	6.632	-3.995	0.006	0.109	PLasma membrane
Q8BMD8	Mitochondrial adenyl nucleotide antiporter SLC25A24	*Slc25a24 Scamc1*	-0.341	6.629	-3.070	0.020	0.189	Mitochondrion
A2ADZ8	Tyrosine-protein kinase Fyn (EC 2.7.10.2) (Proto-oncogene c-Fyn) (p59-Fyn)	*Fyn*	-0.341	6.629	-3.219	0.017	0.172	Cytoplasm ,Nucleus ,PLasma membrane
P39688	NADH dehydrogenase [ubiquinone] 1 alpha subcomplex subunit 13	*Ndufa13 Grim19*	-0.340	6.633	-4.667	0.003	0.079	Mitochondrion,
Q9ERS2	Lysophospholipid acyltransferase 5	*Lpcat3*	-0.340	6.633	-4.178	0.005	0.098	ER
Q91V01	Signal transducer and transcription activator 6	*Stat6*	-0.339	6.625	-2.436	0.048	0.284	Cytoplasm. Nucleus
P52633	Neutrophil cytosol factor 1 (NCF-1) (47 kDa neutrophil oxidase factor) (NCF-47K) (Neutrophil NADPH oxidase factor 1) (p47-phox)	*Ncf1*	-0.339	6.634	-5.448	0.001	0.051	Cytoplasm
Q09014	Destrin (Actin-depolymerizing factor) (ADF) (Sid 23)	*Dstn Dsn Sid23*	-0.338	6.633	-4.648	0.003	0.080	Cytoplasm
Q2TBA3	Mucosa-associated lymphoid tissue lymphoma translocation protein 1 homolog (EC 3.4.22.-) (Paracaspase)	*Malt1*	-0.336	6.632	-3.883	0.007	0.114	Cytoplasm
E9PZ88	alpha-mannosidase (EC 3.2.1.24)	*Man2c1*	-0.336	6.633	-4.175	0.005	0.098	Cytoplasm
Q9CQ54	NADH dehydrogenase [ubiquinone] 1 subunit C2 (Complex I-B14.5b) (CI-B14.5b) (NADH-ubiquinone oxidoreductase subunit B14.5b)	*Ndufc2*	-0.334	6.633	-4.498	0.004	0.085	Mitochondrion,
O70572	Sphingomyelin phosphodiesterase 2 (EC 3.1.4.12) (Lyso-platelet-activating factor-phospholipase C) (Lyso-PAF-PLC) (Neutral sphingomyelinase) (N-SMase) (nSMase)	*Smpd2*	-0.334	6.632	-3.953	0.007	0.110	plasma membrane
F6T4M4	Serine/arginine repetitive matrix 1	*Srrm1*	-0.334	6.633	-4.503	0.004	0.085	Nucleus
Q52L52	Heat shock factor 1 (Hsf1 protein)	*Hsf1*	-0.333	6.630	-3.098	0.020	0.185	Nucleus
Q8VDD8	WASH complex subunit 1 (WAS protein family homolog 1)	*Washc1 Orf19 Wash Wash1*	-0.331	6.631	-3.285	0.015	0.166	Early endosome membrane ; Peripheral membrane protein ; Cytoplasmic side ; Recycling endosome membrane ; Peripheral membrane protein
Q9CQ91	NADH dehydrogenase [ubiquinone] 1 alpha subcomplex subunit 3 (Complex I-B9) (CI-B9) (NADH-ubiquinone oxidoreductase B9 subunit)	*Ndufa3*	-0.330	6.632	-3.914	0.007	0.112	Mitochondrion
P59041	DnaJ homolog subfamily C member 30, mitochondrial (Williams-Beuren syndrome chromosomal region 18 protein homolog)	*Dnajc30 Wbscr18*	-0.330	6.632	-3.545	0.011	0.142	Mitochondrion
D3YUE7	GRAM domain containing 4	*Gramd4*	-0.330	6.632	-3.895	0.007	0.113	Mitochondrion,ER
O08796	Eukaryotic elongation factor 2 kinase (eEF-2 kinase) (eEF-2K) (EC 2.7.11.20) (Calcium/calmodulin-dependent eukaryotic elongation factor 2 kinase)	*Eef2k*	-0.329	6.626	-2.531	0.042	0.267	Cytoplasm
E9QJS7	Adhesion G protein-coupled receptor E5	*Adgre5 Cd97*	-0.326	6.628	-2.420	0.049	0.286	plasma membrane

P19096	Fatty acid synthase	*Fasn*	-0.325	6.634	-4.467	0.004	0.086	Cytoplasm {ECO:0000250}. Melanosome {ECO:0000250}.
Q69ZF3	Non-lysosomal glucosylceramidase	*Gba2 Kiaa1605*	-0.325	6.632	-3.377	0.014	0.155	ER
Q8K4Z3	NAD(P)H-hydrate epimerase (EC 5.1.99.6) (Apolipoprotein A-I-binding protein) (AI-BP) (NAD(P)HX epimerase)	*Naxe Aibp Apoa1bp*	-0.324	6.630	-2.791	0.030	0.228	Mitochondrion
P15105	Glutamine synthetase (GS) (EC 6.3.1.2) (Glutamate--ammonia ligase) (Palmitoyltransferase GLUL) (EC 2.3.1.225)	*Glul Glns*	-0.323	6.633	-3.867	0.007	0.114	Cytoplasm, Mitochondrion, plasma membrane, Lipid-anchor
Q91WG2	Rab GTPase-binding effector protein 2 (Rabaptin-5beta)	*Rabep2 Rabpt5b*	-0.322	6.634	-4.188	0.005	0.098	Cytoplasm, Early endosome,Cytoplasm, cytoskeleton, microtubule organizing center, centrosome, Cytoplasm, cytoskeleton, cilium basal body.
Q8BVA5	Lipid droplet-associated hydrolase (Lipid droplet-associated serine hydrolase)	*Ldah*	-0.322	6.629	-2.658	0.035	0.249	Lipid droplet
Q9EQ28	DNA polymerase delta subunit 3 (DNA polymerase delta subunit p66)	*Pold3*	-0.322	6.633	-3.896	0.007	0.113	Cytoplasm , Nucleus
P26450	Phosphatidylinositol 3-kinase regulatory subunit alpha	*Pik3r1*	-0.321	6.634	-4.290	0.004	0.093	Cytoplasm, Nucleus,golgi
O54984	ATPase GET3 (EC 3.6.-.-) (Arsenical pump-driving ATPase) (Arsenite-stimulated ATPase) (Guided entry of tail-anchored proteins factor 3, ATPase)	*Get3 Arsa Asna1*	-0.318	6.633	-3.687	0.009	0.129	Cytoplasm {ECO:0000255|HAMAP-Rule:MF_03112}. Endoplasmic reticulum {ECO:0000255|HAMAP-Rule:MF_03112}. Nucleus, nucleolus {ECO:0000255|HAMAP-Rule:MF_03112}.
Q9CQZ6	NADH dehydrogenase [ubiquinone] 1 beta subcomplex subunit 3 (Complex I-B12) (CI-B12) (NADH-ubiquinone oxidoreductase B12 subunit)	*Ndufb3*	-0.317	6.631	-2.861	0.027	0.219	Mitochondrion
Q9Z0V7	Mitochondrial import inner membrane translocase subunit Tim17-B	*Timm17b Tim17b*	-0.317	6.632	-3.197	0.017	0.174	Mitochondrion,
P28867	Protein kinase C delta type	*Prkcd Pkcd*	-0.316	6.634	-4.242	0.005	0.096	Cytoplasm, Nucleus,plasma membrane, Mitochondrion , Endosome
Q9QZ88	Vacuolar protein sorting-associated protein 29 (Vesicle protein sorting 29)	*Vps29*	-0.312	6.631	-2.993	0.022	0.198	Cytoplasm; Membrane ; Peripheral membrane protein ; Endosome membrane; Peripheral membrane protein.
A0A0B4J1H7	Immunoglobulin kappa variable 1-135	*Igkv1-135*	-0.311	6.632	-2.960	0.023	0.203	secreted
Q9R0Q9	Mannose-P-dolichol utilization defect 1 protein (Suppressor of Lec15 and Lec35 glycosylation mutation homolog) (SL15)	*Mpdu1 Supl15h*	-0.311	6.631	-2.904	0.025	0.211	plasma membrane
A0A0R4J1I6	Nucleoporin 210	*Nup210*	-0.309	6.633	-3.243	0.016	0.169	Mitochondrion,
P56391	Cytochrome c oxidase subunit 6B1 (Cytochrome c oxidase subunit VIb isoform 1) (COX VIb-1)	*Cox6b1 Cox6b*	-0.309	6.633	-3.210	0.017	0.172	Mitochondrion,
P16546	Spectrin alpha chain, non-erythrocytic 1 (Alpha-II spectrin) (Fodrin alpha chain)	*Sptan1 Spna2 Spta2*	-0.308	6.633	-3.523	0.011	0.143	Cytoplasm, cytoskeleton

Q6P1H6	Ankyrin repeat and LEM domain-containing protein 2 (LEM domain-containing protein 4)	*Ankle2 D5Ertd585e Kiaa0692 Lem4*	-0.308	6.635	-4.892	0.002	0.070	ER
Q9CQ75	NADH dehydrogenase [ubiquinone] 1 alpha subcomplex subunit 2	*Ndufa2*	-0.306	6.635	-4.263	0.005	0.094	Mitochondrion,
E9QMH1	Apolipoprotein B mRNA editing enzyme, catalytic polypeptide 3	*Apobec3*	-0.306	6.632	-2.808	0.029	0.227	Cytoplasm
A0A2K6EDP7	WD repeat and HMG-box DNA binding protein 1	*Wdhd1*	-0.304	6.632	-2.992	0.022	0.198	Nucleus ,Cytoplasm,
Q5Y5T1	Palmitoyltransferase ZDHHC20	*Zdhhc20*	-0.304	6.632	-2.978	0.023	0.200	Golgi, Cytoplasm, plasma membrane , ER
Q9CQZ5	NADH dehydrogenase [ubiquinone] 1 alpha subcomplex subunit 6 (Complex I-B14) (CI-B14) (NADH-ubiquinone oxidoreductase B14 subunit)	*Ndufa6*	-0.303	6.634	-3.549	0.011	0.142	Mitochondrion,
Q80U16	Rho family-interacting cell polarization regulator 2	*Ripor2 Fam65b Kiaa0386*	-0.300	6.635	-4.495	0.004	0.085	Cytoplasm, cytoskeleton
Q62383	Transcription elongation factor SPT6	*Supt6h Kiaa0162 Supt6*	-0.299	6.632	-3.016	0.022	0.196	Nucleus
P06339	H-2 class I histocompatibility antigen, D-37 alpha chain	*H2-T23*	-0.296	6.635	-3.798	0.008	0.118	plasma membrane
D3YXS1	Polymerase (RNA) III (DNA directed) polypeptide G like	*Polr3gl*	-0.296	6.635	-3.925	0.007	0.112	Nucleus
Q3URQ0	Testis-expressed protein 10	*Tex10*	-0.296	6.630	-2.427	0.049	0.285	Nucleus,
Q9D2M8	Ubiquitin-conjugating enzyme E2 variant 2 (Ubc-like protein MMS2)	*Ube2v2 Mms2 Uev2*	-0.296	6.634	-3.526	0.011	0.143	Nucleus, Cytoplasm, cytoskeleton
Q6PKB0	Fc fragment of IgG receptor and transporter (Fcgrt protein)	*Fcgrt*	-0.294	6.634	-3.223	0.017	0.172	plasma membrane
P45591	Cofilin-2 (Cofilin, muscle isoform)	*Cfl2*	-0.294	6.631	-2.541	0.042	0.265	Nucleus matrix. Cytoplasm, cytoskeleton.
Q60790	Ras GTPase-activating protein 3 (GAP1(IP4BP)) (GapIII) (Ins P4-binding protein)	*Rasa3*	-0.294	6.633	-2.928	0.025	0.208	plasma membrane
Q06890	Clusterin (Apolipoprotein J) (Apo-J) (Clustrin) (Sulfated glycoprotein 2) (SGP-2) [Cleaved into: Clusterin beta chain; Clusterin alpha chain]	*Clu Apoj Msgp-2*	-0.293	6.632	-2.877	0.026	0.215	Secreted,. Nucleus ,Cytoplasm, Mitochondrion membrane,ER
P97762	Retinitis pigmentosa 9 protein homolog (Pim-1-associated protein) (PAP-1)	*rp9 Rp9h*	-0.293	6.632	-2.549	0.041	0.264	Nucleus {ECO:0000305}.
Q6ZPL9	ATP-dependent RNA helicase DDX55 (EC 3.6.4.13) (DEAD box protein 55)	*Ddx55 Kiaa1595*	-0.292	6.636	-4.290	0.004	0.093	Nucleus ,Cytoplasm,
Q60611	DNA-binding protein SATB1 (Special AT-rich sequence-binding protein 1)	*Satb1*	-0.292	6.635	-3.627	0.010	0.136	Nucleus.
Q9CPN7	Protein transport protein Sec24A (SEC24-related protein A)	*Sec24a*	-0.291	6.634	-3.232	0.016	0.171	plasma membrane
Q3U2P1	Phosphatidylinositol 4-kinase alpha (PI4-kinase alpha) (PI4K-alpha) (PtdIns-4-kinase alpha) (EC 2.7.1.67)	*Pi4ka Pik4 Pik4ca*	-0.290	6.636	-3.787	0.008	0.119	Cytoplasm {ECO:0000250|UniProtKB:P42356}. Cell membrane {ECO:0000250|UniProtKB:P42356}.
Q6VYH9	Hematopoietic SH2 domain-containing protein (Hematopoietic SH2 protein) (Adaptor in lymphocytes of unknown function X)	*Hsh2d Alx*	-0.288	6.632	-2.559	0.041	0.263	Cytoplasm {ECO:0000269|PubMed:15569688}. Mitochondrion {ECO:0000269|PubMed:15569688}.
A0A075B5V0	Immunoglobulin heavy variable 1-26	*Ighv1-26*	-0.288	6.633	-2.843	0.027	0.222	secreted
E9Q7G1	Transmembrane p24 trafficking protein 7	*Tmed7*	-0.288	6.633	-2.696	0.034	0.243	Endoplasmic reticulum membrane ,Single-pass type I membrane protein , Endoplasmic reticulum-Golgi intermediate compartment membrane ,Golgi apparatus, Membrane.
Q5SUR0	Phosphoribosylformylglycinamidine synthase	*Pfas Kiaa0361*	-0.287	6.635	-3.546	0.011	0.142	Cytoplasm {ECO:0000250}.
P26011	Integrin beta-7 (Integrin beta-P) (M290 IEL antigen)	*Itgb7*	-0.285	6.632	-2.436	0.048	0.284	SCell membrane {ECO:0000305}; Single-pass type I membrane protein {ECO:0000255}.

E9QP62	LIM and senescent cell antigen-like-containing domain protein	*Lims1*	-0.282	6.634	-2.855	0.027	0.219	Cell junction, focal adhesion {ECO:0000256|PIRNR:PIRNR038003}. Cell membrane {ECO:0000256|PIRNR:PIRNR038003}; Peripheral membrane protein {ECO:0000256|PIRNR:PIRNR038003}.
F8VQE2	Kinesin family member 21B	*Kif21b*	-0.281	6.633	-2.668	0.035	0.247	Cytoplasm,
A0A0G2JEC2	Myocyte enhancer factor 2C	*Mef2c*	-0.281	6.637	-4.169	0.005	0.098	Nucleus
Q60770	Syntaxin-binding protein 3 (MUNC-18-3) (Mammalian homolog of Unc-18c) (Munc-18c) (Protein unc-18 homolog 3) (Unc18-3) (Protein unc-18 homolog C) (Unc-18C)	*Stxbp3 Stxbp3a Unc18c*	-0.281	6.635	-3.034	0.021	0.194	Cytoplasm, plasma membrane
Q9DBG3	AP-2 complex subunit beta	*Ap2b1 Clapb1*	-0.280	6.632	-2.437	0.048	0.284	plasma membrane
Q99KN1	Arrestin domain-containing protein 1 (Alpha-arrestin 1)	*Arrdc1*	-0.279	6.635	-3.151	0.018	0.178	plasma membrane
A0A0R4IZY0	Thimet oligopeptidase 1	*Thop1*	-0.277	6.636	-3.793	0.008	0.119	Cytoplasm,
Q62261	Spectrin beta chain, non-erythrocytic 1 (Beta-II spectrin) (Embryonic liver fodrin) (Fodrin beta chain)	*Sptbn1 Elf Spnb-2 Spnb2 Sptb2*	-0.277	6.636	-3.622	0.010	0.136	Cytoplasm, cytoskeleton plasma membrane
Q99N57	RAF proto-oncogene serine/threonine-protein kinase (EC 2.7.11.1) (Proto-oncogene c-RAF) (cRaf) (Raf-1)	*Raf1 Craf*	-0.276	6.635	-3.377	0.014	0.155	Cytoplasm,plasma membrane ,Mitochondrion, Nucleus
P00375	Dihydrofolate reductase (EC 1.5.1.3)	*Dhfr*	-0.274	6.633	-2.615	0.038	0.255	Mitochondrion {ECO:0000269|PubMed:25980602}. Cytoplasm {ECO:0000269|PubMed:25980602}.
Q9JK81	MYG1 exonuclease (EC 3.1.-.-) (Protein Gamm1)	*Myg1*	-0.273	6.634	-2.617	0.037	0.255	Nucleus, plasma membrane ,Mitochondrion ,Nucleus
Q5UE59	Kinesin light chain	*Klc1 Kns2*	-0.273	6.637	-3.813	0.008	0.117	Cytoplasm, cytoskeleton
Q9DCT2	NADH dehydrogenase [ubiquinone] iron-sulfur protein 3, mitochondrial (EC 7.1.1.2) (Complex I-30kD) (CI-30kD) (NADH-ubiquinone oxidoreductase 30 kDa subunit)	*Ndufs3*	-0.272	6.637	-3.718	0.009	0.127	Mitochondrion
A0A0R4J0H7	Condensin complex subunit 1	*Ncapd2*	-0.270	6.633	-2.670	0.035	0.247	Nucleus
F8WIE5	E3 ubiquitin-protein ligase (EC 2.3.2.26)	*Hectd1*	-0.270	6.635	-2.835	0.028	0.224	Nucleus
Q60749	KH domain-containing, RNA-binding, signal transduction-associated protein 1 (GAP-associated tyrosine phosphoprotein p62) (Src-associated in mitosis 68 kDa protein) (Sam68) (p21 Ras GTPase-activating protein-associated p62) (p68)	*Khdrbs1*	-0.269	6.636	-3.553	0.011	0.142	Nucleus,Cytoplasm,plasma membrane
A0A075B5V6	Immunoglobulin heavy variable V1-42	*Ighv1-42*	-0.268	6.635	-2.824	0.028	0.225	secreted
Q924M7	Mannose-6-phosphate isomerase (EC 5.3.1.8) (Phosphohexomutase) (Phosphomannose isomerase) (PMI)	*Mpi Mpi1 Pmi*	-0.266	6.634	-2.502	0.044	0.272	Cytoplasm {ECO:0000269|PubMed:12122025}.
Q9D0R4	Probable ATP-dependent RNA helicase DDX56 (EC 3.6.4.13) (ATP-dependent 61 kDa nucleolar RNA helicase) (DEAD box protein 56)	*Ddx56 D11Ertd619e Noh61*	-0.265	6.637	-3.557	0.011	0.142	Nucleus, nucleolus {ECO:0000269|PubMed:32703285}.
Q9CZ19	Myosin, light polypeptide 4	*Myl4*	-0.265	6.636	-3.419	0.013	0.153	Cytoplasm,
A0A1W2P7U1	Cell division cycle 34	*Cdc34*	-0.264	6.635	-2.677	0.035	0.246	Cytoplasm, Nucleus
E9Q555	E3 ubiquitin-protein ligase RNF213 (EC 2.3.2.27) (EC 3.6.4.-) (E3 ubiquitin-lipopolysaccharide ligase RNF213) (EC 2.3.2.-) (Mysterin) (RING finger protein 213)	*Rnf213 Mystr*	-0.263	6.637	-3.429	0.013	0.152	Cytoplasm, ,Lipid droplet
Q8CEC6	Peptidylprolyl isomerase domain and WD repeat-containing protein 1 (EC 5.2.1.8)	*Ppwd1*	-0.263	6.636	-3.001	0.022	0.197	Nucleus

Q9D8T2	Gasdermin-D (Gasdermin domain-containing protein 1) [Cleaved into: Gasdermin-D, N-terminal (GSDMD-NT) (mGSDMD-NTD) (p30); Gasdermin-D, C-terminal (GSDMD-CT) (mGSDMD-CTD) (p20); Gasdermin-D, p13 (Gasdermin-D, 13 kDa) (13 kDa GSDMD); Gasdermin-D, p40]	*Gsdmd Gsdmdc1*	-0.262	6.637	-3.445	0.012	0.150	Cytoplasm, Nucleus
Q9JL60	Glucocorticoid modulatory element-binding protein 1 (GMEB-1)	*Gmeb1*	-0.262	6.634	-2.629	0.037	0.254	Nucleus. Cytoplasm.
P48410	ATP-binding cassette sub-family D member 1 (EC 3.1.2.-) (EC 7.6.2.-) (Adrenoleukodystrophy protein) (ALDP)	*Abcd1 Ald Aldgh*	-0.261	6.634	-2.417	0.050	0.286	Peroxisome membrane ,plasma membrane ,Lysosome membrane
A0A0R4J050	N-acyl-aliphatic-L-amino acid amidohydrolase (EC 3.5.1.14) (N-acyl-L-amino-acid amidohydrolase)	*Acy1*	-0.261	6.636	-2.960	0.023	0.203	Cytoplasm
Q99KB8	Hydroxyacylglutathione hydrolase, mitochondrial (EC 3.1.2.6) (Glyoxalase II) (Glx II)	*Hagh Glo2*	-0.259	6.635	-2.791	0.030	0.228	Mitochondrion, Cytoplasm {ECO:0000250|UniProtKB:Q16775}.
Q7TSG2	RNA polymerase II subunit A C-terminal domain phosphatase (EC 3.1.3.16) (TFIIF-associating CTD phosphatase)	*Ctdp1 Fcp1*	-0.259	6.636	-2.999	0.022	0.198	Nucleus {ECO:0000250}. Cytoplasm, cytoskeleton
Q9DBR0	A-kinase anchor protein 8 (AKAP-8) (A-kinase anchor protein 95 kDa) (AKAP 95)	*Akap8 Akap95*	-0.256	6.636	-2.934	0.024	0.208	Nucleus ,Cytoplasm
Q9EQP2	EH domain-containing protein 4 (PAST homolog 2) (mPAST2)	*Ehd4 Past2*	-0.255	6.637	-3.518	0.011	0.143	Early endosome membrane, plasma membrane
Q61161	Mitogen-activated protein kinase kinase kinase kinase 2 (EC 2.7.11.1) (Germinal center kinase) (GCK) (MAPK/ERK kinase kinase kinase 2) (MEK kinase kinase 2) (MEKKK 2) (Rab8-interacting protein)	*Map4k2 Rab8ip*	-0.254	6.636	-3.019	0.022	0.196	Cytoplasm,plasma membrane
E9PY48	Condensin-2 complex subunit H2 (Non-SMC condensin II complex subunit H2)	*Ncaph2*	-0.252	6.636	-2.652	0.036	0.250	Nucleus
P82343	N-acylglucosamine 2-epimerase (AGE) (EC 5.1.3.8) (GlcNAc 2-epimerase) (N-acetyl-D-glucosamine 2-epimerase) (Renin-binding protein) (RnBP)	*Renbp*	-0.251	6.636	-2.697	0.034	0.243	Cytoplasm,exosome
A0A1Y7VNC5	Ninein	*Nin*	-0.251	6.635	-2.518	0.043	0.269	Cytoplasm, , cytoskeleton.
A2A7K5	MYC binding protein	*Mycbp*	-0.249	6.635	-2.468	0.046	0.278	Nucleus {ECO:0000256|ARBA:ARBA00004123}.
Q9QZB7	Actin-related protein 10 (Actin-related protein 11)	*Actr10 Act11 Actr11 Arp10 Arp11*	-0.249	6.636	-2.807	0.029	0.227	Cytoplasm, cytoskeleton {ECO:0000250|UniProtKB:I3LHK5}.
Q8C2K1	Differentially expressed in FDCP 6 (DEF-6) (IRF4-binding protein) (SWAP-70-like adapter of T-cells)	*Def6 Ibp Slat*	-0.247	6.638	-3.417	0.013	0.153	Cytoplasm plasma membrane
G3X9H5	Huntingtin	*Htt*	-0.246	6.639	-3.869	0.007	0.114	Cytoplasm,Nucleus
Q3UVL4	Vacuolar protein sorting-associated protein 51 homolog (Protein fat-free homolog)	*Vps51 Ffr*	-0.246	6.636	-2.677	0.035	0.246	Golgi apparatus,endosome
P25911	Tyrosine-protein kinase Lyn (EC 2.7.10.2) (V-yes-1 Yamaguchi sarcoma viral related oncogene homolog) (p53Lyn) (p56Lyn)	*Lyn*	-0.244	6.635	-2.426	0.049	0.285	plasma membrane, Nucleus
D3YU12	NmrA-like family domain containing 1	*Nmral1*	-0.243	6.637	-3.186	0.017	0.175	Cytoplasm, Nucleus
Q3U4X8	DNA ligase (EC 6.5.1.1)	*Lig1*	-0.243	6.637	-2.778	0.030	0.230	plasma membrane
Q3TZX8	Polynucleotide 5'-hydroxyl-kinase NOL9 (EC 2.7.1.-) (Nucleolar protein 9)	*Nol9*	-0.241	6.638	-3.864	0.007	0.114	Nucleus,
Q3U3K9	Lipid droplet-regulating VLDL assembly factor AUP1 (Ancient ubiquitous protein 1)	*Aup1*	-0.241	6.635	-2.515	0.043	0.269	plasma membrane

Q9D517	1-acyl-sn-glycerol-3-phosphate acyltransferase gamma (EC 2.3.1.51) (1-acylglycerol-3-phosphate O-acyltransferase 3) (1-AGP acyltransferase 3) (1-AGPAT 3) (Lysophosphatidic acid acyltransferase gamma) (LPAAT-gamma)	*Agpat3 Lpaat3*	-0.241	6.638	-3.501	0.012	0.144	ER
Q9WTI7	Unconventional myosin-Ic (Myosin I beta) (MMI-beta) (MMIb)	*Myo1c*	-0.241	6.636	-2.628	0.037	0.254	Cytoplasm ,Nucleus
Q3UHC1	Rap guanine nucleotide exchange factor (GEF) 1	*Rapgef1*	-0.240	6.637	-2.883	0.026	0.215	Cytoplasm,endosome
Q9DC70	NADH dehydrogenase [ubiquinone] iron-sulfur protein 7, mitochondrial (EC 7.1.1.2) (Complex I-20kD) (CI-20kD) (NADH-ubiquinone oxidoreductase 20 kDa subunit)	*Ndufs7*	-0.239	6.638	-3.061	0.021	0.191	Mitochondrion,
F8WJ93	Echinoderm microtubule associated protein like 4	*Eml4*	-0.238	6.639	-3.609	0.010	0.138	Cytoplasm, cytoskeleton
A2AB59	Rho GTPase-activating protein 27 (CIN85-associated multi-domain-containing Rho GTPase-activating protein 1) (Rho-type GTPase-activating protein 27)	*Arhgap27 Camgap1*	-0.238	6.637	-2.924	0.025	0.208	Cytoplasm , cytoplasm Membrane
P42230	Signal transducer and activator of transcription 5A (Mammary gland factor)	*Stat5a Mgf Mpf*	-0.237	6.638	-3.317	0.015	0.162	Cytoplasm ,Nucleus
Q8CCB4	Vacuolar protein sorting-associated protein 53 homolog	*Vps53*	-0.236	6.638	-2.928	0.025	0.208	Golgi ,Recycling endosome
Q9JJA4	Ribosome biogenesis protein WDR12 (WD repeat-containing protein 12)	*Wdr12 MNCb-5414*	-0.236	6.637	-3.019	0.022	0.196	Nucleus,
P97822	Acidic leucine-rich nuclear phosphoprotein 32 family member E (Cerebellar postnatal development protein 1) (LANP-like protein) (LANP-L)	*Anp32e Cpd1*	-0.235	6.638	-3.086	0.020	0.187	Cytoplasm. Nucleus.
A0A0R4J0R3	Interferon stimulated exonuclease gene 20-like 2	*Isg20l2*	-0.233	6.639	-3.243	0.016	0.169	Nucleus
Q8BHN3	Neutral alpha-glucosidase AB (EC 3.2.1.207) (Alpha-glucosidase 2) (Glucosidase II subunit alpha)	*Ganab G2an Kiaa0088*	-0.232	6.637	-2.827	0.028	0.225	Endoplasmic reticulum, Golgi apparatus, Melanosome .
O88199	Carbohydrate sulfotransferase 3 (EC 2.8.2.17) (EC 2.8.2.21) (Chondroitin 6-O-sulfotransferase 1) (C6ST-1) (Chondroitin 6-sulfotransferase) (C6ST) (Galactose/N-acetylglucosamine/N-acetylglucosamine 6-O-sulfotransferase 0) (GST-0)	*Chst3 C6st Gst0*	-0.232	6.637	-2.739	0.032	0.236	Golgi apparatus membrane {ECO:0000250}; Single-pass type II membrane protein {ECO:0000250}.
V9GXM1	ADP-ribosylation factor GTPase activating protein 1	*Arfgap1*	-0.232	6.636	-2.441	0.048	0.284	Cytoplasm.golgi
P98083	SHC-transforming protein 1 (SHC-transforming protein A) (Src homology 2 domain-containing-transforming protein C1) (SH2 domain protein C1)	*Shc1 Shc ShcA*	-0.232	6.638	-3.271	0.016	0.168	Cytoplasm ,Mitochondrion matrix,
A0A0A0MQ99	DNA repair protein SWI5 homolog (Protein SAE3 homolog)	*Swi5*	-0.232	6.638	-3.189	0.017	0.175	Cytoplasm
Q8VCW8	Medium-chain acyl-CoA ligase ACSF2, mitochondrial (EC 6.2.1.2)	*Acsf2*	-0.232	6.638	-2.880	0.026	0.215	Mitochondrion {ECO:0000305}.
P48722	Heat shock 70 kDa protein 4L (Heat shock 70-related protein APG-1) (Osmotic stress protein 94)	*Hspa4l Apg1 Hsp4l Osp94*	-0.231	6.639	-3.603	0.010	0.138	Cytoplasm {ECO:0000250}. Nucleus {ECO:0000250}. Note=May translocate to the nucleus after heat shock. {ECO:0000250}.
A7UAK5	6-phosphofructo-2-kinase/fructose-2, 6-biphosphatase 3 splice variant 2 (EC 2.7.1.105, EC 3.1.3.46) (6-phosphofructo-2-kinase/fructose-2,6-biphosphatase 3)	*Pfkfb3*	-0.230	6.639	-3.372	0.014	0.156	Cytoplasm
Q3U6B2	G-protein coupled receptor 183 (Epstein-Barr virus-induced G-protein coupled receptor 2 homolog) (EBI2) (EBV-induced G-protein coupled receptor 2 homolog)	*Gpr183 Ebi2*	-0.229	6.637	-2.537	0.042	0.266	cytoplasm membrane

Q8CG47	Structural maintenance of chromosomes protein 4 (SMC protein 4) (SMC-4) (Chromosome-associated polypeptide C) (XCAP-C homolog)	*Smc4 Capc Smc4l1*	-0.229	6.637	-2.601	0.038	0.256	Nucleus {ECO:0000250}. Cytoplasm
P83877	Thioredoxin-like protein 4A (DIM1 protein homolog) (Spliceosomal U5 snRNP-specific 15 kDa protein) (Thioredoxin-like U5 snRNP protein U5-15kD)	*Txnl4a Dim1 Txnl4*	-0.228	6.637	-2.856	0.027	0.219	Nucleus
Q9Z1N5	Spliceosome RNA helicase Ddx39b (EC 3.6.4.13) (56 kDa U2AF65-associated protein) (DEAD box protein UAP56) (HLA-B-associated transcript 1 protein)	*Ddx39b Bat1 Bat1a Uap56*	-0.228	6.638	-2.816	0.029	0.226	Nucleus ,Cytoplasm
Q61699	Heat shock protein 105 kDa (42 degrees C-HSP) (Heat shock 110 kDa protein) (Heat shock-related 100 kDa protein E7I) (HSP-E7I)	*Hsph1 Hsp105 Hsp110 Kiaa0201*	-0.227	6.639	-3.184	0.017	0.175	Cytoplasm,Nucleus
Q3TPX4	Exocyst complex component 5 (Exocyst complex component Sec10)	*Exoc5 Sec10l1*	-0.227	6.639	-3.263	0.016	0.168	Cytoplasm
Q9JL62	Glycolipid transfer protein (GLTP)	*Gltp*	-0.224	6.638	-2.805	0.029	0.227	Cytoplasm {ECO:0000250}.
Q09200	Beta-1,4 N-acetylgalactosaminyltransferase 1 (EC 2.4.1.92) ((N-acetylneuraminyl)-galactosylglucosylceramide) (GM2/GD2 synthase) (GalNAc-T)	*B4galnt1 Galgt Galgt1 Ggm2*	-0.222	6.639	-3.321	0.015	0.162	Golgi apparatus membrane
Q91V89	Serine/threonine-protein phosphatase 2A 56 kDa regulatory subunit	*Ppp2r5d*	-0.222	6.637	-2.434	0.048	0.284	Cytoplasm ,Nucleus ,PLasma membrane
Q9EQG9	Ceramide transfer protein (CERT) (Collagen type IV alpha-3-binding protein) (Goodpasture antigen-binding protein) (GPBP) (START domain-containing protein 11) (StARD11) (StAR-related lipid transfer protein 11)	*Cert1 Cert Col4a3bp Stard11*	-0.220	6.640	-3.394	0.013	0.154	Cytoplasm ,Golgi apparatus, ER
Q5SVG5	AP complex subunit beta	*Ap1b1*	-0.220	6.638	-2.796	0.029	0.227	golgi, plasma membrane
O89053	Coronin-1A (Coronin-like protein A) (Clipin-A) (Coronin-like protein p57) (Tryptophan aspartate-containing coat protein) (TACO)	*Coro1a Coro1*	-0.220	6.639	-3.134	0.019	0.179	Cytoplasm, cytoskeleton
Q6GV12	3-ketodihydrosphingosine reductase (KDS reductase) (EC 1.1.1.102) (3-dehydrosphinganine reductase) (Follicular variant translocation protein 1 homolog) (FVT-1)	*Kdsr Fvt1*	-0.220	6.638	-2.678	0.034	0.246	ER
A3KMP2	Tetratricopeptide repeat protein 38 (TPR repeat protein 38)	*Ttc38*	-0.218	6.639	-2.803	0.029	0.227	exosome
P36552	Oxygen-dependent coproporphyrinogen-III oxidase, mitochondrial (COX) (Coprogen oxidase) (Coproporphyrinogenase) (EC 1.3.3.3)	*Cpox Cpo*	-0.216	6.639	-3.230	0.016	0.171	Mitochondrion,
Q9D142	Uridine diphosphate glucose pyrophosphatase NUDT14 (UDPG pyrophosphatase) (UGPPase) (EC 3.6.1.45) (Nucleoside diphosphate-linked moiety X motif 14) (Nudix motif 14)	*Nudt14*	-0.214	6.638	-2.781	0.030	0.229	Cytoplasm
Q80ZD3	Sodium-independent sulfate anion transporter (Kidney brain anion transporter) (KBAT) (Solute carrier family 26 member 11)	*Slc26a11*	-0.212	6.638	-2.546	0.041	0.265	plasma membrane
Q3UW64	Bifunctional UDP-N-acetylglucosamine 2-epimerase/N-acetylmannosamine kinase	*Gne*	-0.212	6.639	-3.057	0.021	0.191	Cytoplasm
P41241	Tyrosine-protein kinase CSK (EC 2.7.10.2) (C-Src kinase) (Protein-tyrosine kinase MPK-2) (p50CSK)	*Csk*	-0.211	6.640	-3.212	0.017	0.172	plasma membrane
Q3UHD6	Sorting nexin-27	*Snx27 Kiaa0488*	-0.211	6.639	-2.998	0.022	0.198	Early endosome membrane,Cytoplasm, plasma membrane
Q3TBD2	Rho GTPase-activating protein 45 (Minor histocompatibility protein HA-1)	*Arhgap45 Hmha1*	-0.211	6.638	-2.522	0.043	0.268	Cytoplasm
Q6Q899	Antiviral innate immune response receptor RIG-I	*Rigi Ddx58*	-0.209	6.640	-3.072	0.020	0.189	Cytoplasm, cytoskeleton
B1AXN9	non-specific serine/threonine protein kinase (EC 2.7.11.1)	*Rps6ka3*	-0.209	6.639	-2.556	0.041	0.263	cell membrane

P63280	SUMO-conjugating enzyme UBC9	*Ube2i Ubc9 Ubce2i Ubce9*	-0.209	6.639	-2.608	0.038	0.256	Nucleus , Cytoplasm
H3BLL2	ATP synthase mitochondrial F1 complex assembly factor 1	*Atpaf1*	-0.208	6.639	-2.566	0.040	0.262	Mitochondrion
Q9DBR7	Protein phosphatase 1 regulatory subunit 12A	*Ppp1r12a Mypt1*	-0.207	6.640	-3.405	0.013	0.153	Cytoplasm. Cytoskeleton
O88597	Beclin-1 (Coiled-coil myosin-like BCL2-interacting protein) [Cleaved into: Beclin-1-C 35 kDa; Beclin-1-C 37 kDa]	*Becn1*	-0.206	6.639	-2.488	0.045	0.274	Cytoplasm,Golgi apparatus
G3UWZ0	Bromodomain adjacent to zinc finger domain 1A	*Baz1a*	-0.205	6.640	-3.196	0.017	0.174	Nucleus
Q91V35	Receptor-type tyrosine-protein phosphatase alpha (EC 3.1.3.48)	*Ptpra*	-0.204	6.639	-2.627	0.037	0.254	plasma membrane
Q9CQM9	Glutaredoxin-3 (PKC-interacting cousin of thioredoxin) (PICOT) (PKC-theta-interacting protein) (PKCq-interacting protein) (Thioredoxin-like protein 2)	*Glrx3 Picot Txnl2*	-0.203	6.639	-2.588	0.039	0.258	Cytoplasm, ,Endosome membrane
Q9EQ61	Pescadillo homolog	*Pes1 Pes*	-0.198	6.639	-2.494	0.045	0.273	Nucleus
Q9DCA5	Ribosome biogenesis protein BRX1 homolog (Brix domain-containing protein 2)	*Brix1 Brix Bxdc2*	-0.196	6.640	-2.733	0.032	0.236	Nucleus
Q923S9	Ras-related protein Rab-30	*Rab30 Rsb30*	-0.195	6.639	-2.421	0.049	0.286	Plasma Membrane {ECO:0000250}; Lipid-anchor,Golgi apparatus,
Q9JKW0	ADP-ribosylation factor-like protein 6-interacting protein 1 (ARL-6-interacting protein 1) (Aip-1) (Protein TBX2)	*Arl6ip1 Arl6ip*	-0.195	6.640	-2.809	0.029	0.227	Endosome membrane
Q9CPQ1	Cytochrome c oxidase subunit 6C (Cytochrome c oxidase polypeptide VIc)	*Cox6c*	-0.194	6.640	-2.917	0.025	0.209	Mitochondrion
P17426	AP-2 complex subunit alpha-1	*Ap2a1 Adtaa Clapa1*	-0.192	6.640	-2.729	0.032	0.237	plasma membrane
S0DHL8	Capping protein regulator and myosin 1 linker 2 (RLTPR)	*Carmil2 Rltpr*	-0.192	6.640	-2.826	0.028	0.225	Cytoplasm
Q6PD03	Serine/threonine-protein phosphatase 2A 56 kDa regulatory subunit alpha isoform	*Ppp2r5a*	-0.190	6.640	-2.547	0.041	0.264	Cytoplasm ,Nucleus
Q9DB77	Cytochrome b-c1 complex subunit 2, mitochondrial	*Uqcrc2*	-0.190	6.640	-2.615	0.038	0.255	Mitochondrion,
P08207	Protein S100-A10	*S100a10 Cal1l*	-0.189	6.639	-2.432	0.049	0.284	Cytoplasm,plasma membrane
O70145	Neutrophil cytosol factor 2 (NCF-2) (67 kDa neutrophil oxidase factor) (NADPH oxidase activator 2) (Neutrophil NADPH oxidase factor 2) (p67-phox)	*Ncf2 Noxa2 P67phox*	-0.188	6.640	-2.737	0.032	0.236	Cytoplasm
Q8K1M6	Dynamin-1-like protein (EC 3.6.5.5) (Dynamin family member proline-rich carboxyl-terminal domain less) (Dymple) (Dynamin-related protein 1)	*Dnm1l Drp1*	-0.185	6.640	-2.740	0.032	0.236	Cytoplasm, ,Golgi apparatus {ECO:0000250}. Endomembrane system;Mitochondrion,
P97807	Fumarate hydratase, mitochondrial (Fumarase) (EC 4.2.1.2) (EF-3)	*Fh Fh1*	-0.184	6.640	-2.625	0.037	0.254	Mitochondrion,
Q9DBG7	Signal recognition particle receptor subunit alpha (SR-alpha) (Docking protein alpha) (DP-alpha)	*Srpra Srpr*	-0.184	6.640	-2.559	0.041	0.263	Endoplasmic reticulum membrane
O35943	Frataxin, mitochondrial	*Fxn Frda*	-0.182	6.640	-2.644	0.036	0.252	Mitochondrion,
Q9R0Q6	Actin-related protein 2/3 complex subunit 1A (SOP2-like protein) (Sid 329)	*Arpc1a Sid329*	-0.182	6.640	-2.492	0.045	0.273	Cytoplasm, cytoskeleton,Nucleus.
Q8BFY9	Transportin-1 (Importin beta-2) (Karyopherin beta-2)	*Tnpo1 Kpnb2*	-0.180	6.640	-2.534	0.042	0.266	Cytoplasm, Nucleus .
E9Q8N5	CLIP associating protein 2	*Clasp2*	-0.179	6.641	-2.420	0.049	0.286	Cytoplasm,plasma membrane, golgi,cytoskeleton
P35564	Calnexin	*Canx*	-0.179	6.640	-2.513	0.043	0.269	ER
Q9JKY5	Huntingtin-interacting protein 1-related protein (HIP1-related protein)	*Hip1r*	-0.179	6.641	-2.626	0.037	0.254	Cytoplasm
E9PUF7	Rho guanine nucleotide exchange factor (GEF) 1	*Arhgef1*	-0.176	6.641	-2.689	0.034	0.244	plasma membrane
Q3UMB9	WASH complex subunit 4 (WASH complex subunit SWIP)	*Washc4 Kiaa1033*	-0.176	6.641	-2.754	0.031	0.234	Early endosome

Q9DCR2	AP-3 complex subunit sigma-1	*Ap3s1*	-0.173	6.641	-2.469	0.046	0.278	Golgi apparatus.plasma membrane
Q8BWZ3	N-alpha-acetyltransferase 25, NatB auxiliary subunit	*Naa25 Mdm20*	-0.172	6.641	-2.711	0.033	0.241	Cytoplasm
E9Q9M1	5'-nucleotidase, cytosolic II	*Nt5c2*	-0.169	6.641	-2.438	0.048	0.284	Cytoplasm
P61164	Alpha-centractin (Centractin) (ARP1) (Actin-RPV) (Centrosome-associated actin homolog)	*Actr1a Ctrn1*	-0.166	6.641	-2.472	0.046	0.277	Cytoplasm, cytoskeleton.
A0A0R4J215	G patch domain-containing protein 11 (Coiled-coil domain-containing protein 75)	*Gpatch11*	-0.166	6.641	-2.620	0.037	0.255	Nucleus
Q6PGB6	N-alpha-acetyltransferase 50 (EC 2.3.1.258) (N-acetyltransferase NAT13) (N-epsilon-acetyltransferase 50) (EC 2.3.1.-) (NatE catalytic subunit)	*Naa50 Mak3 Nat13*	-0.165	6.641	-2.441	0.048	0.284	Cytoplasm, Nucleus,plasma membrane, Mitochondrion , Endosome
O35286	ATP-dependent RNA helicase DHX15 (EC 3.6.4.13) (DEAH box protein 15)	*Dhx15 Ddx15 Deah9*	-0.165	6.641	-2.414	0.050	0.287	Nucleus
Q3THK3	General transcription factor IIF subunit 1 (Transcription initiation factor IIF subunit alpha) (TFIIF-alpha)	*Gtf2f1*	-0.160	6.641	-2.477	0.046	0.277	Nucleus
P06800	Receptor-type tyrosine-protein phosphatase C (EC 3.1.3.48) (Leukocyte common antigen) (L-CA) (Lymphocyte antigen 5) (Ly-5) (T200) (CD antigen CD45)	*Ptprc Ly-5*	-0.160	6.642	-2.582	0.039	0.259	plasma membrane
E9Q586	Dynactin subunit 1	*Dctn1*	-0.156	6.641	-2.428	0.049	0.284	Cytoplasm,cytoskeleton
Q9DCJ7	Small ribosomal subunit protein mS38	*Aurkaip1 Aip Akip Mrps38*	0.500	6.618	4.565	0.003	0.083	Mitochondrion matrix {ECO:0000250|UniProtKB:Q9NWT8}. Nucleus {ECO:0000250|UniProtKB:Q9NWT8}.
P51827	AF4/FMR2 family member 3 (Lymphoid nuclear protein related to AF4) (Protein LAF-4)	*Aff3 Laf4*	0.501	6.599	2.505	0.044	0.271	Nucleus.
O88425	Nucleoside diphosphate kinase 6 (NDK 6) (NDP kinase 6) (EC 2.7.4.6) (nm23-M6)	*Nme6*	0.501	6.610	3.182	0.017	0.175	Mitochondrion
Q8BUV8	Protein GPR107	*Gpr107 Kiaa1624*	0.502	6.615	3.676	0.009	0.130	Cell membrane; Multi-pass membrane protein; Golgi apparatus, trans-Golgi network membrane; Multi-pass membrane protein.
Q8BSF4	Phosphatidylserine decarboxylase proenzyme, mitochondrial (EC 4.1.1.65) [Cleaved into: Phosphatidylserine decarboxylase beta chain; Phosphatidylserine decarboxylase alpha chain]	*Pisd*	0.505	6.618	4.957	0.002	0.067	Mitochondrion inner membrane ; Mitochondrion inner membrane , Lipid droplet
Q99N91	Large ribosomal subunit protein bL34m (39S ribosomal protein L34, mitochondrial) (L34mt) (MRP-L34)	*Mrpl34*	0.507	6.613	3.410	0.013	0.153	Mitochondrion
Q6PE15	Palmitoyl-protein thioesterase ABHD10, mitochondrial	*Abhd10*	0.509	6.616	4.310	0.004	0.093	Mitochondrion
Q80YV4	4'-phosphopantetheine phosphatase (EC 3.1.3.-) (Inactive pantothenic acid kinase 4) (mPanK4)	*Pank4*	0.517	6.618	5.662	0.001	0.048	Cytoplasm
P17095	High mobility group protein HMG-I/HMG-Y (HMG-I(Y)) (High mobility group AT-hook protein 1) (High mobility group protein A1)	*Hmga1 Hmgi Hmgiy*	0.517	6.609	3.168	0.018	0.176	Nucleus. Chromosome.
D3YX27	HtrA serine peptidase 2	*Htra2*	0.518	6.608	2.967	0.023	0.202	Mitochondrion membrane
P85094	Isochorismatase domain-containing protein 2A	*Isoc2a Isoc2*	0.518	6.593	2.442	0.048	0.284	Cytoplasm {ECO:0000250}. Nucleus {ECO:0000250}.
Q8R035	Large ribosomal subunit protein mL62	*Mrpl58 Ict1*	0.520	6.619	6.628	0.000	0.034	Mitochondrion
P18608	Non-histone chromosomal protein HMG-14 (High mobility group nucleosome-binding domain-containing protein 1)	*Hmgn1 Hmg-14 Hmg14*	0.520	6.600	2.612	0.038	0.255	Nucleus. Cytoplasm.

P83917	Chromobox protein homolog 1 (Heterochromatin protein 1 homolog beta) (HP1 beta) (Heterochromatin protein p25) (M31) (Modifier 1 protein)	*Cbx1 Cbx*	0.521	6.606	2.958	0.024	0.203	Nucleus
A0A0R4J0B2	Phospholipase B-like (EC 3.1.1.-)	*Plbd1*	0.527	6.605	3.158	0.018	0.178	lysosome
Q9Z0L8	Gamma-glutamyl hydrolase (EC 3.4.19.9) (Conjugase) (FGPH) (Folylpolyglutamate hydrolase) (GH) (Gamma-Glu-x carboxypeptidase)	*Ggh*	0.531	6.606	3.009	0.022	0.197	Secreted, extracellular space; Lysosome ; Melanosome.
F2Z3U6	11-beta-hydroxysteroid dehydrogenase 1 (EC 1.1.1.146) (EC 1.1.1.201) (7-oxosteroid reductase) (Corticosteroid 11-beta-dehydrogenase isozyme 1)	*Hsd11b1*	0.534	6.596	2.555	0.041	0.263	Endoplasmic reticulum membrane .
P48758	Carbonyl reductase [NADPH] 1	*Cbr1 Cbr*	0.535	6.602	3.069	0.020	0.189	Cytoplasm
Q67FY2	B-cell CLL/lymphoma 9-like protein (B-cell lymphoma 9-like protein) (BCL9-like protein) (BCL9-related beta-catenin-binding protein) (Protein BCL9-2)	*Bcl9l B9l*	0.536	6.612	4.512	0.003	0.085	Nucleus
Q9CPV3	Large ribosomal subunit protein mL42	*Mrpl42 D10Ertd322e Mrps32*	0.537	6.609	3.409	0.013	0.153	Mitochondrion
Q9DC16	Endoplasmic reticulum-Golgi intermediate compartment protein 1 (ER-Golgi intermediate compartment 32 kDa protein) (ERGIC-32)	*Ergic1 Ergic32*	0.538	6.614	5.134	0.002	0.061	Endoplasmic reticulum membrane ; Multi-pass membrane protein; Golgi apparatus membrane.
Q61391	Neprilysin (EC 3.4.24.11) (Atriopeptidase) (Enkephalinase) (Neutral endopeptidase 24.11) (NEP) (Neutral endopeptidase) (Skin fibroblast elastase) (SFE) (CD antigen CD10)	*Mme*	0.541	6.612	3.872	0.007	0.114	Cell membrane; Single-pass type II membrane protein.
O09131	Glutathione S-transferase omega-1	*Gsto1 Gstx Gtsttl*	0.542	6.617	6.585	0.000	0.034	Cytoplasm, cytosol.
P0C8B4	EKC/KEOPS complex subunit GON7	*Gon7*	0.542	6.597	2.443	0.048	0.284	Nucleus {ECO:0000250|UniProtKB:Q9BXV9}.
O08797	SPI6 (Serine (Or cysteine) peptidase inhibitor, clade B, member 9)	*Serpinb9*	0.543	6.601	2.918	0.025	0.209	cytoplasma
F8WJ05	Inter-alpha-trypsin inhibitor heavy chain H1	*Itih1*	0.543	6.599	2.755	0.031	0.234	Secreted
A0A0B4J1I1	Immunoglobulin kappa variable 16-104	*Igkv16-104*	0.544	6.615	5.375	0.001	0.052	intracellular
Q99JY3	GTPase IMAP family member 4 (Immunity-associated nucleotide 1 protein) (IAN-1) (Immunity-associated protein 4)	*Gimap4 Ian1 Imap4*	0.544	6.616	6.489	0.000	0.035	Cytoplasm, cytosol.
P06684	Complement C5 (Hemolytic complement) [Cleaved into: Complement C5 beta chain; Complement C5 alpha chain; C5a anaphylatoxin; Complement C5 alpha' chain]	*C5 Hc*	0.547	6.605	3.156	0.018	0.178	Secreted.
D3Z2K2	Mitochondrial ribosomal protein S14	*Mrps14*	0.550	6.609	4.159	0.005	0.099	mittochondrion
Q9DB15	Large ribosomal subunit protein bL12m (39S ribosomal protein L12, mitochondrial) (L12mt) (MRP-L12)	*Mrpl12 Rpml12*	0.551	6.611	4.688	0.003	0.078	Mitochondrion matrix
A0A0A6YX73	cAMP-dependent protein kinase type II-alpha regulatory subunit	*Prkar2a*	0.553	6.615	5.720	0.001	0.047	Cell membrane.
Q9D7N6	Large ribosomal subunit protein uL30m (39S ribosomal protein L30, mitochondrial) (L30mt) (MRP-L30)	*Mrpl30*	0.558	6.605	3.640	0.010	0.134	Mitochondrion
Q9CQN7	Large ribosomal subunit protein mL41 (39S ribosomal protein L41, mitochondrial) (L41mt) (MRP-L41)	*Mrpl41*	0.560	6.607	3.918	0.007	0.112	Mitochondrion {ECO:0000250|UniProtKB:Q8IXM3}.
Q9D8S9	BolA-like protein 1	*Bola1*	0.562	6.598	3.028	0.021	0.194	Mitochondrion {ECO:0000250|UniProtKB:Q9Y3E2}.
Q8BVD5	MAGUK p55 subfamily member 7	*Mpp7*	0.564	6.599	3.154	0.018	0.178	Membrane; Peripheral membrane protein; Lateral cell membrane ; Cell junction; Cytoplasm.
P10107	Annexin A1	*Anxa1 Anx1 Lpc-1 Lpc1*	0.566	6.613	5.484	0.001	0.051	Nucleus; Cytoplasm ;Membrane; Early endosome; Endosome membrane ; Cell membrane ; Secreted,

Q61171	Peroxiredoxin-2	*Prdx2*	0.567	6.601	3.505	0.012	0.144	Cytoplasm {ECO:0000250|UniProtKB:P32119}.
P47968	Ribose-5-phosphate isomerase (EC 5.3.1.6) (Phosphoriboisomerase)	*Rpia Rpi*	0.567	6.587	2.530	0.042	0.267	Cytoplasm {ECO:0000250|UniProtKB:Q28960}.
Q9CQL6	Large ribosomal subunit protein bL35m (39S ribosomal protein L35, mitochondrial) (L35mt) (MRP-L35)	*Mrpl35*	0.567	6.591	2.699	0.033	0.243	Mitochondrion {ECO:0000250}.
Q8BMS4	Ubiquinone biosynthesis O-methyltransferase, mitochondrial	*Coq3*	0.574	6.608	4.619	0.003	0.081	Mitochondrion inner membrane ; Peripheral membrane protein .
A8DUK4	Beta-globin (Globin a1) (Hemoglobin, beta adult s chain) (Hemoglobin, beta adult t chain)	*Hbb-bs Glna1 Hbb-bt Hbbt1 Hbbt2*	0.576	6.606	4.188	0.005	0.098	others
Q8VHL1	Histone-lysine N-methyltransferase SETD7 (EC 2.1.1.364) (Histone H3-K4 methyltransferase SETD7) (H3-K4-HMTase SETD7) (SET domain-containing protein 7) (SET7/9)	*Setd7 Kiaa1717 Set7 Set9*	0.581	6.607	4.478	0.004	0.086	Nucleus
Q9D0T2	Dual specificity protein phosphatase 12 (EC 3.1.3.16) (EC 3.1.3.48) (Dual specificity phosphatase T-DSP4) (Dual specificity phosphatase VH1)	*Dusp12*	0.591	6.612	6.650	0.000	0.034	Nucleus; Cytoplasm, cytosol .
Q6PAL7	Transcription factor Gibbin (AT-hook DNA-binding motif-containing protein 1)	*Ahdc1*	0.592	6.608	5.267	0.002	0.056	Nucleus; Chromosome
Q64522	Histone H2A type 2-B (H2A-clustered histone 21) (H2a-613A)	*H2ac21 Hist2h2ab*	0.606	6.594	3.007	0.022	0.197	Nucleus. Chromosome.
Q91Z53	Glyoxylate reductase/hydroxypyruvate reductase (EC 1.1.1.79) (EC 1.1.1.81)	*Grhpr Glxr*	0.612	6.607	5.804	0.001	0.047	cytosol
Q00623	Apolipoprotein A-I (Apo-AI) (ApoA-I) (Apolipoprotein A1) [Cleaved into: Proapolipoprotein A-I (ProapoA-I); Truncated apolipoprotein A-I]	*Apoa1*	0.612	6.599	3.545	0.011	0.142	Secreted.
Q9D0Y8	Large ribosomal subunit protein mL52 (39S ribosomal protein L52, mitochondrial) (L52mt) (MRP-L52)	*Mrpl52*	0.615	6.591	3.155	0.018	0.178	Mitochondrion {ECO:0000250|UniProtKB:Q86TS9}.
Q8CI78	Required for meiotic nuclear division protein 1 homolog	*Rmnd1*	0.616	6.591	2.796	0.029	0.227	Mitochondrion {ECO:0000250|UniProtKB:Q9NWS8}. Note=May be localized in mitochondrial RNA granules. {ECO:0000250|UniProtKB:Q9NWS8}.
P0DOV1	Interferon-activable protein 211 (Interferon-activable protein 205-B) (Ifi-205-B) (Interferon-inducible protein p205-B) (Myeloid cell nuclear differentiation antigen) (Protein D3)	*Ifi211 Ifi205b Mnda p205*	0.618	6.577	2.543	0.042	0.265	Nucleus {ECO:0000269|PubMed:15342947}.
P97429	Annexin A4 (Annexin IV) (Annexin-4)	*Anxa4 Anx4*	0.624	6.610	9.990	0.000	0.012	Zymogen granule membrane {ECO:0000250|UniProtKB:P50994}; Peripheral membrane protein {ECO:0000250|UniProtKB:P50994}.
Q60936	Atypical kinase COQ8A, mitochondrial (EC 2.7.-.-) (Chaperone activity of bc1 complex-like) (Chaperone-ABC1-like) (Coenzyme Q protein 8A) (aarF domain-containing protein kinase 3)	*Coq8a Adck3 Cabc1*	0.631	6.607	7.159	0.000	0.028	Mitochondrion {ECO:0000250|UniProtKB:Q8NI60}. Membrane {ECO:0000255}; Single-pass membrane protein {ECO:0000250|UniProtKB:Q8NI60, ECO:0000255}.
Q91VB8	Alpha globin 1 (Alpha globin 2) (Alpha-globin) (Globin c1) (Hemoglobin alpha, adult chain 1) (Hemoglobin alpha, adult chain 2)	*Hba-a1 Glnc1 haemaglobin alpha 1 haemaglobin alpha 2 Hba-a2*	0.631	6.586	3.076	0.020	0.189	others
P26187	Methylated-DNA--protein-cysteine methyltransferase (EC 2.1.1.63) (6-O-methylguanine-DNA methyltransferase) (MGMT) (O-6-methylguanine-DNA-alkyltransferase)	*Mgmt*	0.636	6.581	2.819	0.028	0.226	Nucleus
Q8CI08	SLAIN motif-containing protein 2	*Slain2 Kiaa1458*	0.637	6.586	2.815	0.029	0.226	Cytoplasm, cytoskeleton .
Q9CQP0	Large ribosomal subunit protein bL33m (39S ribosomal protein L33, mitochondrial) (L33mt) (MRP-L33)	*Mrpl33*	0.646	6.605	6.993	0.000	0.029	Mitochondrion
Q9CWG8	Protein arginine methyltransferase NDUFAF7, mitochondrial (EC 2.1.1.320) (NADH dehydrogenase [ubiquinone] complex I, assembly factor 7) (Protein midA homolog)	*Ndufaf7*	0.662	6.597	4.741	0.003	0.077	Mitochondrion {ECO:0000250|UniProtKB:Q7L592}.

Q8R1S0	Ubiquinone biosynthesis monooxygenase COQ6, mitochondrial (EC 1.14.13.-) (Coenzyme Q10 monooxygenase 6)	*Coq6*	0.669	6.604	8.468	0.000	0.016	Mitochondrion inner membrane ; Peripheral membrane protein ; Matrix side ; Golgi apparatus.
Q9CWU6	Ubiquinol-cytochrome c reductase complex assembly factor 1	*Uqcc1*	0.673	6.598	5.647	0.001	0.048	Mitochondrion inner membrane ; Cytoplasmic
Q8K0Q5	Rho GTPase-activating protein 18 (Rho-type GTPase-activating protein 18)	*Arhgap18*	0.690	6.596	5.762	0.001	0.047	Cytoplasm {ECO:0000250|UniProtKB:Q8N392}.
A2AQP0	Myosin-7B (Myosin cardiac muscle beta chain) (Myosin heavy chain 7B, cardiac muscle beta isoform)	*Myh7b*	0.694	6.575	3.199	0.017	0.174	Membrane; Peripheral membrane protein
Q9DBB8	Trans-1,2-dihydrobenzene-1,2-diol dehydrogenase	*Dhdh*	0.699	6.559	2.755	0.031	0.234	others
Q61097	Kinase suppressor of Ras 1 (mKSR1) (EC 2.7.11.1) (Protein Hb)	*Ksr1 Ksr*	0.700	6.602	10.651	0.000	0.011	Cytoplasm Membrane ;Peripheral membrane protein; Cell membrane ; Peripheral membrane protein;Endoplasmic reticulum membrane
Q78J03	Methionine-R-sulfoxide reductase B2, mitochondrial (MsrB2) (EC 1.8.4.12) (EC 1.8.4.14)	*Msrb2*	0.701	6.590	4.809	0.003	0.074	Mitochondrion
Q9CPW3	Large ribosomal subunit protein mL54 (39S ribosomal protein L54, mitochondrial) (L54mt) (MRP-L54)	*Mrpl54*	0.702	6.597	6.190	0.001	0.039	Mitochondrion
Q9CYL5	Golgi-associated plant pathogenesis-related protein 1	*Glipr2*	0.704	6.596	6.484	0.000	0.035	Golgi apparatus membrane; Lipid-anchor.
Q8BGG7	Ubiquitin-associated and SH3 domain-containing protein	*Ubash3b*	0.713	6.586	4.179	0.005	0.098	Cytoplasm, Nucleus .
E9Q0X9	Methyltransferase like 17	*Mettl17*	0.720	6.595	6.890	0.000	0.030	Mitochondrion
E9PZ91	Bis(5'-adenosyl)-triphosphatase (EC 3.6.1.29)	*Fhit*	0.729	6.597	9.540	0.000	0.013	Mitochondrion;Nucleus;Cytoplasm
Q01149	Collagen alpha-2(I) chain (Alpha-2 type I collagen)	*Col1a2 Cola2*	0.730	6.571	3.357	0.014	0.157	Secreted, extracellular space, extracellular matrix.
Q9CQA9	Cancer-related nucleoside-triphosphatase	*Ntpcr*	0.741	6.593	7.052	0.000	0.029	others
Q8BJ03	Heme A synthase COX15 (HAS) (EC 1.17.99.9) (Cytochrome c oxidase assembly protein COX15 homolog)	*Cox15*	0.744	6.576	3.733	0.009	0.125	Mitochondrion inner membrane ; Multi-pass membrane protein .
Q8R3K3	Pentatricopeptide repeat-containing protein 2, mitochondrial	*Ptcd2*	0.746	6.564	3.320	0.015	0.162	Mitochondrion
A0A087WQF7	Solute carrier family 39 (zinc transporter), member 10	*Slc39a10*	0.746	6.562	2.736	0.032	0.236	Multi-pass membrane protein
D3Z4C9	Mitochondrial nucleoid factor 1 (Mitochondrial protein M19)	*Uqcc2 2900010M23Rik Mnf1*	0.757	6.592	7.499	0.000	0.025	Mitochondrion matrix, mitochondrion nucleoid.
Q61703	Inter-alpha-trypsin inhibitor heavy chain H2 (ITI heavy chain H2) (ITI-HC2) (Inter-alpha-inhibitor heavy chain 2)	*Itih2*	0.760	6.537	2.485	0.045	0.274	Secreted.
P32037	Solute carrier family 2, facilitated glucose transporter member 3 (Glucose transporter type 3, brain) (GLUT-3)	*Slc2a3 Glut3*	0.763	6.586	5.715	0.001	0.047	Cell membrane ; Multi-pass membrane protein ;Perikaryon .
Q9DBF1	Alpha-aminoadipic semialdehyde dehydrogenase	*Aldh7a1 Ald7a1*	0.789	6.586	6.602	0.000	0.034	Cytoplasm; Nucleus ;Mitochondrion
Q9Z2D6	Methyl-CpG-binding protein 2 (MeCp-2 protein) (MeCp2)	*Mecp2*	0.806	6.546	3.287	0.015	0.166	Nucleus
Q5PR72	Phosphodiesterase (EC 3.1.4.-)	*Pde2a*	0.833	6.581	9.055	0.000	0.013	others
G3X957	PTPRF interacting protein, binding protein 2 (liprin beta 2)	*Ppfibp2*	0.871	6.567	5.985	0.001	0.042	cytsol
D3Z2M5	BCL2 binding component 3	*Bbc3*	1.055	6.544	8.665	0.000	0.015	mitochondrion
H3BKG0	Caveolin 1, caveolae protein	*Cav1*	1.175	6.493	4.064	0.006	0.104	Golgi apparatus membrane

**Table S2** Primers used in this study				
	application	Forward	Reverse	probe
mouse Pcbp1 primer	real-time PCR	GACGCCGGTGTGACTGAAA	GTCAGCGTGATGATCCTCTCC	
mouse IgM primer (Total)	real-time PCR	CAGGGGTCTCACCTTCTTGA	AGGTTAGCGGACTTGCTGAG	
mouse IgD primer	real-time PCR	CCAAGATGACATCTCAGG	CTCCAAGCAAAGAATCAC	
mouse Actin	real-time PCR	CGGTTCCGATGCCCTGAGGCTCTT	CGTCACACTTCATGATGGAATTGA	
mouse IgM primer (membrane)	real-time PCR	TCCGGAGAGACCTATACCTGTGTT	TTCTCAAAGCCTTCCTCCTCAGCA	
mouse IgM primer (secreted)	real-time PCR	TCTCCCTGATCATGTCTGACACAG	ATACACAGAGCAACTGGACACCCA	
mouse Fdxr primer	real-time PCR	TGTGGGCTGGTACAATGGAC	GGCCCACTATCCACACAGTC	
28S primer	RIP	CTAAATACCGGCACGAGACC	TTCACGCCCTCTTGAACTCT	
FDXR 3'UTR primer	RIP	CAAGACAAGAGCCGGACAGT	TCTAGAGGCCCTCCAAGGTC	
mouse IgM-digital primer	digital PCR	CAATGTGTCCGTCGTGGATCT	GTCCTCAGTGTAGCCCAAGATG	5'(FAM) CGTGCCGCCTGGAGAAACCTGCC 3'(BHQ1)
mouse Gapdh-digital primer	digital PCR	CAGGGGTCTCACCTTCTTGA	AGGTTAGCGGACTTGCTGAG	5'(VIC) CTCCACATGTGCTGCCAGTCCCTCCACAGA 3'(BHQ1)
human sh*Pcbp1* #1		TAGTCTGGCCCAGTATCTAAT		
human sh*Pcbp1* #2		CGGGTGTAAGATCAAAGAGAT		
human sh*Fdxr* #1		AGCTTCGGGAGATGATTCAGT		
human sh*Fdxr* #2		TGCTCAGCAGCATTGGGTATA		

**Table 3** Sequences used in this study
**Human *Pcbp1* -cDNA**
ATGGATGCCGGTGTGACTGAAAGTGGACTAAATGTGACTCTCACCATTCGGCTTCTTATGCACGGAAAGGAAGTAG<br>GAAGCATCATTGGGAAGAAAGGGGAGTCGGTTAAGAGGATCCGCGAGGAGAGTGGCGCGCGGATCAACATCTCGG<br>AGGGGAATTGTCCGGAGAGAATCATCACTCTGACCGGCCCCACCAATGCCATCTTTAAGGCTTTCGCTATGATCATC<br>GACAAGCTGGAGGAAGATATCAACAGCTCCATGACCAACAGTACCGCGGCCAGCAGGCCCCCGGTCACCCTGAGG<br>CTGGTGGTGCCGGCCACCCAGTGCGGCTCCCTGATTGGGAAAGGCGGGTGTAAGATCAAAGAGATCCGCGAGAGT<br>ACGGGGGCGCAGGTCCAGGTGGCGGGGGATATGCTGCCCAACTCCACCGAGCGGGCCATCACCATCGCTGGCGT<br>GCCGCAGTCTGTCACCGAGTGTGTCAAGCAGATTTGCCTGGTCATGCTGGAGACGCTCTCCCAGTCTCCGCAAGGG<br>AGAGTCATGACCATTCCGTACCAGCCCATGCCGGCCAGCTCCCCAGTCATCTGCGCGGGCGGCCAAGATCGGTGC<br>AGCGACGCTGCGGGCTACCCCCATGCCACCCATGACCTGGAGGGACCACCTCTAGATGCCTACTCGATTCAAGGA<br>CAACACACCATTTCTCCGCTCGATCTGGCCAAGCTGAACCAGGTGGCAAGACAACAGTCTCACTTTGCCATGATGCA<br>CGGCGGGACCGGATTCGCCGGAATTGACTCCAGCTCTCCAGAGGTGAAAGGCTATTGGGCAAGTTTGGATGCATCT<br>ACTCAAACCACCCATGAACTCACCATTCCAAATAACTTAATTGGCTGCATAATCGGGCGCCAAGGCGCCAACATTAA<br>TGAGATCCGCCAGATGTCCGGGGCCCAGATCAAAATTGCCAACCCAGTGGAAGGCTCCTCTGGTAGGCAGGTTACT<br>ATCACTGGCTCTGCTGCCAGTATTAGTCTGGCCCAGTATCTAATCAATGCCAGGCTTTCCTCTGAGAAGGGCATGGG<br>GTGCAGC
**Human Pcbp1-Δiron protein**
MDAGVTESGLNVTLTIRLLMHGKEVGSIIGKKGESVKRIREESGARINISEGNCPERIITLTGPTNAIFKAFAMIIDKLEE**A**INS<br>SMTNSTAASRPPVTLRLVVPATQCGSLIGKGGCKIKEIRESTGAQVQVAGDMLPNSTERAITIAGVPQSVTECVKQICLVM<br>L**A**TLSQSPQGRVMTIPYQPMPASSPVICAGGQDRCSDAAGYPHATHDLEGPPLDAYSIQGQHTISPLDLAKLNQVARQQ<br>SHFAMMHGGTGFAGIDSSSPEVKGYWASLDASTQTTHELTIPNNLIGCIIGRQGANINEIRQMSGAQIKIANPVEGSSGRQ<br>VTITGSAASISLAQYLINARLSS**A**KGMGCS
**Human Pcbp1-ΔRNA protein**
MDAGVTESGLNVTLTIRLLMHGKEVGSIIGKKGESVKRI**A**EESGARINISEGNCPERIITLTGPTNAIFKAFAMIIDKLEEDINS<br>SMTNSTAASRPPVTLRLVVPATQCGSLIGKGGCKIKEI**A**ESTGAQVQVAGDMLPNSTERAITIAGVPQSVTECVKQICLVM<br>LETLSQSPQGRVMTIPYQPMPASSPVICAGGQDRCSDAAGYPHATHDLEGPPLDAYSIQGQHTISPLDLAKLNQVARQQ<br>SHFAMMHGGTGFAGIDSSSPEVKGYWASLDASTQTTHELTIPNNLIGCIIGRQGANINEI**A**QMSGAQIKIANPVEGSSGRQ<br>VTITGSAASISLAQYLINARLSSEKGMGCS
**Human Fdxr-cDNA**
ATGGCTTCGCGCTGCTGGCGCTGGTGGGGCTGGTCGGCGTGGCCTCGGACCCGGCTGCCTCCCGCCGGGAGCAC<br>CCCGAGCTTCTGCCACCATTTCTCCACACAGGAGAAGACCCCCCAGATCTGTGTGGTGGGCAGTGGCCCAGCTGG<br>CTTCTACACGGCCCAACACCTGCTAAAGCACCCCCAGGCCCACGTGGACATCTACGAGAAACAGCCTGTGCCCTTT<br>GGCCTGGTGCGCTTTGGTGTGGCGCCTGATCACCCCGAGGTGAAGAATGTCATCAACACATTTACCCAGACGGCCC<br>ATTCTGGCCGCTGTGCCTTCTGGGGCAACGTGGAGGTGGGCAGGGACGTGACGGTGCCGGAGCTGCAGGAGGCC<br>TACCACGCTGTGGTGCTGAGCTACGGGGCAGAGGACCATCGGGCCCTGGAAATTCCTGGTGAGGAGCTGCCAGGT<br>GTGTGCTCCGCCCGGGCCTTCGTGGGCTGGTACAACGGGCTTCCTGAGAACCAGGAGCTGGAGCCAGACCTGAGC<br>TGTGACACAGCCGTGATTCTGGGGCAGGGGAACGTGGCTCTGGACGTGGCCCGCATCCTACTGACCCCACCTGAG<br>CACCTGGAGAGAACGGACATCACGAAGGCAGCCCTGGGTGTACTGAGGCAGAGTCGAGTGAAGACAGTGTGGCTA<br>GTGGGCCGGCGTGGACCCCTGCAAGTGGCCTTCACCATTAAGGAGCTTCGGGAGATGATTCAGTTACCGGGAGCC<br>CGGCCCATTTTGGATCCTGTGGATTTCTTGGGTCTCCAGGACAAGATCAAGGAGGTCCCCCGCCCGAGGAAGCGG<br>CTGACGGAACTGCTGCTTCGAACGGCCACAGAGAAGCCAGGGCCGGCGGAAGCTGCCCGCCAGGCATCGGCCTC<br>CCGTGCCTGGGGCCTCCGCTTTTTCCGAAGCCCCCAGCAGGTGCTGCCCTCACCAGATGGGCGGCGGGCAGCAG<br>GTGTCCGCCTAGCAGTCACTAGACTGGAGGGTGTCGATGAGGCCACCCGTGCAGTGCCCACGGGAGACATGGAAG<br>ACCTCCCTTGTGGGCTGGTGCTCAGCAGCATTGGGTATAAGAGCCGCCCTGTCGACCCAAGCGTGCCCTTTGACTC<br>CAAGCTTGGGGTCATCCCCAATGTGGAGGGCCGGGTTATGGATGTGCCAGGCCTCTACTGCAGCGGCTGGGTGAA<br>GAGAGGACCTACAGGTGTCATAGCCACAACCATGACTGACAGCTTCCTCACCGGCCAGATGCTGCTGCAGGACCTG<br>AAGGCTGGGTTGCTCCCCTCTGGCCCCAGGCCTGGCTACGCAGCCATCCAGGCCCTGCTCAGCAGCCGAGGGGTC<br>CGGCCAGTCTCTTTCTCAGACTGGGAGAAGCTGGATGCCGAGGAGGTGGCCCGGGGCCAGGGCACGGGGAAGCC<br>CAGGGAGAAGCTGGTGGATCCTCAGGAGATGCTGCGCCTCCTGGGCCAC
**Murine Fdxr-cDNA**

ATGGCTCCTCGCTGCTGGCACTGGTGGCGCTGGTCCGCGTGGTCTGGGCTTCGGCCGTCTCCCTCCAGGAGCACT
CCGACCCCAGGCTTCTGCCAGAAGTTCTCCACACAGGAGAAGACCCCTCAGATCTGTGTGGTCGGCAGTGGCCCA
GCTGGCTTCTACACAGCCCAACACTTGTTAAAGCACCACACCCATGCCCACGTAGACATCTACGAGAAGCAGCTCG
TGCCATTCGGCCTGGTGCGCTTTGGTGTGGCACCTGACCATCCTGAAGTAAAGAATGTTATCAACACATTTACACAG
ACAGCCCGCTCAGACCGCTGTGCCTTCCAGGGCAATGTGGTGGTGGGCAGGGACGTGTCGGTTCCAGAGCTTCGG
GAAGCCTACCATGCTGTGGTGCTGAGTTATGGAGCAGAGGACCACCAACCCCTGGGAATTCCTGGCGAGGAGCTG
CCTGGAGTGGTCTCAGCCCGGGCCTTTGTGGGCTGGTACAATGGACTTCCCGAGAACCAGGAGCTGGCGCCAGAT
CTGAGCTGTGACACGGCTGTAATTCTGGGACAGGGGAATGTGGCTCTGGATGTGGCCCGGATCCTGCTGACCCCA
CCTGAGCACCTGGAGAAAACAGACATCACAGAGGCTGCATTGGGGGCCCTGAGGCAGAGTCGGGTGAAGACTGTG
TGGATAGTGGGCCGGCGTGGGCCCTTGCAAGTAGCGTTCACCATTAAGGAGCTTCGGGAGATGATTCAGTTGCCAG
GAACCCGGCCCATTTTGGATCCTTCGGATTTCTTGGGCCTCCAGGACAGAATTAAGGATGTCCCCCGTCCAAGGAG
GCGGCTAACAGAACTGCTGCTTCGGACAGCCACGGAGAAGCCAGGAGTGGAAGAGGCTGCCCGCCAGGCACTGG
CCTCCCGGGCCTGGGGTCTCCGCTTTTTCCGAAGCCCCCAGCAGGTGCTCCCTACCCCAGATGGCCAACGGGTAG
CAGGCATCCGCCTGGCAGTTACTAGTCTAGAGGGTGTTGGGGAGTCCACTCGGGCAGTGCCCACAGGAGACGTGG
AGGACCTCCCTTGTGGACTGCTGCTGAGCAGCGTTGGGTATAAGAGCCGCCCCATCGACCCCAGCGTGCCCTTTGA
CCCCAAGCTTGGAGTCATCCCCAACACAGAGGGCCGGGTTGTGAATGTCCCAGGCCTCTACTGCAGTGGCTGGGT
GAAGAGGGGACCCACAGGTGTCATCACCACAACCATGACAGACAGCTTCCTCACCAGCCAGGCGCTGCTGGAGGA
CCTGAAGGCGGGGCTGCTGCCCTCCGGCCCCAGACCCGGCTATGTGGCCATTCAAGCCCTGCTCAGCAATCGAGG
AGTCCGGCCAGTGTCTTTCTCAGACTGGGAGAAGCTGGATGCTGAGGAAGTCTCTCGAGGCCAAGGTACTGGGAA
ACCAAGGGAGAAGCTGGTGGATCGAAGAGAGATGCTGCGGCTGCTGGGCCACTGA

Murine Fdxr 3'UTR WT

ACCTGGGCCCCAGCCCCTACCGTGCAAGACAAGAGCCGGACAGTCTGGGTTGGACTCTTCTCTCGTTCATTATGTT
ATTGACGGCCCTGGCTTAGACGCCGAACCGTGCCCTTTCCAGGGGCCTCTGAGCTCTGCCTTCTGCAAGCTGACCT
CCCAGTGTGGCTTGAGGCCAAGGAGGGAACCTGAAGCTAGGGATGGATGGAGATACAGACTGACCCTACCCGTCC
TGCTTCCTTTCTGATGGACCTTGGAGGGCCTCTAGAACGGAACACAGTGGAAATAAAACAGATGTGCCTAAGAcccat
ggctgatagctaccatttttggggggctgggggtgctgttactgtgtagctcctgtttgcctgtggctgccagagggaactaagaaaacagatccttgctcaagagtgtcctgg
ggacagtggggattgggggggagggggctgcagtggggatgggggcagggcatacacctatcctccctttcctgtctttcctgcttcttcggcc

Murine Fdxr 3'UTR ΔCA mutant

ACCTGGGGGGGAGGGGGTACCGTGCAAGACAAGAGCCGGACAGTCTGGGTTGGACTCTTCTCTCGTTCATTATGTT
ATTGACGGCCCTGGCTTAGACGGGGAAGGGTGGGGTTTCCAGGGGCCTCTGAGCTCTGCCTTCTGCAAGCTGACC
TCCCAGTGTGGCTTGAGGCCAAGGAGGGAACCTGAAGCTAGGGATGGATGGAGATACAGACTGAGGGTAGGGGTG
GTGCTTCCTTTCTGATGGACCTTGGAGGGCCTCTAGAACGGAACACAGTGGAAATAAAACAGATGTGGGTAAGAGG
Gatggctgatagctaccatttttggggggctgggggtgctgttactgtgtagctcctgtttgcctgtggctgccagagggaactaagaaaacagatccttgctcaagagtgtc
ctggggacagtggggattgggggggagggggctgcagtggggatgggggcagggcatacacctatGGtGGGtttGGtgtctttcctgcttcttcggcc

Table S4 Seahorse data

OCAR- in naive B cells(Fig.2G)						
Time (minutes)	WT			KO		
1.308903	61.37133	61.558037	60.747715	52.339758	52.206815	50.153398
7.767699	56.00884	56.374641	54.665247	49.237723	49.966376	48.035511
14.22741	53.738296	52.517138	52.009134	47.402295	48.600091	46.87409
20.769892	27.489031	27.349298	26.582477	22.131967	20.427569	28.221999
27.230124	23.058127	22.300061	20.790563	17.187182	18.009783	17.107312
33.687863	22.465063	21.727093	19.78421	17.301628	18.038319	17.555353
40.232651	93.890844	90.73805	92.492912	71.232037	75.854851	73.065995
46.70101	81.332272	78.850709	78.279899	63.084813	68.120485	61.805965
53.16286	74.09384	72.709768	71.359449	60.744341	64.751337	57.568354
59.702856	15.966724	13.921722	13.963082	11.469695	11.848717	12.907991
66.171255	16.531168	13.922242	14.410808	11.522553	12.585596	12.989073
72.642565	16.666228	13.378099	14.164118	11.878356	12.199043	12.289226

ECAR- in naive B cells(Fig.2I)				
Time (minutes)	WT		KO	
1.305745	5.469424	5.6874078	6.5789665	6.769599
7.758887	5.40206675	5.752687	6.35060475	6.6012255
14.2048	5.200239	5.6808246	6.19952575	6.813855
20.739768	8.12179825	8.930468	12.187996	11.6228823
27.18912	8.27445675	8.853757	12.42137275	12.0467243
33.640104	8.351668	8.7764372	12.222536	11.9334508
40.180235	10.50490475	10.6241566	12.289887	11.5713518
46.637721	11.09726825	11.2454128	11.69772625	11.377316
53.097647	11.80555	11.5581562	11.26276225	11.1404708
59.637214	3.60805575	3.7667726	3.837679	4.234468
66.098716	2.55145525	2.754825	3.27622975	3.8567195
72.560475	2.686758	2.5562386	3.46180675	3.90724425

ECAR- in iGCB cells (Fig.4G)						
Time (minutes)	WT			KO		
1.305745	21.60959767	21.9869772	22.371548	31.5842118	29.231958	27.401394
7.758887	20.4722835	21.2219028	21.4875874	30.6434352	28.504526	26.330063
14.2048	20.17785533	20.5271654	20.6994172	29.839023	27.4551588	26.042595
20.739768	102.1731348	99.2596284	101.8533606	126.762377	120.82184	116.97884
27.18912	118.7339732	120.037458	120.0868278	144.727514	139.294132	134.39424
33.640104	119.4182425	122.305288	120.4760156	144.162291	138.49111	134.59186
40.180235	140.2945955	142.645479	139.4151204	166.396218	165.589088	163.3387
46.637721	134.4502402	139.122749	134.432246	160.921105	155.624725	153.78393
53.097647	130.201374	135.377418	130.32713	156.765688	150.6541	148.31632
59.637214	29.5435905	30.3525318	27.6742658	37.5221632	33.744682	34.258093
66.098716	21.90858667	23.8203096	21.3588548	31.7921982	28.0053152	27.629675
72.560475	20.95724567	22.3447836	20.0055972	29.9874474	26.6498704	25.996291

OCAR- in iGCB cells (Fig.4F)								
Time (minutes)	WT							
1.30996	158.449834	160.032698	162.382507	136.105377	153.650306	159.09802	149.208414	137.448962
7.7611	153.669599	154.502069	157.278243	134.002748	151.361497	155.23569	144.889797	136.050377
14.205038	155.245219	155.421105	158.014103	135.328913	153.033036	155.68909	147.20346	137.67625
20.736602	65.477222	67.165014	68.314795	54.00199	63.368489	66.26084	62.495987	58.102605
27.187284	67.337945	68.014549	70.874546	55.279571	68.811334	70.373671	66.987257	60.796029
33.634446	71.353049	71.966696	74.32113	58.274928	73.592313	75.177146	70.973891	65.29298
40.158457	385.987289	393.135492	386.423749	376.196244	425.721697	440.40533	406.411935	409.495692
46.609079	420.21491	420.230832	423.735802	420.749944	422.364541	436.8241	406.537149	413.586057
53.061414	418.376855	415.52866	420.616858	419.837619	418.327346	431.64649	400.996407	409.92365
59.597592	31.791926	34.465704	35.062503	20.823456	25.995241	27.867145	26.037133	23.21595
66.055455	32.786752	35.836734	35.81193	25.381656	29.522941	32.257032	31.311645	27.208528
72.509276	33.810611	34.652008	34.881259	25.895506	30.572881	32.833194	30.632792	27.504464
Time (minutes)	KO							
1.30996	97.967972	100.082365	99.407988	97.527466	95.277222	106.4997	104.663552	102.042548
7.7611	96.748883	97.177595	95.732492	96.276671	94.474474	104.7055	102.750852	101.438011
14.205038	98.322032	99.690329	98.732031	97.826233	96.348171	106.36715	106.18764	103.420008
20.736602	37.715698	36.971583	37.572882	36.696695	34.561742	39.873296	38.621516	36.279754
27.187284	39.782437	39.507612	40.89649	38.702129	36.139906	40.956993	41.289782	38.631063
33.634446	42.649968	41.262333	42.547515	41.450381	39.053391	44.349503	43.917639	40.994732
40.158457	242.290679	235.165101	240.034767	240.566244	240.152427	227.84839	243.37627	245.836949
46.609079	232.550853	227.721429	228.309587	225.163559	232.484845	218.62122	227.966003	233.143727
53.061414	227.544942	223.209818	222.181132	221.032637	226.493584	215.99652	218.527575	227.807577
59.597592	20.435685	18.468481	20.737945	18.576735	15.620768	21.487576	21.608085	19.263256
66.055455	21.540232	21.450363	24.323375	21.305376	19.09064	23.694291	23.698506	21.530908
72.509276	21.404209	20.926322	24.455533	20.646324	19.391027	23.95501	24.745448	20.610714
